# A macrocyclic oligofuran: synthesis, solid state structure and electronic properties†
†Electronic supplementary information (ESI) available. CCDC 1892379. For ESI and crystallographic data in CIF or other electronic format see DOI: 10.1039/c9sc03247a


**DOI:** 10.1039/c9sc03247a

**Published:** 2019-08-19

**Authors:** Sandip V. Mulay, Or Dishi, Yuan Fang, Muhammad R. Niazi, Linda J. W. Shimon, Dmitrii F. Perepichka, Ori Gidron

**Affiliations:** a Institute of Chemistry , The Hebrew University of Jerusalem , Edmond J. Safra Campus , Jerusalem , Israel . Email: ori.gidron@mail.huji.ac.il; b Department of Chemistry , McGill University , Montreal , QC H3A 0B8 , Canada . Email: dmitrii.perepichka@mcgill.ca; c Chemical Research Support Unit , Weizmann Institute of Science , Rehovot , Israel

## Abstract

We report the first π-conjugated macrocyclic system with an oligofuran backbone. The calculated HOMO–LUMO gap is similar to that of the corresponding linear polymer, indicating a remarkable electron delocalization. The X-ray structure reveals a planar conformation, in contrast to the twisted conformation of macrocyclic oligothiophenes. The intermolecular π–π stacking distance is extremely small (3.17 Å), indicating very strong interactions. The macrocycle forms large π-aggregates in solution and shows a tendency toward highly ordered multilayer adsorption at the solid–liquid interface. The face-on orientation of molecules explains the higher hole mobility observed in the out-of-plane direction.

## Abstract 2

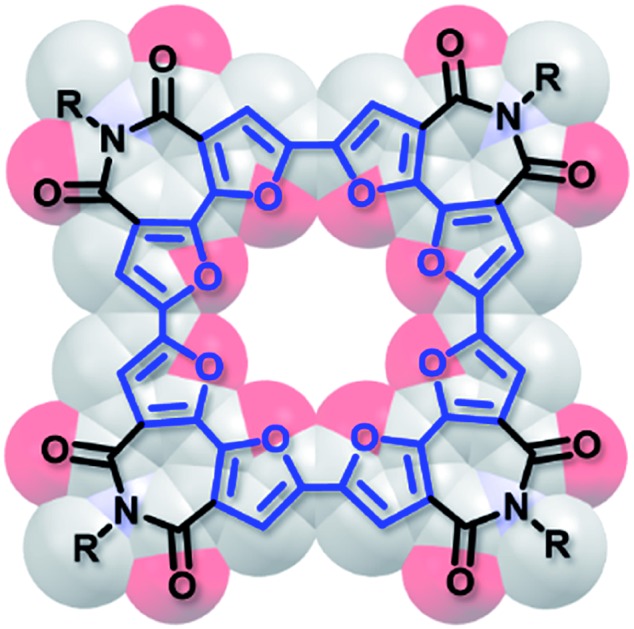
The first π-conjugated macrocyclic system with an oligofuran backbone display planar conformation and forms large π-aggregates, in contrast to the twisted conformation of small macrocyclic oligothiophenes.

## Introduction

π-Conjugated macrocycles with well-defined diameters have received significant attention in the last few decades, mainly because of their unique optical and electronic properties, their host–guest capabilities, and their potential as building blocks for supramolecular materials.^1^–^6^ Many π-conjugated macrocyclic systems aggregate into columnar structures,^4^,^7^–^11^ producing molecular channels that, with the appropriate inner-core functionalization, may give rise to ion channels, rod-like micellar aggregates, and even reaction chambers.^12^–^15^

**Chart 1:**
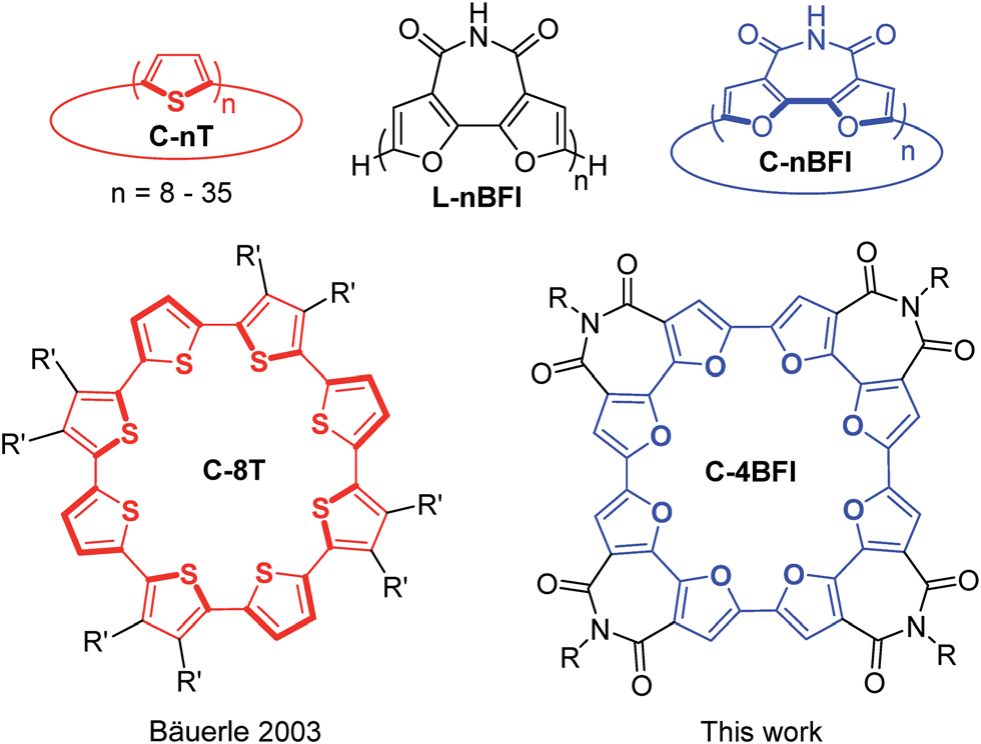
Structures of the linear (L) and macrocyclic (C) α,α′-oligofurans (**nBFI**) and α,α′-oligothiophenes (**nT**) discussed in this work. *R*′ = *n*-butyl, *R* = 2-octyldodecyl.

A particularly intriguing class of π-conjugated macrocycles is macrocyclic oligothiophenes (**C-nT**, Chart 1), introduced by Bäuerle's group in sizes ranging from **C-8T** to **C-35T**.^16^–^18^ These macrocycles demonstrate interesting electronic and optical properties,^19^ self-assembly at the solid–liquid interface,^20^–^22^ and stable redox behavior.^23^ Subsequently, Iyoda's group found that, when separated by acetylene spacers, thiophenes can form giant macrocyclic oligomers that arrange themselves into wire-like assemblies and form inclusion complexes with fullerenes.^24^–^26^ However, whereas the Iyoda's thienyleneethynylene macrocycles are planar, pure thiophene-based macrocycles, **C-nT**, in small diameters are distorted. Although no crystallographic information is available for **C-8T**, DFT calculations indicate that it adopts a spiderlike conformation^27^ and X-ray analysis of the larger **C-10T** reveals interring dihedral twisting of 26–34°.^23^,^28^ Such twisting may negatively affect both intramolecular π-conjugation and intermolecular interactions, which are crucial for optoelectronic applications.

We have previously introduced linear α-oligofurans, which display high planarity/rigidity, good π-conjugation, and strong fluorescence compared with their thiophene analogs.^29^–^32^ Our calculations predicted that, in contrast to **C-nT**, small macrocyclic oligofurans (6- to 8-mers) should be planar, as the formed bisecting angle for each furan is 125° *vs.* 150° for thiophene, leading to low strain energies, lower HOMO–LUMO gaps, and stronger π-conjugation.^33^ Moreover, as macrocyclic oligofurans may be considered ‘conjugated crown ethers’, they are potentially interesting as host–guest systems or as components in supramolecular structures (rotaxenes, catenanes, *etc.*).^34^–^37^ However, despite the plethora of reported thiophene-based macrocycles,^1^,^38^ macrocyclic oligofurans are not known, possibly because the relative instability of oligofurans has hampered their development.^39^ To overcome this instability, we recently introduced 2,2′-bifuran-3,3′-dicarboximide oligomers and polymers (**L-nBFI**, Chart 1), which are significantly more stable than the parent furan.^40^ Since each **BFI** unit consists of two furans ‘locked’ in a *syn* orientation, and such preorganization is expected to favor macrocyclization by reducing strain energy,^41^ we envisioned that **BFI** could serve as a building unit for macrocyclic oligofuran scaffolds.

Here we describe the synthesis, structure, and properties of the first macrocycle having an oligofuran backbone, the α,α′-tetramer of 2,2′-bifuran-3,3′dicarboximide **C-4BFI** (Chart 1). In accordance with our calculations and crystallographic analysis, **C-4BFI** is planar and exhibits remarkably strong intermolecular interactions. In solution and as a solid, **C-4BFI** self-assembles *via* π-stacking. Scanning tunneling microscopy (STM) imaging reveals the formation of ordered multilayers at the solid–liquid interface. The face-on orientation of the molecules on the surface explains the observed charge transport anisotropy with higher mobility in out-of-plane direction.

## Results and discussion

**Fig. 1:**
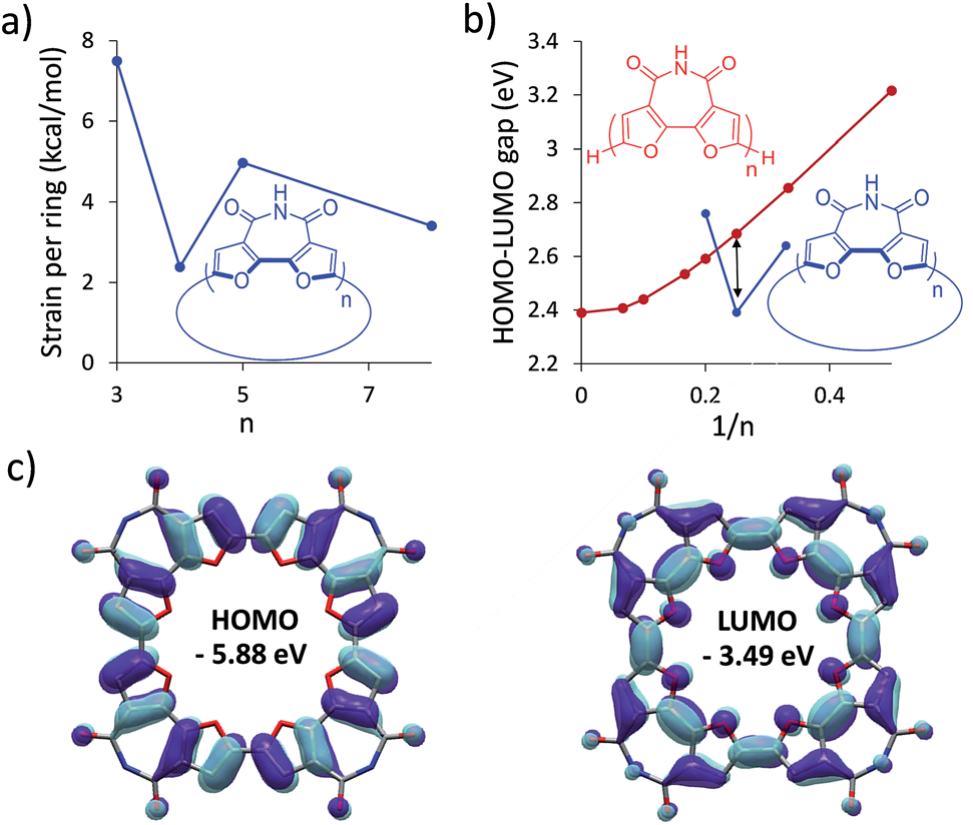
Calculated (B3LYP/6-311G(d)) (a) strain energy per unit for **C-nBFI**; (b) HOMO–LUMO gap for **L-nBFI** (red) and C-nBFI (blue), where the black arrow indicates the tetramer; and (c) the HOMO and LUMO surfaces of **C-4BFI**.

To find the ideal candidate for macrocyclization, we first calculated the electronic structures (at the DFT/B3LYP/6-311G(d) level) of **C-nBFI** molecules of various sizes (Fig. 1a). We found that the tetramer, **C-4BFI**, has the lowest strain energy (2.4 kcal mol^–1^ per bifuran unit), being lower than that of the smaller **C-3BFI** (7.5 kcal mol^–1^) and of the larger **C-5BFI** (5.0 kcal mol^–1^). In addition, the backbone of **C-4BFI** is predicted to be planar which maximizes the π-conjugation.^42^ Calculations predicted that the HOMO–LUMO gap for **C-4BFI** (2.39 eV) is 0.3 eV lower than that of the linear tetramer (**L-4BFI**), and is almost identical to the bandgap of the linear polymer (Fig. 1b). The predicted HOMO–LUMO gap in **C-3BFI** is also smaller compared with the linear analogue **L-3BFI** (by 0.2 eV) but the gap of **C-5BFI** macrocycle is larger by 0.18 eV than the gap of **L-5BFI**, which is attributed to its non-planarity. Both the HOMO and LUMO resemble those of the parent macrocyclic furans, with a small contribution from the imide group to the LUMO (Fig. 1c). The computational results thus reveal **C-4BFI** as an optimum target for cyclization.

**Scheme 1:**
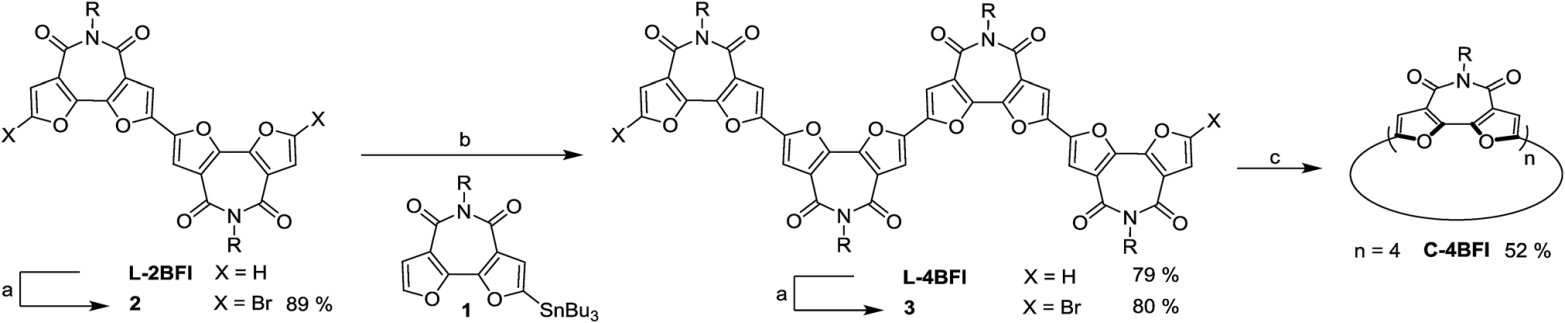
Synthesis of **C-4BFI** Conditions: (a) Br_2_, FeCl_3_, CH_2_Cl_2_, rt, dark; (b) Pd(PPh_3_)_4_, toluene, 90 °C; (c) Ni(COD)_2_, 2,2′-bipyridine, THF, 50 °C, 48 h.

The synthesis started with preparation of the linear tetramer **L-4BFI** by Stille coupling of stannane **1** with the dibrominated product of **L-2BFI** (**2**) (Scheme 1).^40^ The 2-octyldodecyl side chains were introduced for solubility (attempts to use *n*-hexyl side chain resulted in insoluble linear tetramer). The coupling product, **L-4BFI**, was then brominated using Br_2_ in the presence of FeCl_3_ to yield **3**. Finally, macrocyclization was performed by adding a stoichiometric amount of Ni(COD)_2_ and 2,2′-bipyridine to a dilute solution (5 × 10^–4^ M) of **3** at 50 °C to yield **C-4BFI** (52%) after 48 h.^43^ The macrocycle was characterized using NMR and by MALDI-TOF (see ESI†). Cyclic voltammetry (CV) of **C-4BFI** shows a quasi-reversible oxidation peak at 0.78 V *vs.* Fc/Fc^+^, corresponding to HOMO level of –5.58 eV, and irreversible reduction at –1.50 V, corresponding to LUMO level of –3.30 eV, and resulting HLG value of 2.28 eV which is similar to the calculated value of 2.39 eV (see Section S9 in ESI†). While the current methodology was demonstrated for the cyclization of tetramer, it is plausible that larger macrocycles can also be prepared with **BFI** building block, as the strain energies decrease after reaching a maxima for the pentamer (Fig. 1a).

**Fig. 2:**
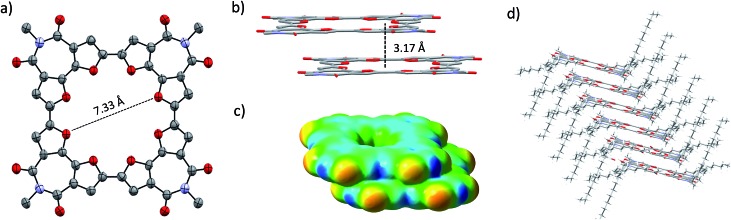
X-ray structure of **C-4BFI**. (a) Ellipsoid representation and (b) packing in stick representation excluding the 2-octyldodecyl groups. Hydrogens and solvent molecules are omitted for clarity. (c) Electrostatic potential map of **C-4BFI** dimer, calculated at the B3LYP/6-311G(d) level. (d) Stick representation of **C-4BFI** column, including 2-octyldodecyl side groups, showing slip-stack packing arrangement.

Single crystals of **C-4BFI** were grown by slow diffusion of acetone into dichloromethane solution, yielding red needles. The single crystal X-ray analysis shows that the macrocycle is planar (Fig. 2a). This contrasts calculated and experimental data for **C-nT**: the DFT predicts a spiderlike up and down conformation for **C-8T**,^27^,^33^ while the X-ray crystal structure for **C-10T** (the smallest structure available for marocyclic thiophenes) shows that the thiophene rings twist with dihedral angles of 26–34°.^23^ In order to compare the net heteroatom effect, we have calculated the thiophene analog of **C-4BFI**, **C-4BTI**. We found that while **C-4BFI** is nearly planar, with a planarization energy of 0.2 kcal mol^–1^, **C-4BTI** is distorted out of planarity, with planarization energy as large as 17 kcal mol^–1^ (Fig. S34, see ESI†). Thus, the heteroatom (sulfur or oxygen) plays a crucial role in the backbone planarity. The interring bond length between the adjacent **BFI** units in **C-4BFI** is 1.43 Å, which is shorter than that in **C-4BFI** is an indication an enhanced conjugation (see Fig. 56 ESI†). The inner diameter of **C-4BFI** macrocycles is 7.33 Å, corresponding to a van der Waals cavity of 4.3 Å. This is similar to the predicted inner cavity for 24-crown-8 ether (4–4.5 Å),^44^ yet achieved in a highly rigid π-conjugated structure that is interesting as a host for supramolecular engineering of optoelectronic materials.^34^

The macrocycles pack in a slip-stacked motif (Fig. 2b), with a very short intermolecular π–π stacking distance of 3.17 Å (closest C···C distance 3.26 Å). The short packing distances contrast sharply with those found in thiophene–acetylene macrocycles (3.7 Å)^24^ or in **C-10T** (>8 Å),^23^ and is even closer than the interplane distance of graphite (3.35 Å). This tight packing is a result of the planarity of the macrocycle and the quadrupole moment brought about by four imide groups, which induce strong π–π interactions. The calculated electrostatic potential (ESP) map of the dimer, extracted from the X-ray structure, explains the slippage as resulting from interactions between the electron-poor imide groups and the electron-rich bifuran units (Fig. 2c). The large 2-octyldodecyl groups protrude up and down on four sides of the macrocycle, forming isolated π-channels with strong but one-dimensional interactions (Fig. 2d).

**Fig. 3:**
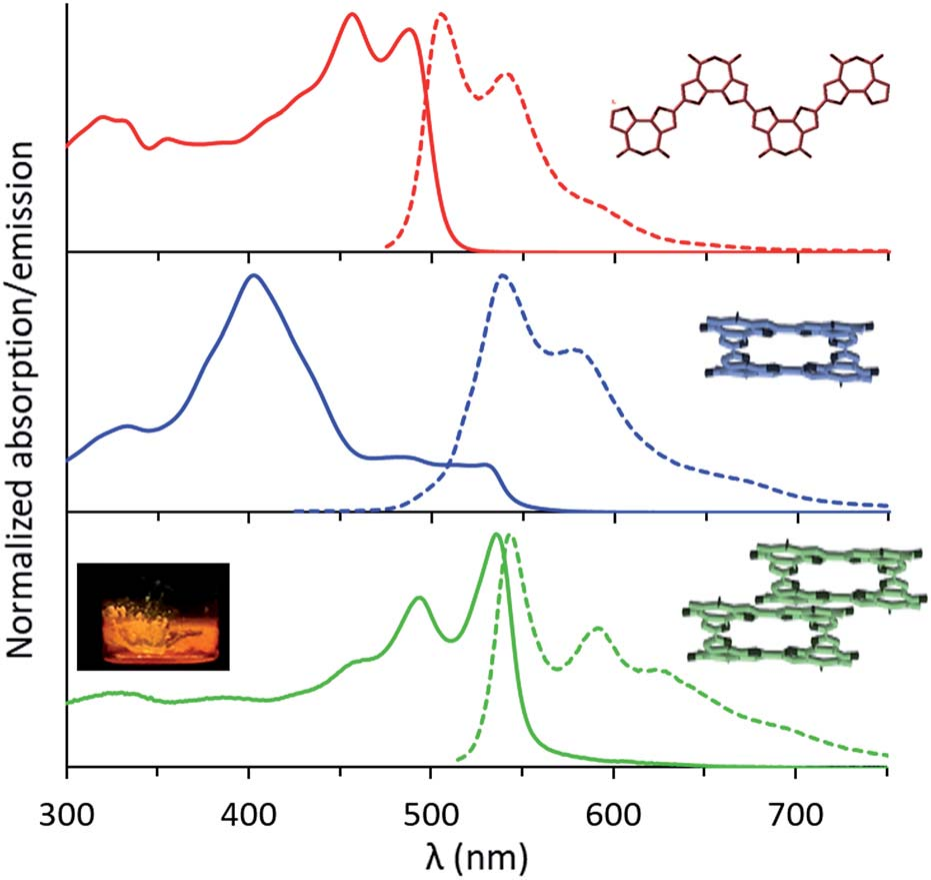
Normalized UV-Vis absorption (solid line) and emission (dashed line) spectra for **L-4BFI** (red), **C-4BFI** (blue) in chloroform, and **C-4BFI** film (green). Inset: photograph of solid **C-4BFI** fluorescing upon illumination with UV light (365 nm).

The UV-Vis absorption spectrum of the linear tetramer, **L-4BFI** in chloroform, displays the expected π–π* transition, with two vibronic peaks at 457 nm and 489 nm corresponding to a C=C backbone stretch of 0.178 eV (1432 cm^–1^; Fig. 3, red trace). The emission spectrum shows the similarly structured S^1^ → S^0^ transition, with a Stokes shift of 0.09 eV. For the macrocyclic oligofuran **C-4BFI**, the absorption maximum measured in chloroform is at 401 nm (Fig. 3, blue trace), corresponding to the S^0^ → S^2^ transition (extinction coefficient of 1.1 × 10^5^ cm^–1^ M^–1^). The shoulder that appears in the ∼480–520 nm range is likely associated with the S^0^ → S^1^ transition, which is Laporte forbidden due to a conservation of orbital symmetry in the centrosymmetric macrocycle. The emission spectrum shows a similar pattern for the macrocycle and the linear tetramer, corresponding to the S^1^ → S^0^ transition. However, the emission maximum is bathochromically shifted by 0.142 eV for **C-4BFI** compared with **L-4BFI**, which is in-line with the calculated difference in their HOMO–LUMO gap. The fluorescence quantum yield for the macrocycle measured in chloroform at 450 nm excitation is 18%, which is smaller than that of the linear oligomer (61%), in line with the symmetry-forbidden S^1^ → S^0^ transition. However, it is larger than the fluorescence quantum yield of macrocyclic oligothiophenes.^24^

**Fig. 4:**
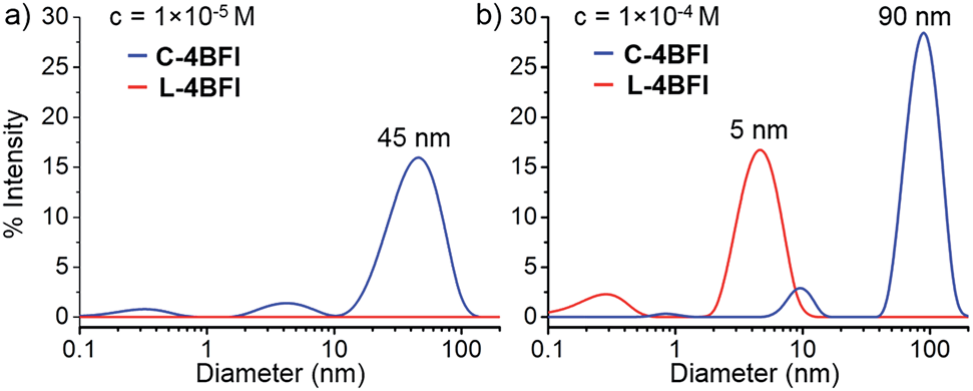
Dynamic light scattering (DLS) of **C-4BFI** and **L-4BFI** in hexane. (a) *c* = 1 × 10^–5^ M, and (b) *c* = 1 × 10^–4^ M.

The absorption spectrum of **C-4BFI** in hexane at high dilution is similar to that observed in chloroform, with a strong S^0^ → S^2^ transition and a weak S^0^ → S^1^ tail. Increasing the concentration leads to the emergence of new transitions at 535 nm and 489 nm, until a solid film is formed in which only these new transitions can be observed (Fig. 3, green trace). The emission spectrum is nearly a mirror image of the absorption, with an extremely small Stokes shift of 0.034 eV, indication of a rigid structure. Aggregation is also easily observed in hexane solutions by dynamic light scattering measurements (DLS): at a concentration of 10^–5^ M, **C-4BFI** forms aggregates with a hydrodynamic diameter ∼45 nm; these grow to large particles (∼90 nm) as the concentration increases to 10^–4^ M (Fig. 4, blue trace). With time, these aggregates form a red film on the container walls. Atomic Force Microscopy (AFM) imaging of this film shows particles up to ∼100 nm size, in line with DLS measurements, and reveals their crystalline nature (see ESI†). By comparison, the linear tetramer **L-4BFI** shows a much lower tendency to aggregate, with no observable aggregates at a concentration of 10^–5^ M and only small (5 nm) aggregates appearing at 10^–4^ M (Fig. 4, red trace). ^1^H-NMR shows the concentration dependence of the chemical shift of the β-proton up from 10^–5^ M, which supports strong intermolecular interactions. The estimated association constant for dimer formation in chloroform-*d* is 725 ± 134 M^–1^ (see Section S9 in ESI†). Samples dissolved in toluene display higher solubility compared with hexane, and no change in spectra even at 10^–4^ M concentration, supporting that π–π interactions of the macrocyclic core is the main cause for the observed aggregation.

**Fig. 5:**
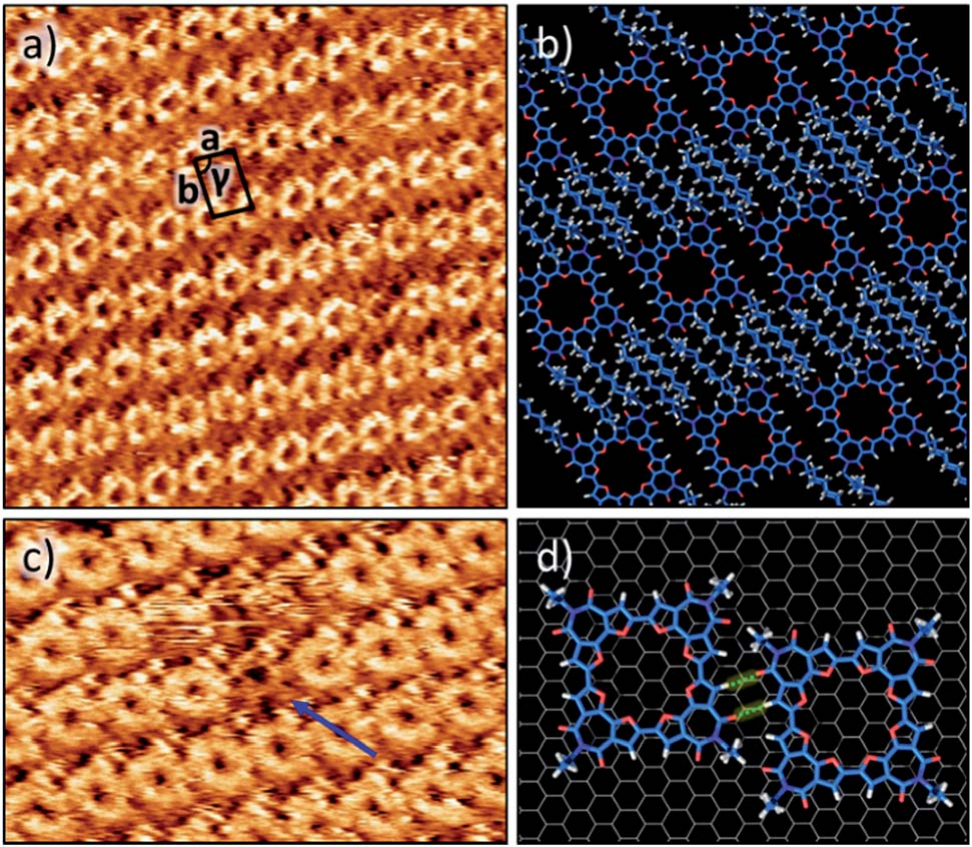
(a) STM image of **C-4BFI** (5 × 10^–2^ M) at the 1,2,4-trichlorobenzene–graphite interface. Image scale: 19.3 × 19.3 nm^2^. Imaging conditions: *I*_set_ = 1 pA, and *V*_bias_ = –1.2 V. (b) Molecular modelling unit cell parameters: *a* = 1.7 ± 0.1 nm, *b* = 2.4 ± 0.1 nm and *γ* = 84 ± 1°. (c) STM image showing the multilayer. Image scale: 13.6 × 13.6 nm^2^. Blue arrow shows the vacancy defect in the upper layer through which the STM contrast still shows a molecular structure (from the layer below). (d) Molecular model displaying C=O···H hydrogen bonding between two adjacent macrocycles.

The self-assembly of **C-4BFI** at the solid–liquid interface was imaged using STM. The macrocycles adopt a lamellar arrangement with an oblique unit cell (*a* = 1.7 ± 0.1 nm, *b* = 2.4 ± 0.1 nm, *γ* = 84 ± 1°, Fig. 5a). The conjugated macrocyclic core appears bright and the darker region between the macrocycle rows corresponds to interdigitated alkyl chains. The central cavities of the macrocycles are clearly resolved. Molecular modelling of the observed pattern suggests that, within the rows, the macrocycles are linked by two-point hydrogen bonding involving furan CH donors and carbonyl acceptors (Fig. 5b and d). Such assembly allows for interactions between only half of the alkyl chains (4 out of 8) and the HOPG surface. Presumably, the other alkyl chains are adsorbed on top of themselves (Fig. 5b) as has been observed for other alkylated molecules.^45^ We also note a pronounced tendency of the macrocycle to form multilayers at the liquid–solid interface, in line with its aggregation in solution.^46^Fig. 5c shows the multilayer, with molecules missing from the upper layer giving the appearance of holes in which a lower contrast molecule (of the lower layer) can still be identified.^47^

**Fig. 6:**
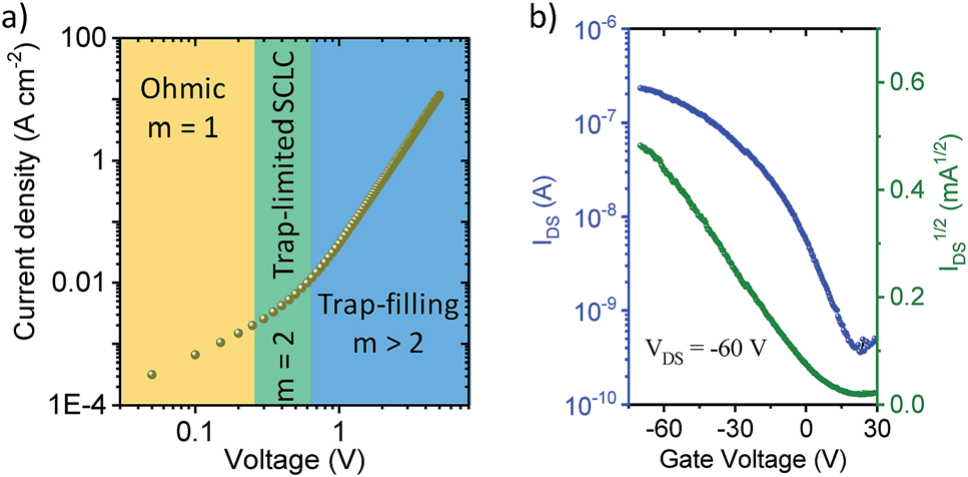
(a) Hole-only diode SCLC characteristics of **C-4BFI**, where *m* is the slope; (b) transfer characteristics of **C-4BFI** films blended with nitrofluorene acceptor.

The out-of-plane charge transport properties of **C-4BFI** films were investigated in a diode configuration. The log–log plots of current-density/voltage (*J*–*V*) of the hole-only diodes (Fig. 6a) display ohmic, trap-limited space charge limited current (SCLC) and trap filling regimes. The charge carrier mobility (μ) in SCLC regime was determined using the Mott-Gurney's law to be 2 × 10^–4^ cm^2^ V^–1^ s^–1^, which is within the typical range for organic (light-emitting, photovoltaic) diode applications. On the other hand, the field-effect transistor (FET) measurements for the same films did not display any field-effect current modulation, which at least in part can be explained by the very low HOMO of **C-4BFI** (–5.88 eV in gas phase) that leads to easy hole trapping. We were able to fabricate functional p-type FET devices by blending **C-4BFI** with a nitrofluorene acceptor (2,5,7-trinitro-4-(2,2,3,3,4,4,5,5,6,6-decafluorohexoxycarbonyl)-9-dicyanomethylenefluorene), in order to annihilate the hole traps (*i.e.* impurities and interfacial states with HOMO higher than that of bulk **C-4BFI**, Fig. 6b).^48^ Nevertheless, the measured field-effect hole mobility (5 × 10^–6^ cm^2^ V^–1^ s^–1^) for in-plane transport is almost two orders of magnitude lower than that measured by SCLC for out-of-plane transport. This difference points to the preferential face-on molecular orientation of the molecules (and therefore out-of-plane π-stacking direction), already seen in the STM experiments (Fig. 5).

## Conclusions

In summary, we have introduced **C-4BFI** as the first macrocyclic oligofuran. Its X-ray structure reveals a planar backbone, with very short interplanar distances, and a small inner cavity whose dimensions are similar to those of 24-crown-8 ether. The macrocycle shows a tendency to self-aggregate in solution, with ordered multilayers forming at the solid–liquid interface. It exhibits distinct absorption and emission spectra, and fluoresces in the solid state. **C-4BFI** displays a p-type behavior with anisotropic mobility of up to 2 × 10^–4^ cm^2^ V^–1^ s^–1^ in thin films. We are currently investigating the capabilities of oligofuran macrocycles to form inclusion complexes with its oxygen-containing cavities, as well as supramolecular aggregates with different side groups.

## Conflicts of interest

There are no conflicts to declare.

## Supplementary Material

Supplementary information

Supplementary information

Crystal structure data
