# Supplementary material for: A macrocyclic oligofuran: synthesis, solid state structure and electronic properties
Source: Chem Sci. 2019 Aug 19;10(37):8527–32. doi: 10.1039/c9sc03247a (PMC7003964; doi:10.1039/c9sc03247a)
Supplement: Supplementary file 2 [file SC-010-C9SC03247A-s002.pdf]

Electronic Supplementary Material (ESI) for Chemical Science.
This journal is © The Royal Society of Chemistry 2019

Supporting Information

A Macrocyclic Oligofuran: Synthesis, Solid State Structure and Electronic Properties

Sandip V. Mulay,[‖a,d] Or Dishi,[‖a] Yuan Fang,[‖b] Muhammad R. Niazi,[b] Linda J. W. Shimon,[c] Dmitrii F. Perepichka[b]* and Ori Gidron[a]*

[a]Institute of Chemistry, The Hebrew University of Jerusalem, Edmond J. Safra Campus, Jerusalem, Israel. [b]Department of Chemistry and Center for Self-Assembled Chemical Structures, McGill University, Montreal, QC, H3A 0B8 Canada. [c]Chemical Research Support Unit, Weizmann Institute of Science, Rehovot, Israel.

[d]Current address: Artificial Photosynthesis Research Group, Korea Research Institute of Chemical Technology (KRICT), 100 Jang-dong, Yuseong, Daejeon 305 600, Republic of Korea.

[‖]These authors contributed equally.

Table of Contents

S1. General Information 3
S2. Synthetic Scheme 4
S3. Synthetic Procedures 5
Synthesis of *N*-(2-octyldodecyl)- 5,-bromo-2,2'-bifuran-3,3'-dicarboximide (**4**) 5
Synthesis of *N*-(2-octyldodecyl)-(tributylstannyl)-2,2'-bifuran-3,3'-dicarboximide (**1**) 5
Synthesis of Bis (*N*-2-octyldodecyl-2,2'-bifuran-3,3'-dicarboximide) (**L-2BFI**) 6
Synthesis of **2** 7
Synthesis of **L-4BFI** 8
Synthesis of **3** 9
Synthesis of **C-4BFI** 10
S4. NMR Spectra 11
S5. X-Ray Data Collection and Structure Refinement 30
S6. Computational Details 32
S6.1. Absolute Energies of Neutral Structures 33
S6.2. Optimized Structures of Selected Macrocycles 34
S6.3. Frontier Molecular Orbitals for Selected Macrocycles 38
S6.4. Calculated HOMO-LUMO gaps for selected oligomers 42
S6.5. TD-DFT calculation of **C-4BFI-H** (B3LYP/6-311g(d) 43
S7. Experimental Absorption, Emission, and Excitation Spectra 45
S8. Aggregation NMR Studies 51
S9. Electrochemistry 54
S10. Bond Length Alternations 55
S11. Scanning Probe Microscopy measurements 59
S12. Dynamic Light Scattering (DLS) 61
S13. Device Fabrication 62
References 64

S1. General Information

The reagents and chemicals used here were commercially available and were used without further purification unless otherwise stated. Flash chromatography (FC) was performed using CombiFlash SiO_2 columns. The compound *N*-(2-octyldodecyl)-2,2'-bifuran-3,3'-dicarboximide (**L-1BFI**) was synthesized according to the procedures detailed in the literature.[1] ^{1}H and ^{13}C NMR spectra were recorded in solution on a Brucker-AVIII 400 MHz and 500 MHz spectrometers using tetramethylsilane (TMS) as the external standard. The spectra were recorded using chloroform-*d* as the solvent. Chemical shifts are expressed in δ units. UV-vis absorption and fluorescence spectra of synthesized compounds were recorded on an Agilent Technologies Cary 5000 UV-Vis-NIR spectrophotometer. Fluorescence measurements were carried out with a Horiba Scientific Fluoromax-4 spectrofluorometer.

High resolution mass spectra were measured on a HR Q-TOF LCMS and Waters Micromass GCT_Premier Mass Spectrometer using ESI. MALDI-TOFMS spectra were acquired using an MALDI-TOF/TOF autoflex speed mass spectrometer (Bruker Daltonilk GmbH, Bremen, Germany), which is equipped with a smartbeam-II solid-state laser (modified Nd:YAG laser) λ = 355 nm. The instrument was operated in positive ion, reflectron mode. The accelerating voltage was 21.0 kV. The delay time was 130 ns. Laser fluence were optimized for each sample. The laser was fired at a frequency of 2 kilohertz and spectra were accumulated in multiples of 500 laser shots, with 1500 shots in total. *Sample preparation*: 2-[(2E)-3-(4-tert-Butylphenyl)-2-methylprop-2-enylidene] malononitrile (DCTB) matrix solutions were made to a concentration of 20 mg/ml in dichloromethane (DCM). Sample solutions were made to an approximate concentration of 5 mg/ml in DCM. Sample and matrix solutions were premixed in ratio of 1:10 or 1:40 (v/v). A volume of 0.5 ul of this mixture was disposed on MALDI steel target plate. After evaporation of the solvent the target was inserted into mass spectrometer

S2. Synthetic Scheme

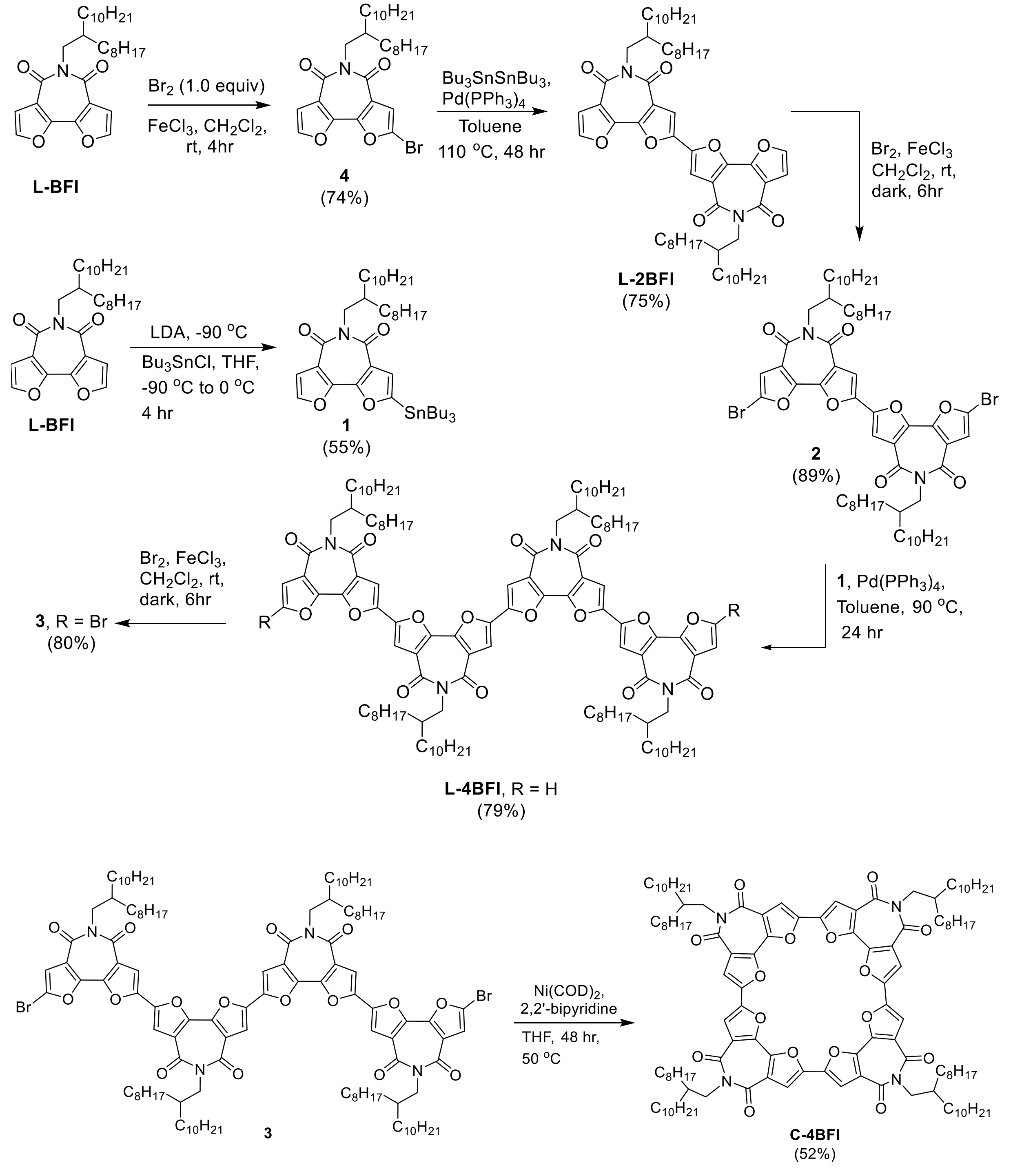

Scheme S1. Synthesis route to **C-4BFI**.

S3. Synthetic Procedures

Synthesis of *N*-(2-octyldodecyl)- 5,-bromo-2,2'-bifuran-3,3'-dicarboximide (**4**)

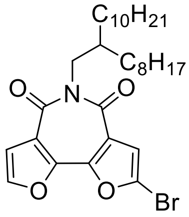

Bromine (0.21 mL, 4.13 mmol) and a catalytic amount of $FeCl_3$ (13 mg, 2 mol%) were added to a solution of *N*-(2-octyldodecyl)-2,2'-bifuran-3,3'-dicarboximide (**L-1BFI**) (2.0 g, 4.13 mmol) in CH_2Cl_2 (50 mL) and the reaction mixture was stirred in the dark for 4 h. After completion of the reaction, it was quenched using a sat. aq. solution of $Na_2S_2O_3$ and stirred for a further 30 min. The reaction mixture was poured into CH_2Cl_2 (150 mL) and washed with water (2 × 100 mL), brine, dried (Na_2SO_4), filtered, and concentrated. The residue obtained was purified by flash column chromatography on silica gel using hexane and CH_2Cl_2 (1:1) as eluent to give **4** as a light yellow solid (1.71 g, 74% yield). **^{1}H NMR** (500 MHz, Chloroform-*d*) δ 7.53 (d, *J* = 1.9 Hz, 1H), 7.13 (d, *J* = 1.9 Hz, 1H), 7.04 (s, 1H), 4.19 (d, *J* = 7.2 Hz, 2H), 1.87-1.82 (m, 1H), 1.35 – 1.16 (m, 32H), 0.85 (t, *J* = 7.0 Hz, 3H), 0.84 (t, *J* = 7.0 Hz, 3H); **^{13}C NMR** (126 MHz, Chloroform-*d*) δ 160.38, 159.67, 144.43, 143.85, 142.29, 125.58, 121.87, 120.40, 115.71, 114.41, 48.58, 36.35, 32.04, 32.01, 31.78, 30.16, 29.77, 29.75, 29.71, 29.66, 29.46, 29.42, 26.58, 22.80, 22.78, 14.22; **HRMS** (ESI) calcd. for $C_{30}H_{45}BrNO_4$ 564.2511, found 564.2503 $(M + H)^+$.

Synthesis of *N*-(2-octyldodecyl)-(tributylstannyl)-2,2'-bifuran-3,3'-dicarboximide (**1**)

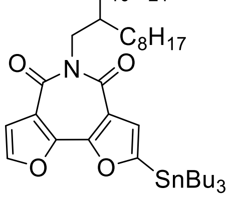

A solution of lithium diisopropylamide (LDA; 2.3 mL, 2.0 M in hexane, 4.54 mmol) was added dropwise to a solution of **L-1BFI** (2.0 g, 4.13 mmol) in dry THF (80 mL) at -90 °C under N_2 and

stirred for 1 hr. Bu_3SnCl (1.68 mL, 6.19 mmol) was added dropwise, and the reaction mixture was allowed to reach at 0 °C and stirred for 2 hr. The mixture was quenched with water, extracted with CH_2Cl_2 (2 × 80 mL), dried (Na_2SO_4), filtered, and evaporated. The residue obtained was purified by flash column chromatography on silica gel basified with trimethylamine using hexane as eluent to give **1** as a colorless oil (1.75 g, 55% yield). **^{1}H NMR** (500 MHz, Chloroform-*d*) δ 7.53 (d, *J* = 1.9 Hz, 1H), 7.26 (d, *J* = 2.4 Hz, 1H), 7.15 (d, *J* = 1.9 Hz, 1H), 4.25 (d, *J* = 7.3 Hz, 2H), 1.89 (p, *J* = 5.6, 5.1 Hz, 1H), 1.64 – 1.55 (m, 6H), 1.45 – 1.12 (m, 44H), 0.94 – 0.81 (m, 15H); **^{13}C NMR** (126 MHz, Chloroform-*d*) δ 165.69, 161.34, 161.10, 147.52, 144.02, 143.20, 125.35, 120.66, 119.35, 114.24, 48.52, 36.54, 32.07, 32.05, 31.92, 31.90, 30.25, 30.23, 29.80, 29.78, 29.77, 29.72, 29.49, 29.46, 29.00, 27.29, 26.71, 22.84, 22.82, 14.26, 13.77, 10.61; **HRMS** (ESI) calcd for $C_{42}H_{72}NO_4Sn$ 774.4487, found 774.4512 $(M + H)^+$.

Synthesis of Bis (*N*-2-octyldodecyl-2,2'-bifuran-3,3'-dicarboximide) (**L-2BFI**)

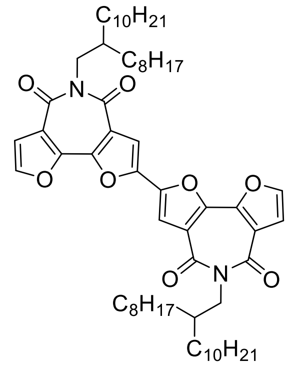

To a two-neck flask equipped with a condenser was added **4** (1.88 g, 3.34 mmol), hexabutyldistannane (969 mg, 1.67 mmol), tetrakis(triphenylphosphine)palladium(0) [$Pd(PPh_3)_4$] (192 mg, 0.167 mmol), and 70 mL dry toluene. The reaction mixture was refluxed under argon for 48 h. After cooling to room temperature, the solvent was evaporated under reduced pressure and the residue obtained was purified by flash column chromatography on silica gel using hexane and CH_2Cl_2 (1:3) as the eluent to give **L-2BFI** as a yellow solid (1.2 g, 75% yield). **^{1}H NMR** (500 MHz, Chloroform-d) δ 7.60 (d, *J* = 1.9 Hz, 2H), 7.48 (s, 2H), 7.18 (d, *J* = 1.9 Hz, 2H), 4.24 (d, *J* = 7.2 Hz, 4H), 1.88 (p, *J* = 6.1 Hz, 2H), 1.38 – 1.16 (m, 64H), 0.85 (t, *J* = 7.0 Hz, 6H), 0.84 (t, *J* = 7.0 Hz, 6H); **^{13}C NMR** (126 MHz, Chloroform-d) δ 160.42, 160.19, 144.79, 144.19, 143.19, 142.53, 121.78, 121.38, 114.69, 111.59, 48.63, 36.43, 32.05, 32.04, 31.84, 30.20, 29.80, 29.78,

29.75, 29.70, 29.48, 29.45, 26.63, 22.81, 14.24. **HRMS** (MALDI) calcd for $C_{60}H_{88}N_2O_8$ 964.654, found 964.668 $(M)^+$.

Synthesis of **2**

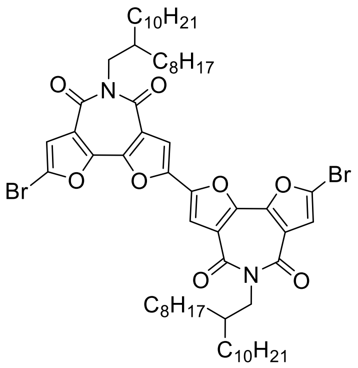

Bromine (0.11 mL, 1.98 mmol) and a catalytic amount of $FeCl_3$ (2 mg, 2 mol%) were added to a solution of imide **L-2BFI** (0.64 g, 0.66 mmol) in dry CH_2Cl_2 (40 mL) and the reaction mixture was stirred in the dark for 6 hr. After completion of the reaction, it was quenched using a sat. aq. solution of $Na_2S_2O_3$ and stirred for a further 30 min. The reaction mixture was poured into CH_2Cl_2 (60 mL) and washed with water (2 × 40 mL), brine, dried (Na_2SO_4), filtered, and concentrated. The residue obtained was purified by flash column chromatography on silica gel using hexane and CH_2Cl_2 (1:1) as eluent to give **2** as a light yellow solid (0.66 g, 89% yield). **^{1}H NMR** (500 MHz, Chloroform-*d*) δ 7.50 (s, 2H), 7.10 (s, 2H), 4.23 (d, *J* = 7.2 Hz, 4H), 1.89 -1.84 (m, 2H), 1.38 – 1.18 (m, 64H), 0.86 (t, *J* = 7.0 Hz, 6H), 0.85 (t, *J* = 7.0 Hz, 6H); **^{13}C NMR** (126 MHz, Chloroform-*d*) δ 159.82, 159.39, 144.89, 143.58, 142.16, 126.53, 122.89, 121.80, 116.14, 111.92, 48.74, 36.39, 32.07, 32.06, 31.81, 30.19, 29.81, 29.80, 29.76, 29.71, 29.50, 29.47, 26.60, 22.83, 14.26. **HRMS** (MALDI) calcd for $C_{60}H_{86}Br_2N_2O_8$ 1122.473, found 1122.505 $(M)^+$.

Synthesis of **L-4BFI**

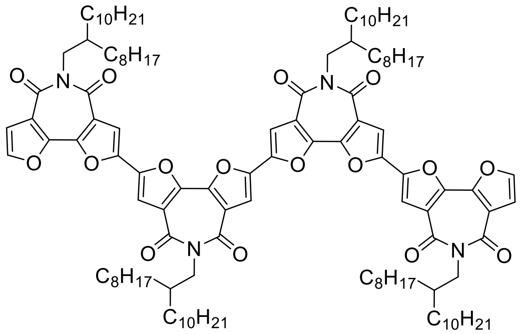

Tetrakis(triphenylphosphine)palladium(0) [(Pd(PPh_3)$_4$] 25 mg, 0.021 mmol) was added to a solution of **2** (0.27 g, 0.42 mmol) and *N*-2-octyldodecyl-5-(tributylstannyl)-2,2'-bifuran-3,3'-dicarboximide (**1**) (0.75 g, 0.97 mmol) in dry and degassed toluene (15 mL), and the reaction mixture was stirred at 90 °C for 24 h under nitrogen. Then reaction mixture was cooled to room temperature and the solvent was evaporated under reduced pressure. The residue obtained was purified by flash column chromatography on silica gel using hexane and dichloromethane (1:1 to 9:1) as eluent to give **L-4BFI** as a red solid (0.48 g, 79% yield). **^{1}H NMR** (500 MHz, Chloroform-d) δ 7.67 (d, *J* = 1.9 Hz, 2H), 7.60 (s, 2H), 7.54 (s, 2H), 7.48 (s, 2H), 7.19 (d, *J* = 1.8 Hz, 2H), 4.26 (d, *J* = 7.2 Hz, 4H), 4.24 (d, *J* = 7.2 Hz, 4H),1.90 (h, *J* = 6.3 Hz, 4H), 1.44 – 1.16 (m, 128H), 0.92 – 0.79 (m, 24H); **^{13}C NMR** (126 MHz, Chloroform-d) δ 160.29, 159.92, 159.68, 159.62, 145.29, 145.13, 144.40, 144.31, 143.25, 142.35, 142.28, 142.00, 122.85, 122.71, 121.79, 121.55, 114.69, 112.27, 112.20, 111.82, 48.77, 48.63, 36.48, 32.07, 31.90, 31.88, 30.24, 29.85, 29.83, 29.81, 29.80, 29.78, 29.75, 29.73, 29.50, 29.48, 26.67, 22.82, 14.25; **HRMS** (MALDI) calcd for $C_{120}H_{174}N_4O_{16}$ 1927.292, found 1927.291 $(M)^+$.

Synthesis of **3**

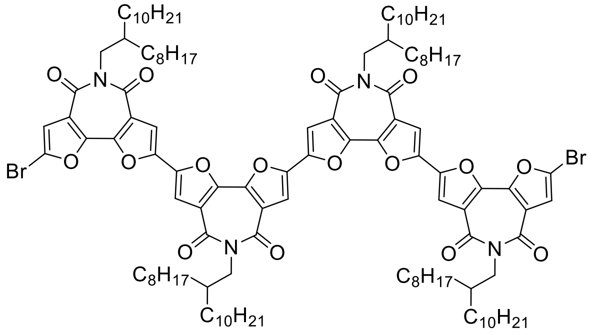

Bromine (0.041 mL, 0.794 mmol) and a catalytic amount of $FeCl_3$ (0.86 mg, 2 mol%) were added to a solution of imide **L-4BFI** (0.51 g, 0.265 mmol) in CH_2Cl_2 (50 mL) and the reaction mixture was stirred in the dark for 6 h. After completion of the reaction, it was quenched using a saturated solution of $Na_2S_2O_3$ and stirred for additional 30 min. The reaction mixture was then poured into CH_2Cl_2 (60 mL) and washed with water (2 × 40 mL), brine, dried (Na_2SO_4), filtered, and concentrated. The residue obtained was purified by flash column chromatography on silica gel using hexane and CH_2Cl_2 (1:1) as eluent to give **3** as a light orange solid (0.442 g, 80% yield). **^{1}H NMR** (500 MHz, Chloroform-*d*) δ 7.59 (s, 2H), 7.53 (s, 2H), 7.49 (s, 2H), 7.08 (s, 2H), 4.26 (d, *J* = 7.3 Hz, 4H), 4.21 (d, *J* = 7.3 Hz, 4H), 1.92 – 1.83 (m, 4H), 1.38 – 1.21 (m, 128H), 0.87 – 0.83 (m, 24H); **^{13}C NMR** (126 MHz, Chloroform-*d*) δ 159.74, 159.69 (2C), 159.35, 145.21, 145.18, 144.77, 143.53, 142.34, 142.25, 142.20, 126.61, 122.95, 122.88, 122.86, 121.86, 116.17, 112.41, 112.24, 112.22, 48.77, 36.48, 36.43, 32.08, 31.89, 31.85, 30.22, 29.84, 29.81, 29.80, 29.78, 29.75, 29.73, 29.53, 29.51, 29.49, 26.64, 22.83, 14.26; **HRMS** (MALDI) calcd for $C_{120}H_{172}Br_2N_4O_{16}$ 2085.114, found 2085.132 $(M)^+$.

Synthesis of **C-4BFI**

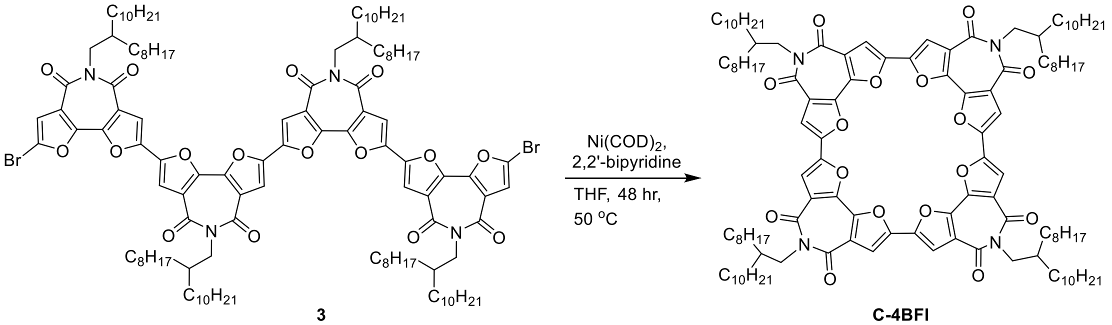

A solution of Bis(1,5-cyclooctadiene)nickel(0) [Ni(COD)$_2$] (37 mg, 0.13 mmol) and 2,2'-dipyridyl (21 mg, 0.13 mmol) in dry THF (15 mL) was stirred for 15 min at 50 °C under argon atmosphere. After cooling to room temperature, the violet solution was transferred to a solution of compound **3** (250 mg, 0.12 mmol) in THF (235 mL). The reaction mixture was stirred for 48 hr at 50 °C under argon atmosphere. The solvent was evaporated under vacuum and the crude product was purified by flash column chromatography on silica gel using hexane and CH_2Cl_2 (1:1) as eluent to give **C-4BFI** as a red solid (120 mg, 52% yield). **^{1}H NMR** (500 MHz, Chloroform-*d*) δ 7.30 (s, 8H), 4.19 (d, *J* = 7.2 Hz, 16H), 1.86 (p, *J* = 5.9 Hz, 8H), 1.38 – 0.99 (m, 128H), 0.87 (t, *J* = 7.0 Hz, 24H); **^{13}C NMR** (126 MHz, Chloroform-*d*) δ 159.47, 144.98, 142.54, 123.28, 113.56, 48.86, 36.48, 32.09, 32.07, 31.83, 30.25, 29.85, 29.83, 29.81, 29.76, 29.53, 29.50, 26.63, 22.85, 22.84, 14.27; **HRMS** (MALDI) calcd for $C_{120}H_{172}N_4O_{16}$ 1925.276, found 1925.267 $(M)^+$.

* We note that the ^{1}H NMR signals observed for **C-4BFI** are concentration-dependent due to self-association. See section S8 for details.

S4. NMR Spectra

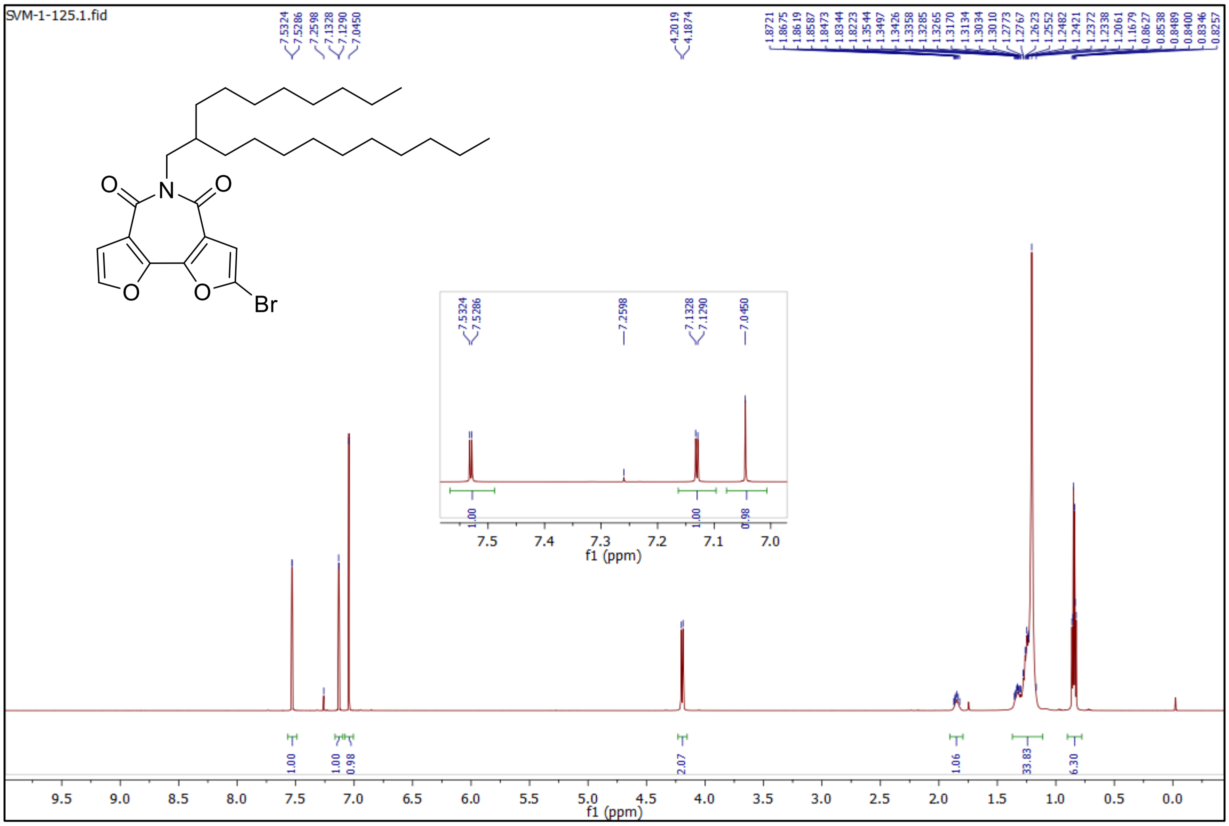

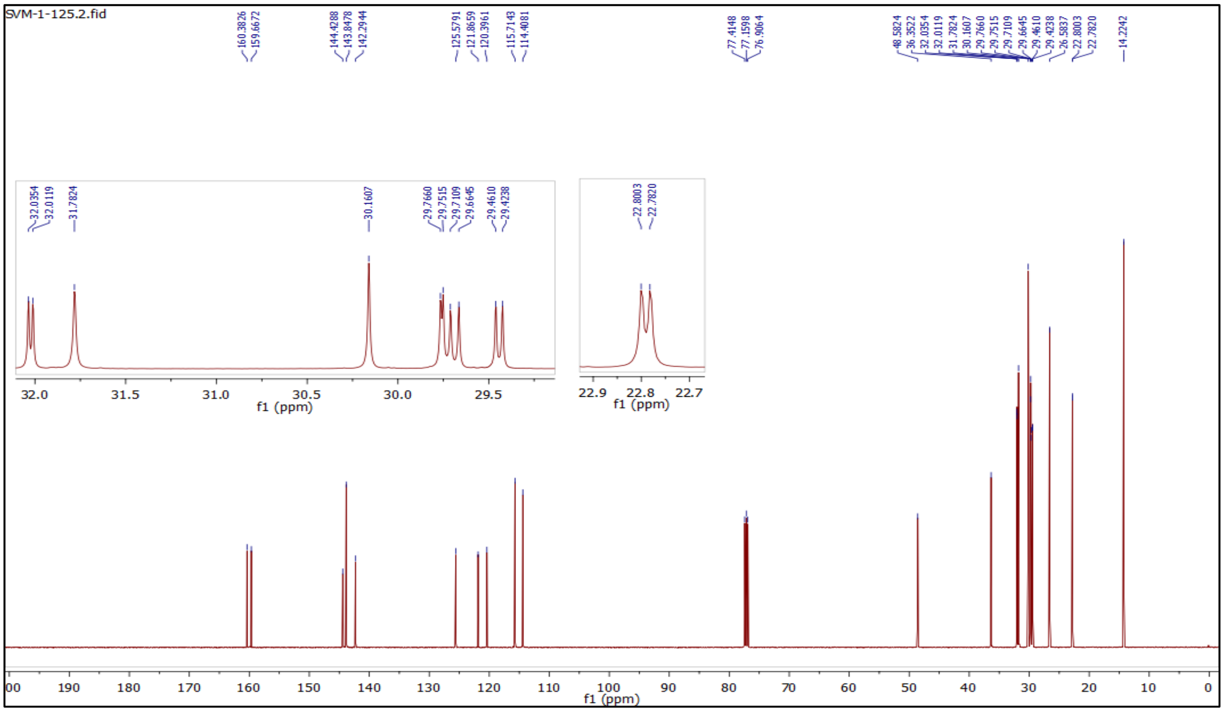

Figure S1. ^{1}H and ^{13}C NMR spectra of **4** in $CDCl_3$, measured at 298 K.

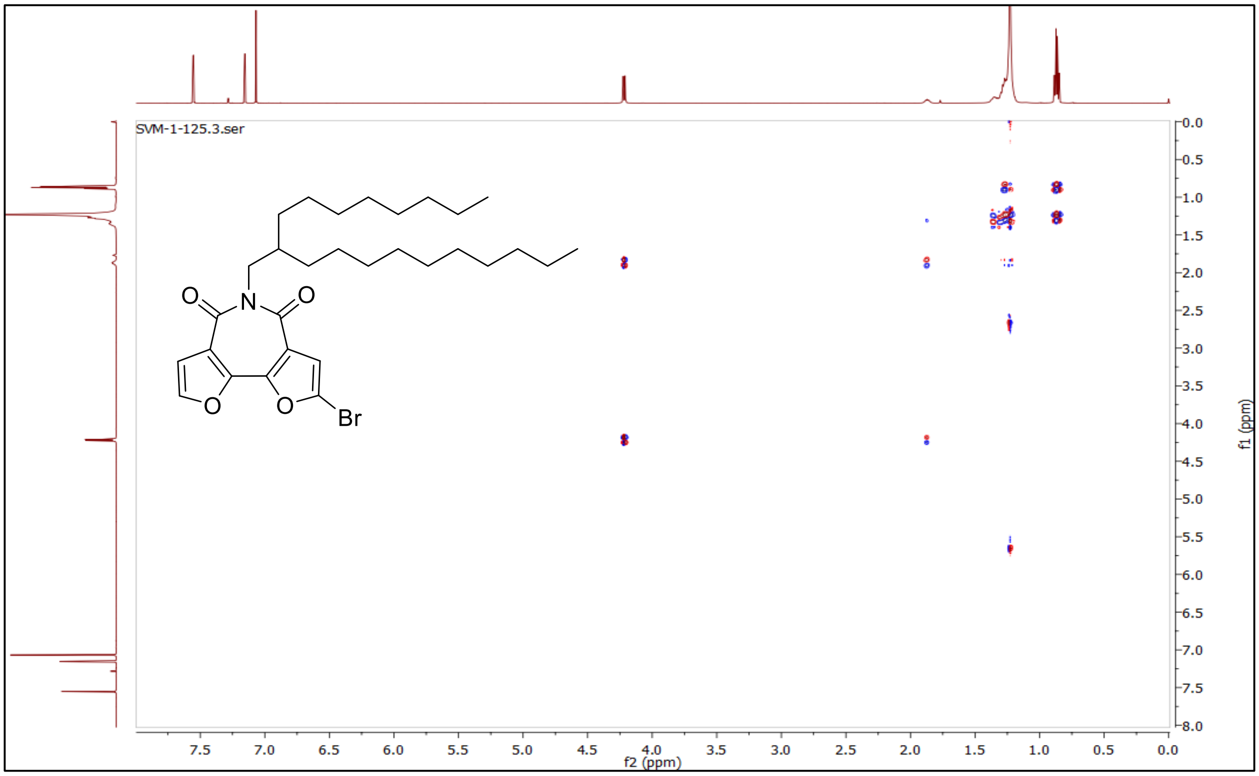

Figure S2. COSY NMR spectra of **4** in $CDCl_3$, measured at 298 K

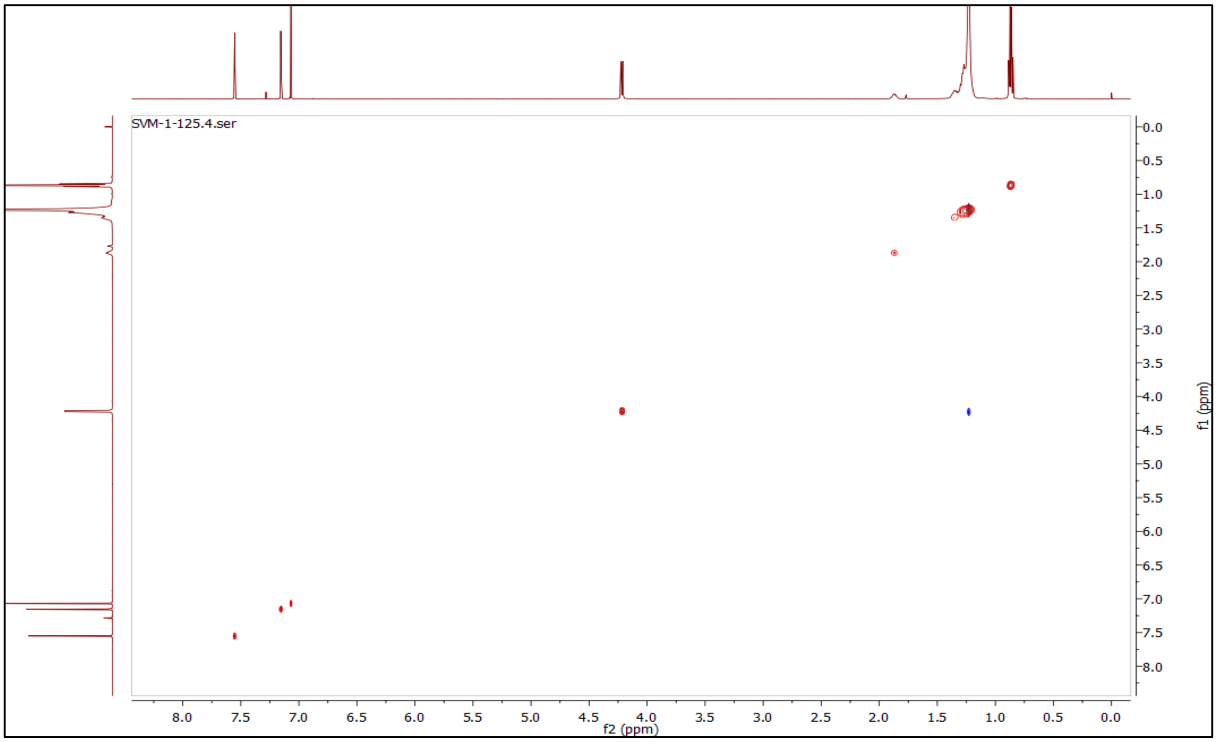

Figure S3. NOESY NMR spectra of **4** in $CDCl_3$, measured at 298 K.

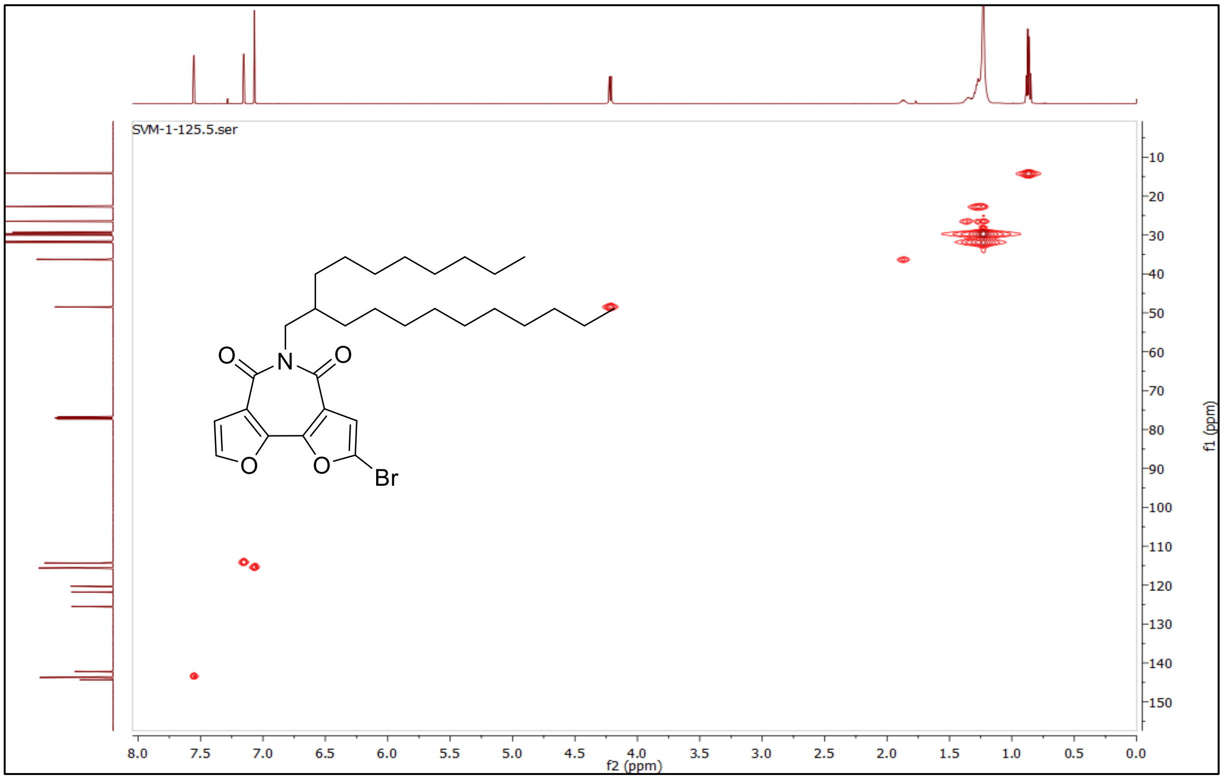

Figure S4. HSQC NMR spectra of **4** in $CDCl_3$, measured at 298 K.

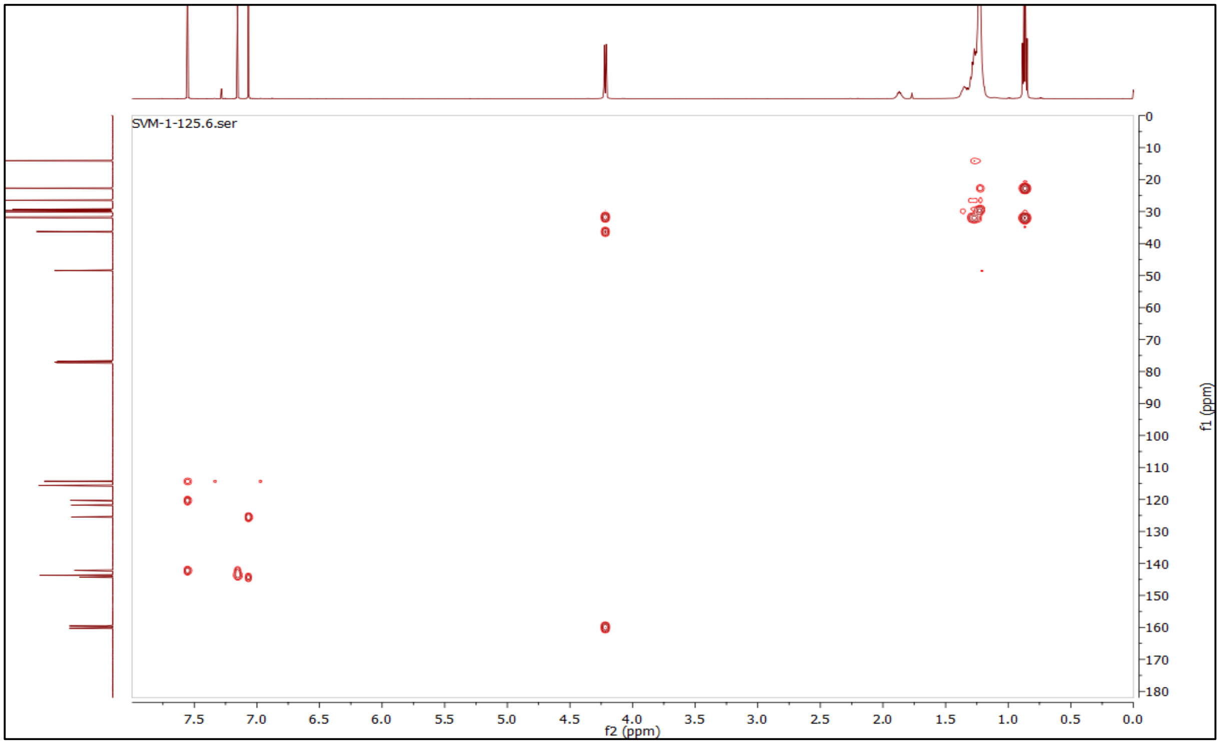

Figure S5. HMBC NMR spectra of **4** in $CDCl_3$, measured at 298 K.

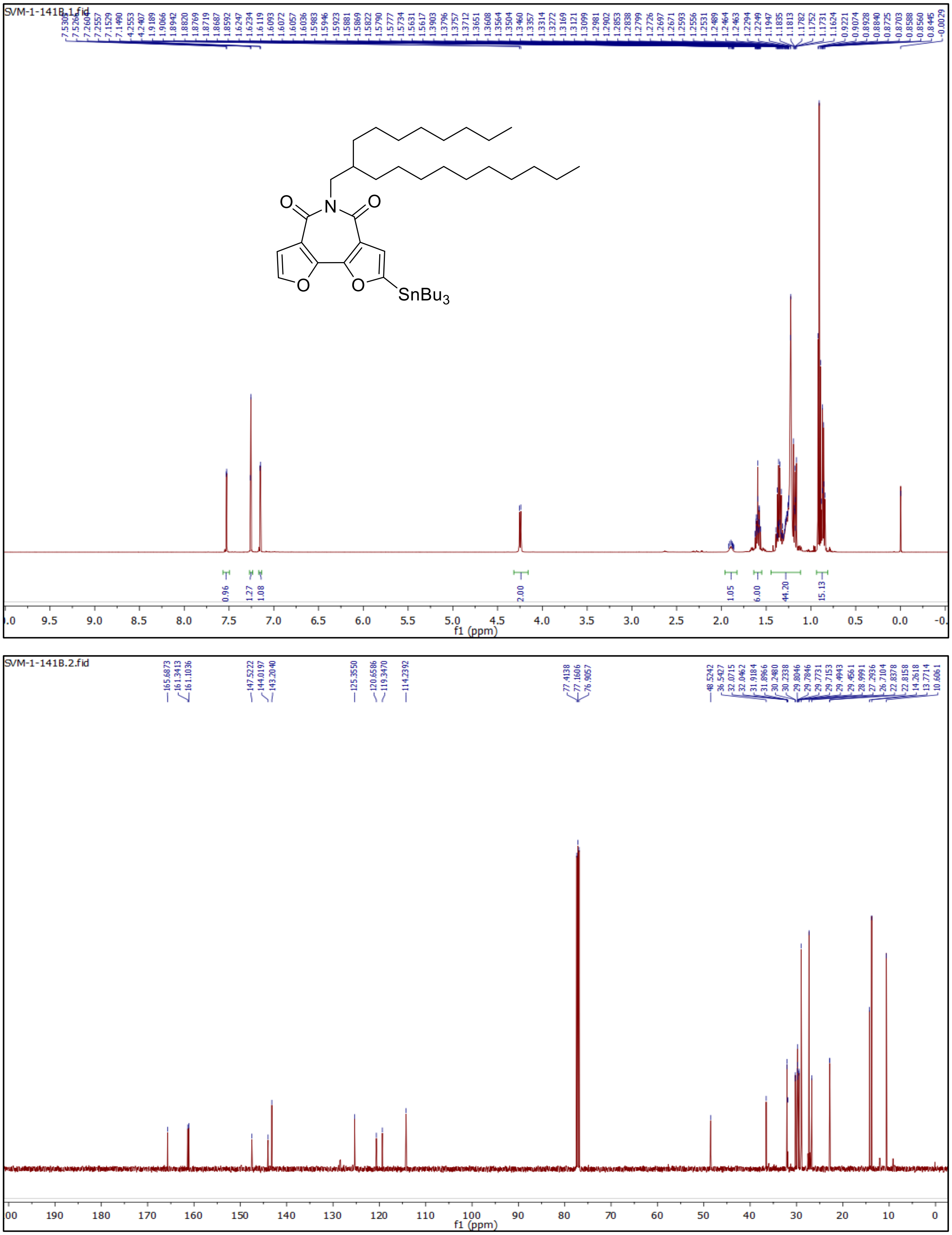

Figure S6. ^{1}H and ^{13}C NMR spectra of **1** in $CDCl_3$, measured at 298 K.

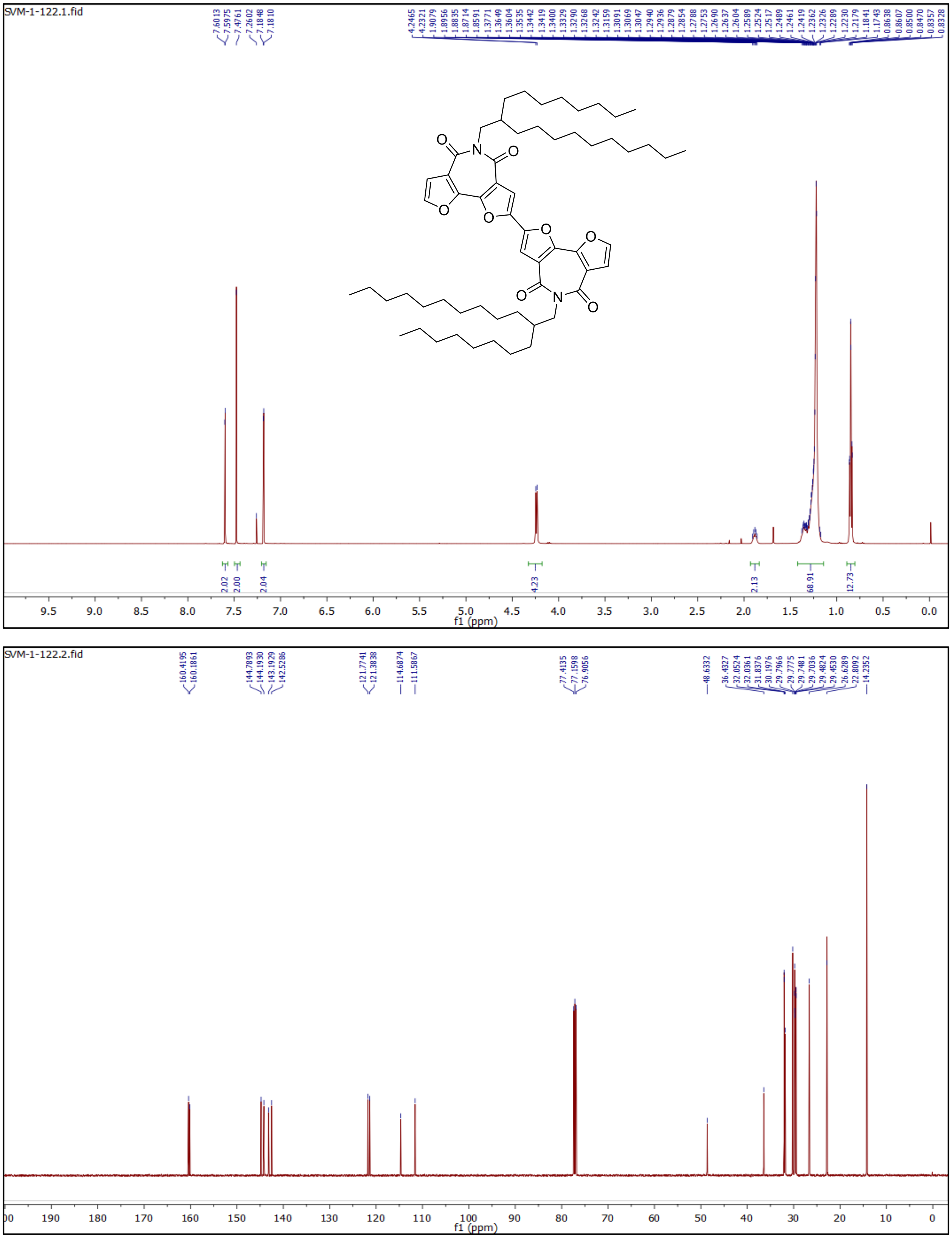

Figure S7. ^{1}H and ^{13}C NMR spectra of **L-2BFI** in $CDCl_3$, measured at 298 K.

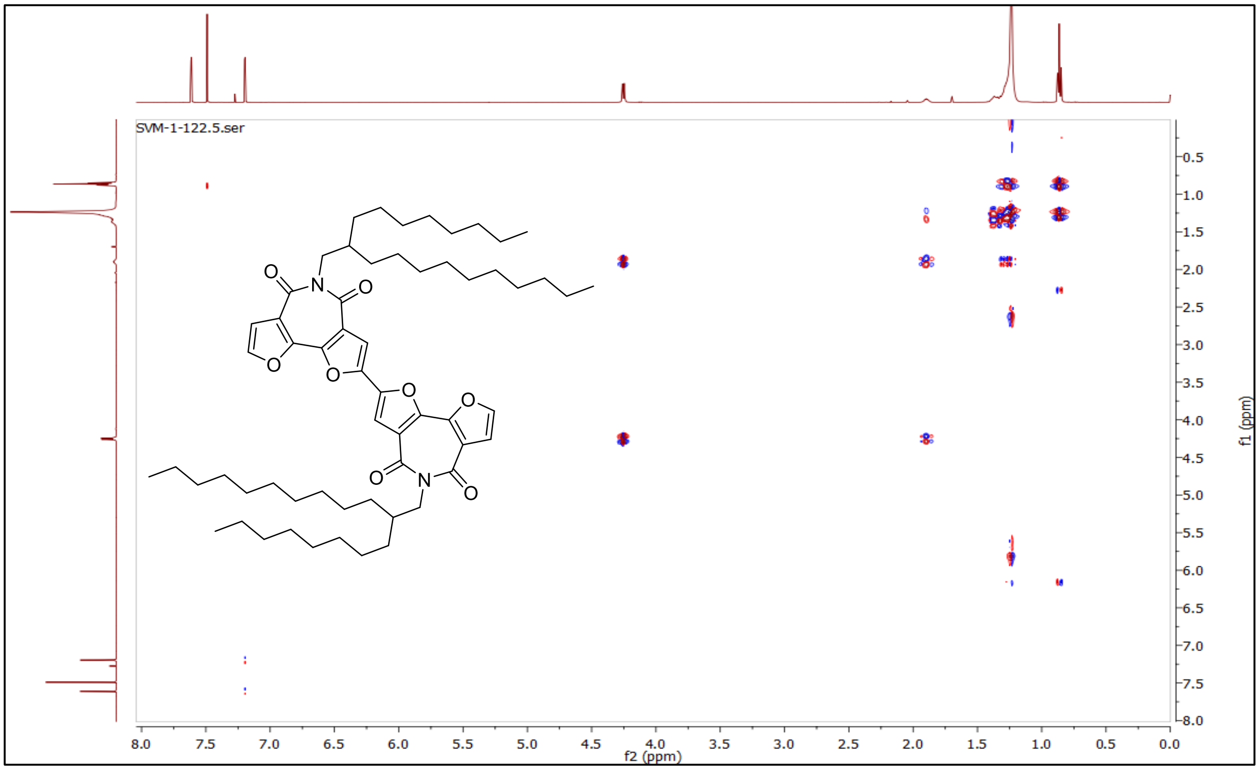

Figure S8. COSY NMR spectra of **L-2BFI** in $CDCl_3$, measured at 298 K.

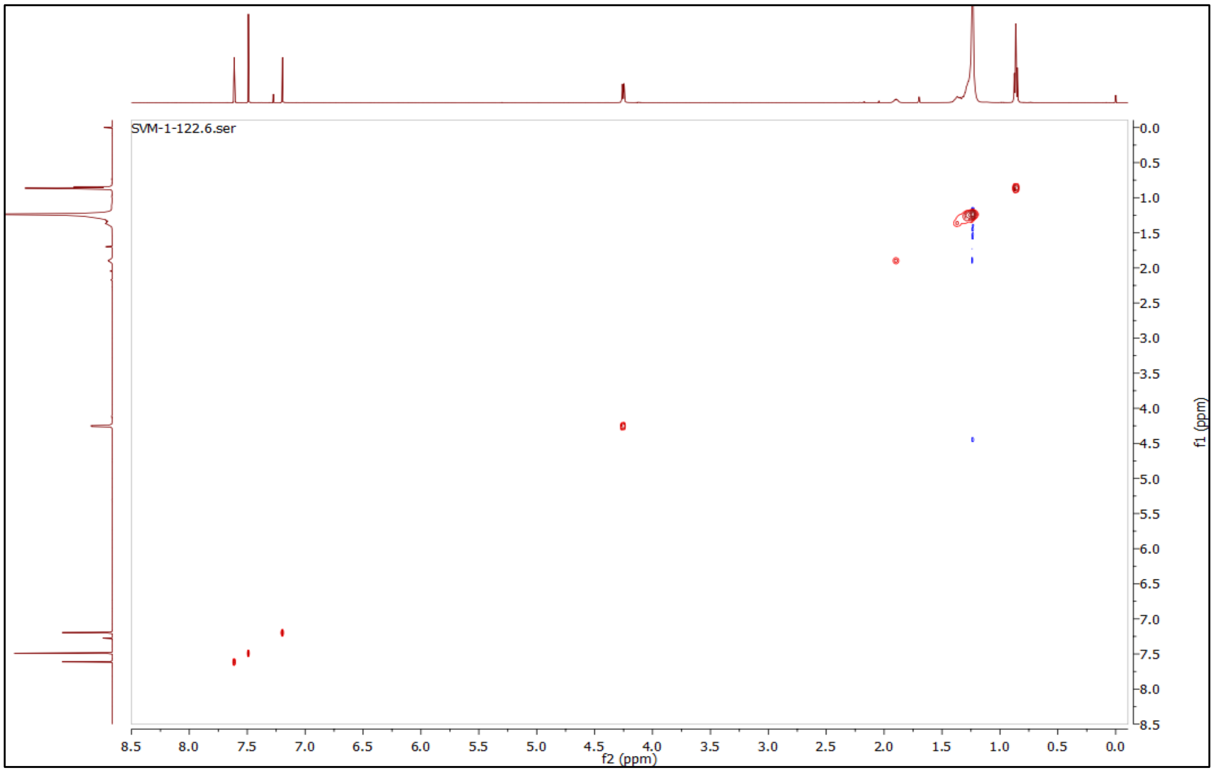

Figure S9. NOESY NMR spectra of **L-2BFI** in $CDCl_3$, measured at 298 K.

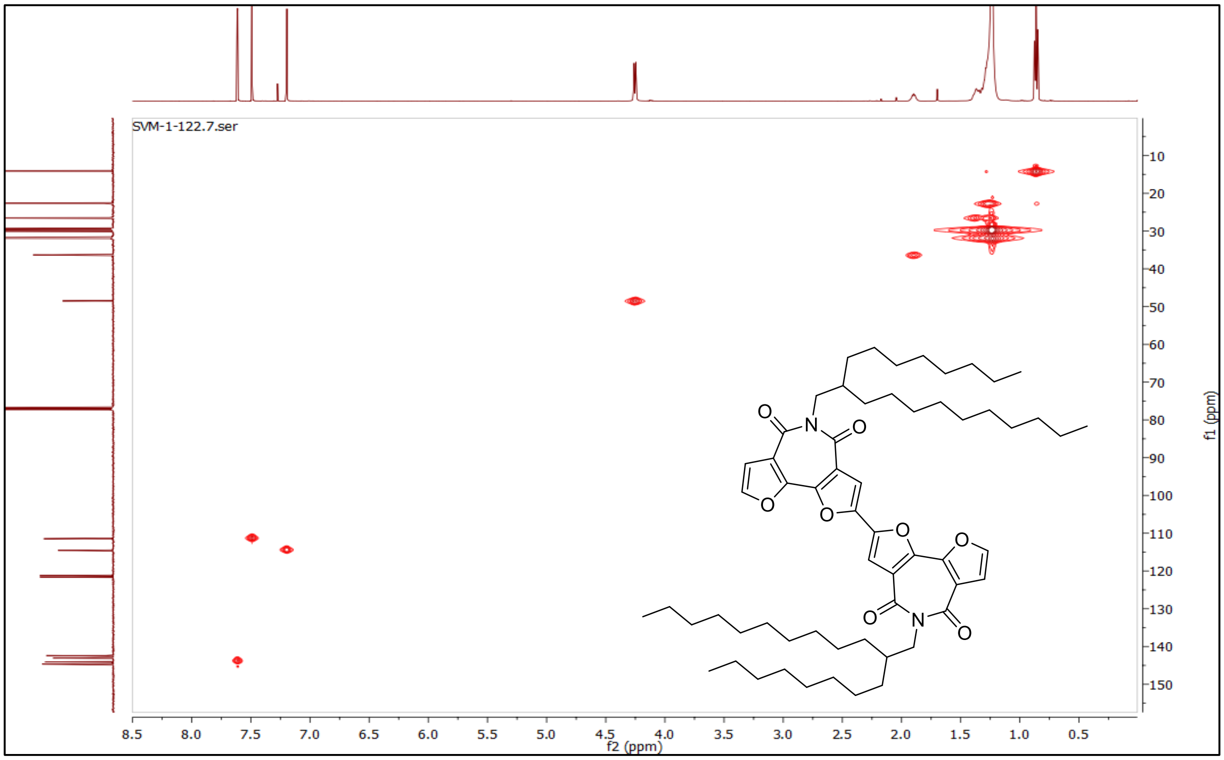

Figure S10. HSQC NMR spectra of **L-2BFI** in $CDCl_3$, measured at 298 K.

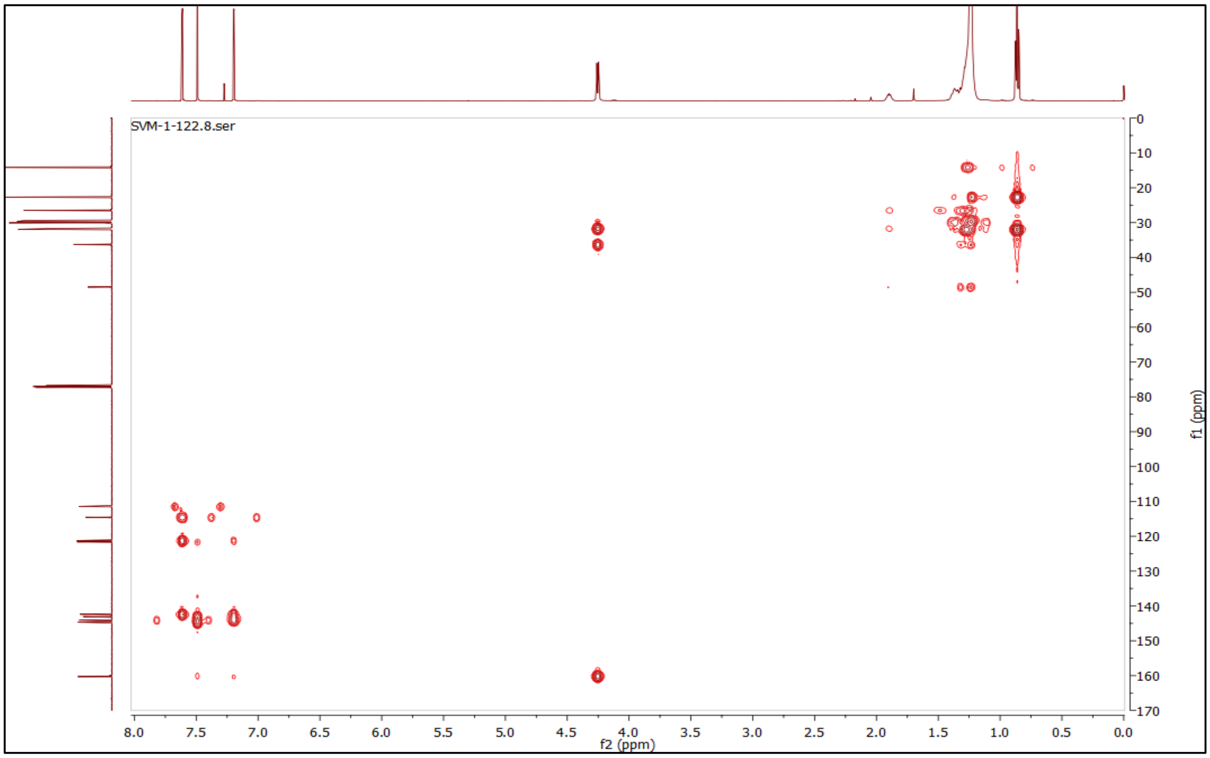

Figure S11. HMBC NMR spectra of **L-2BFI** in $CDCl_3$, measured at 298 K.

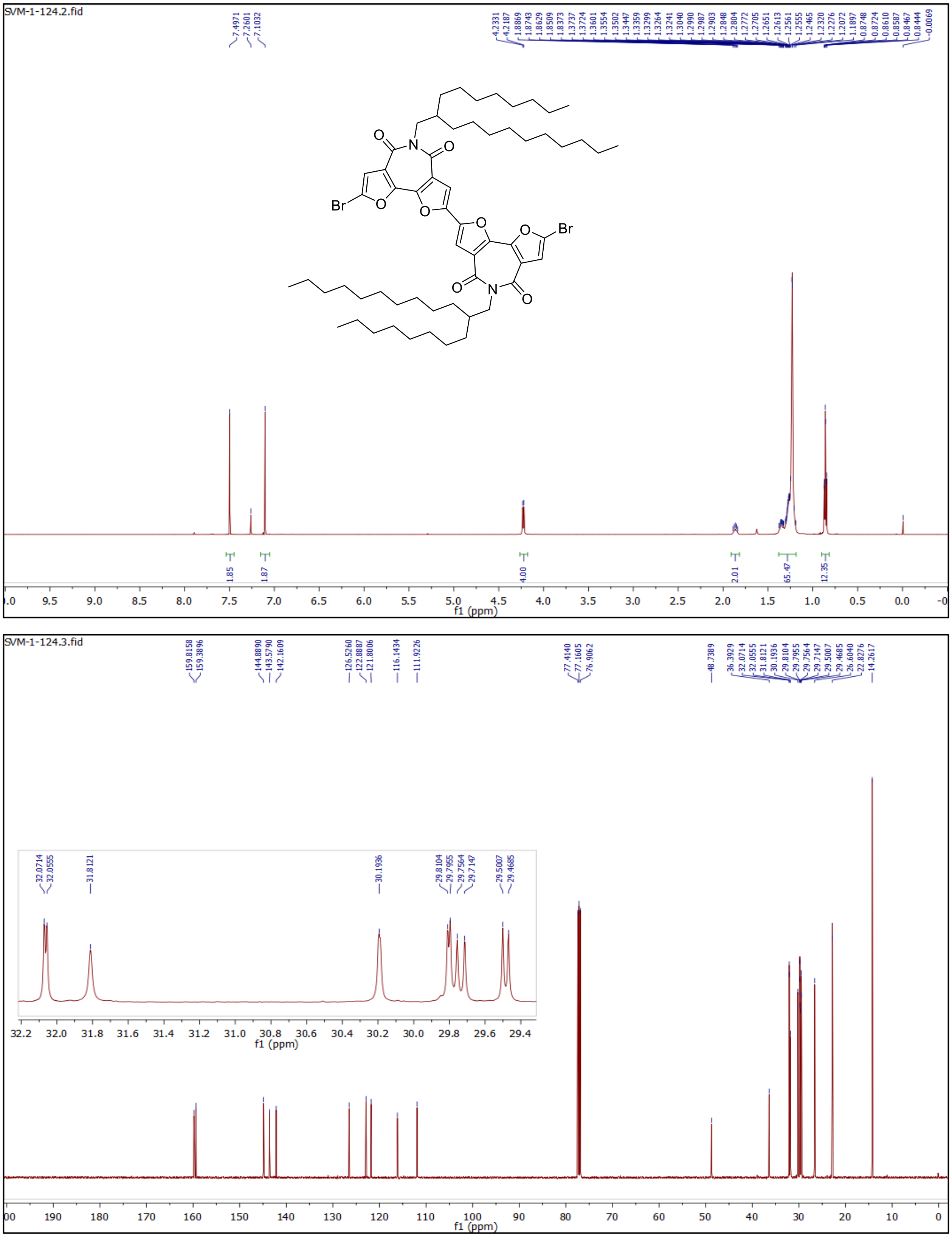

Figure S12. ^{1}H and ^{13}C NMR spectra of **2** in $CDCl_3$, measured at 298 K.

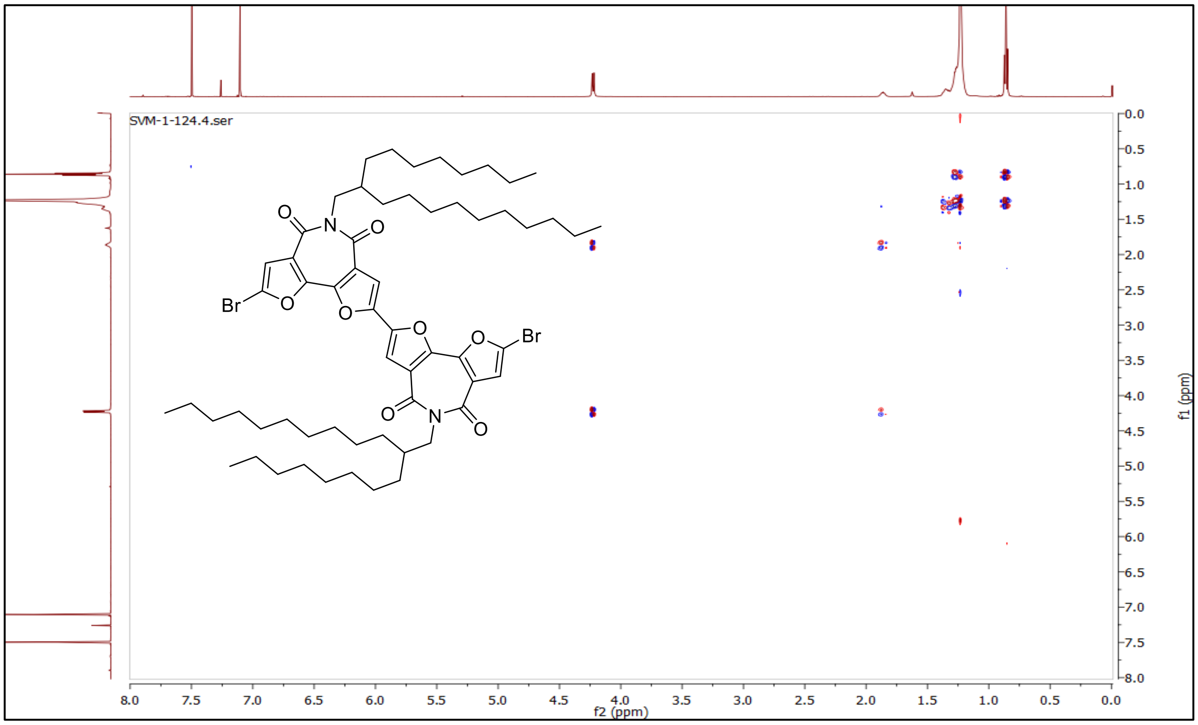

Figure S13. COSY NMR spectra of **2** in $CDCl_3$, measured at 298 K.

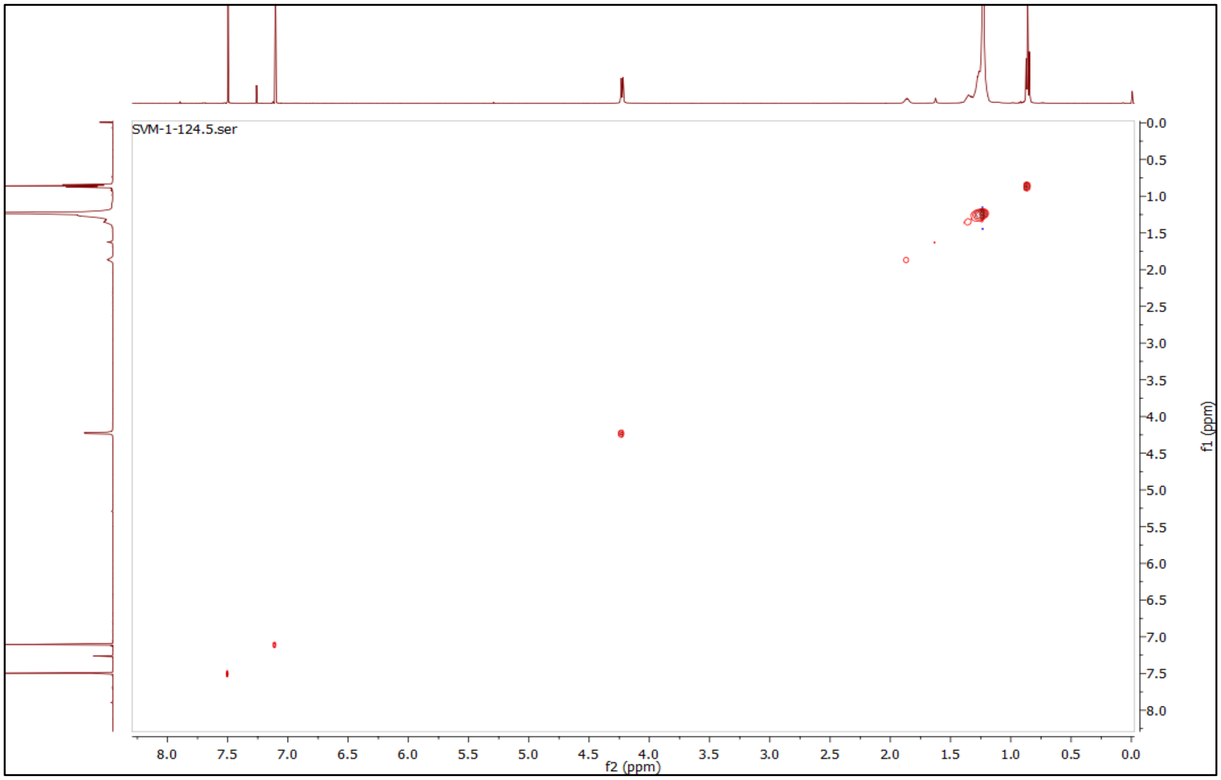

Figure S14. NOESY NMR spectra of **2** in $CDCl_3$, measured at 298 K.

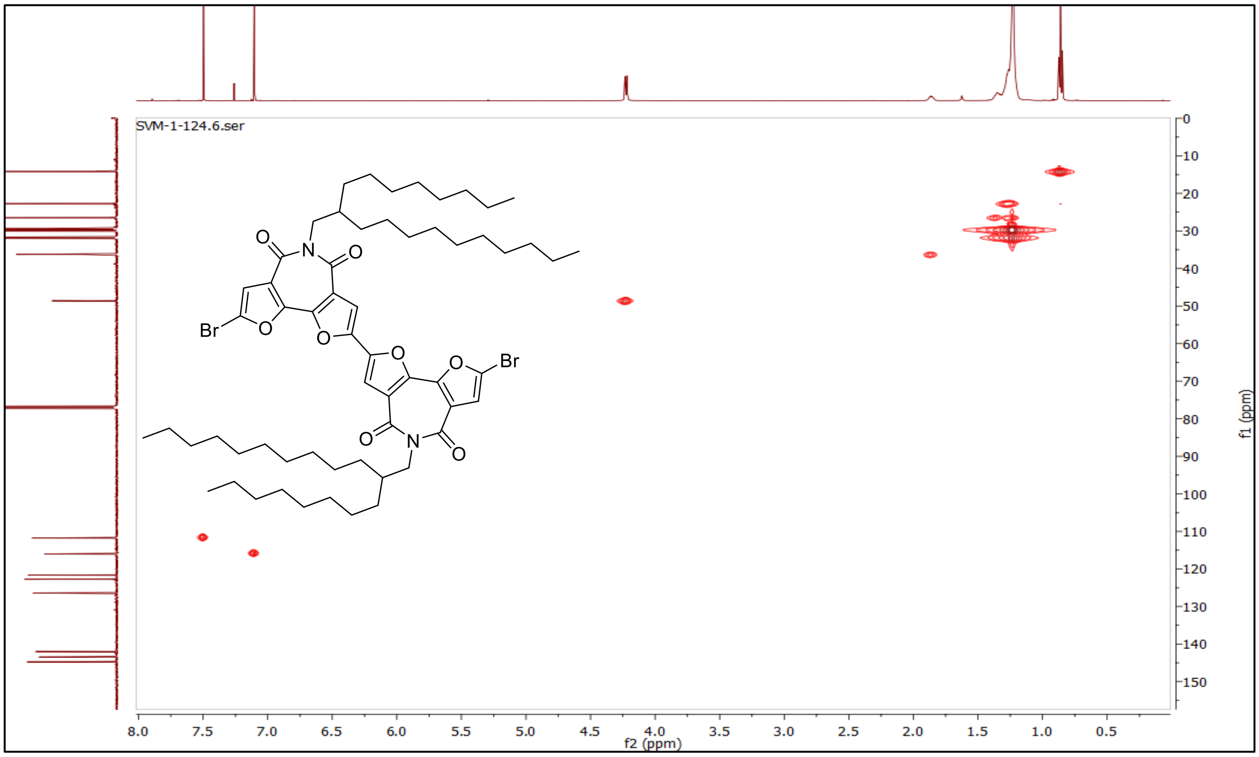

Figure S15. HSQC NMR spectra of **2** in $CDCl_3$, measured at 298 K.

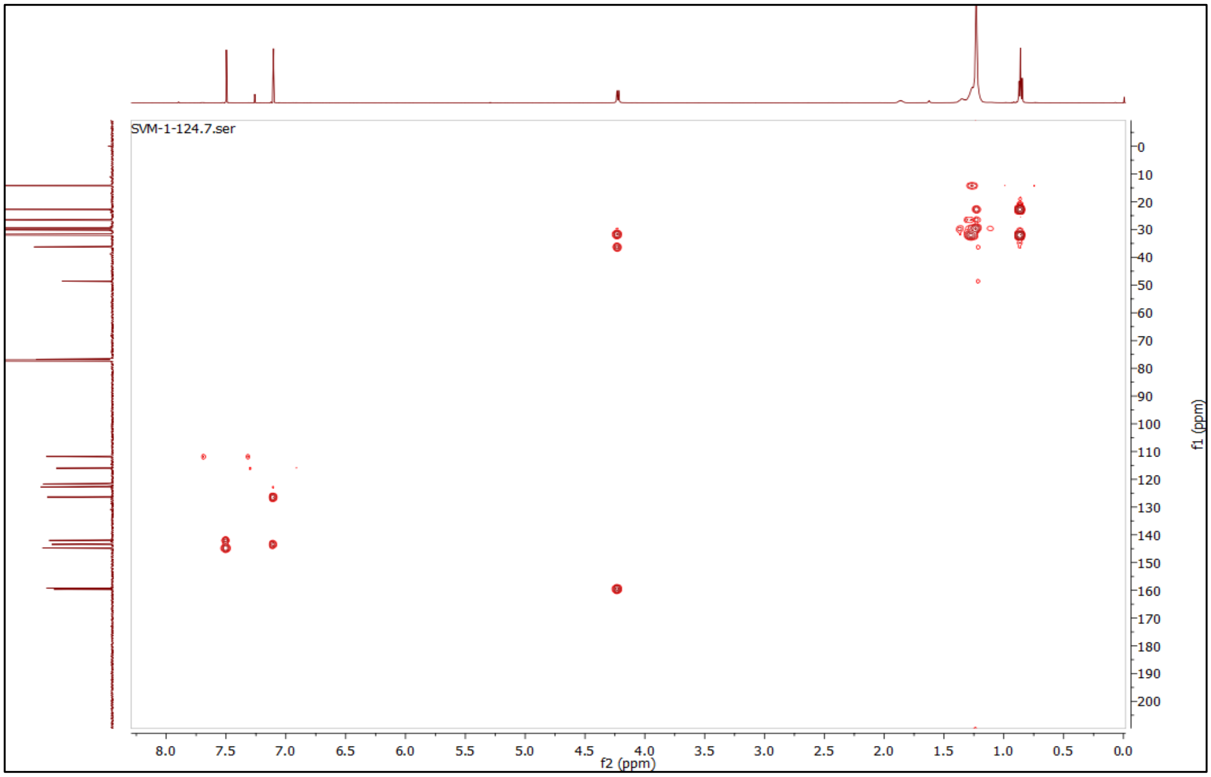

Figure S16. HMBC NMR spectra of **2** in $CDCl_3$, measured at 298 K.

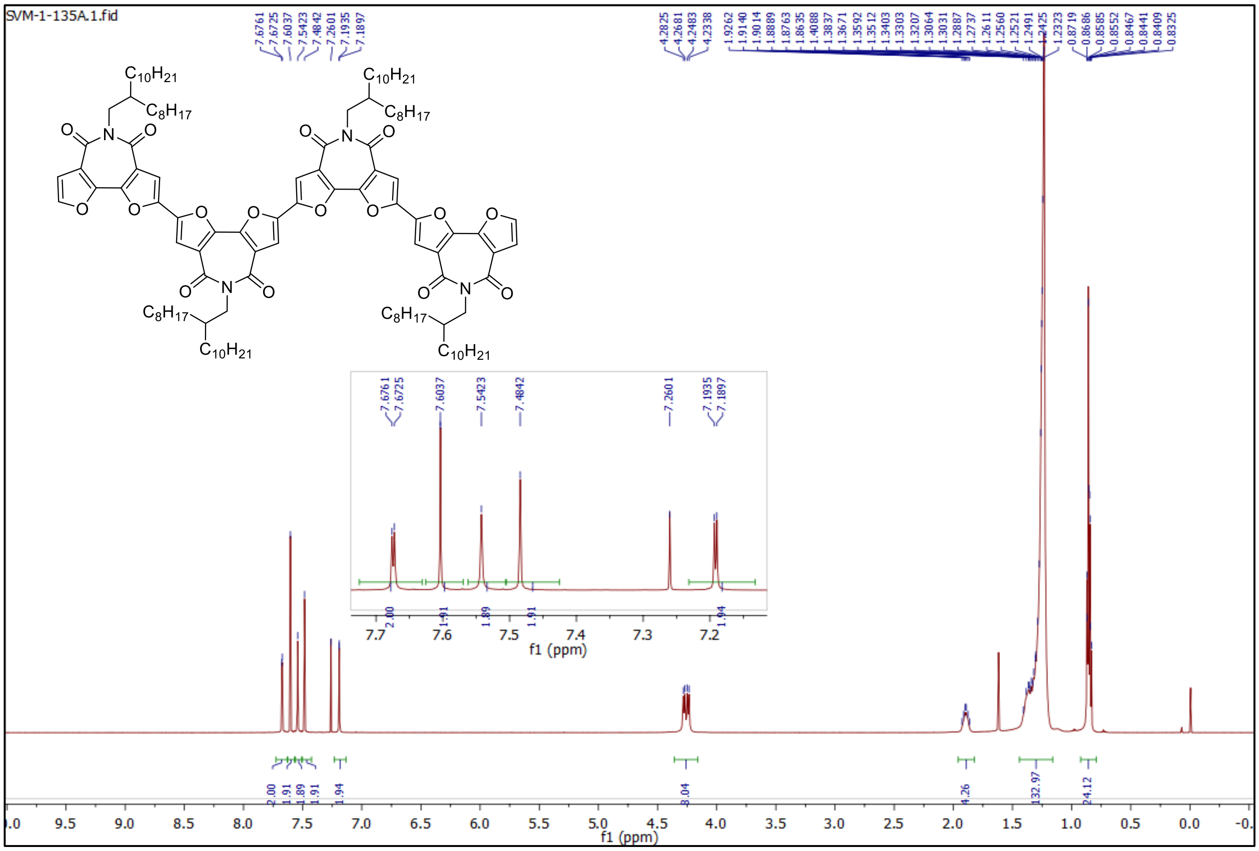

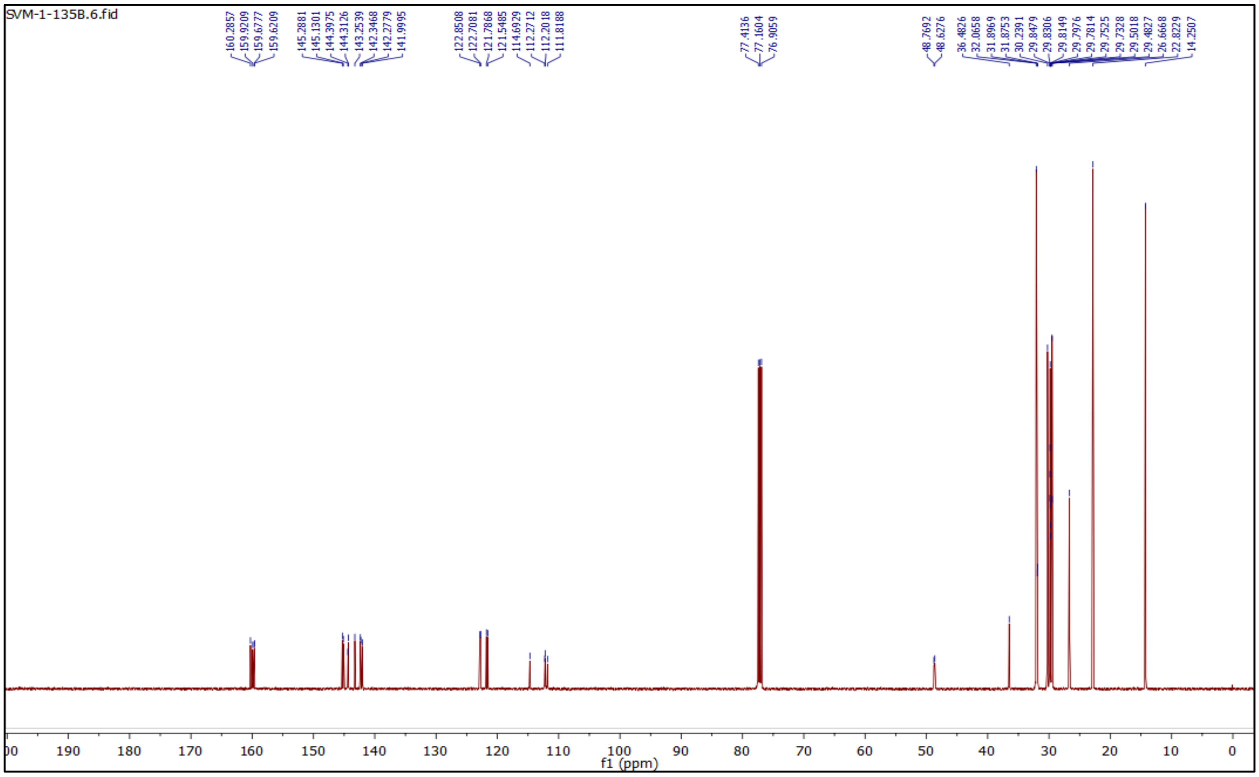

Figure S17. ^1H and ^{13}C NMR spectra of **L-4BFI** in $CDCl_3$, measured at 298 K.

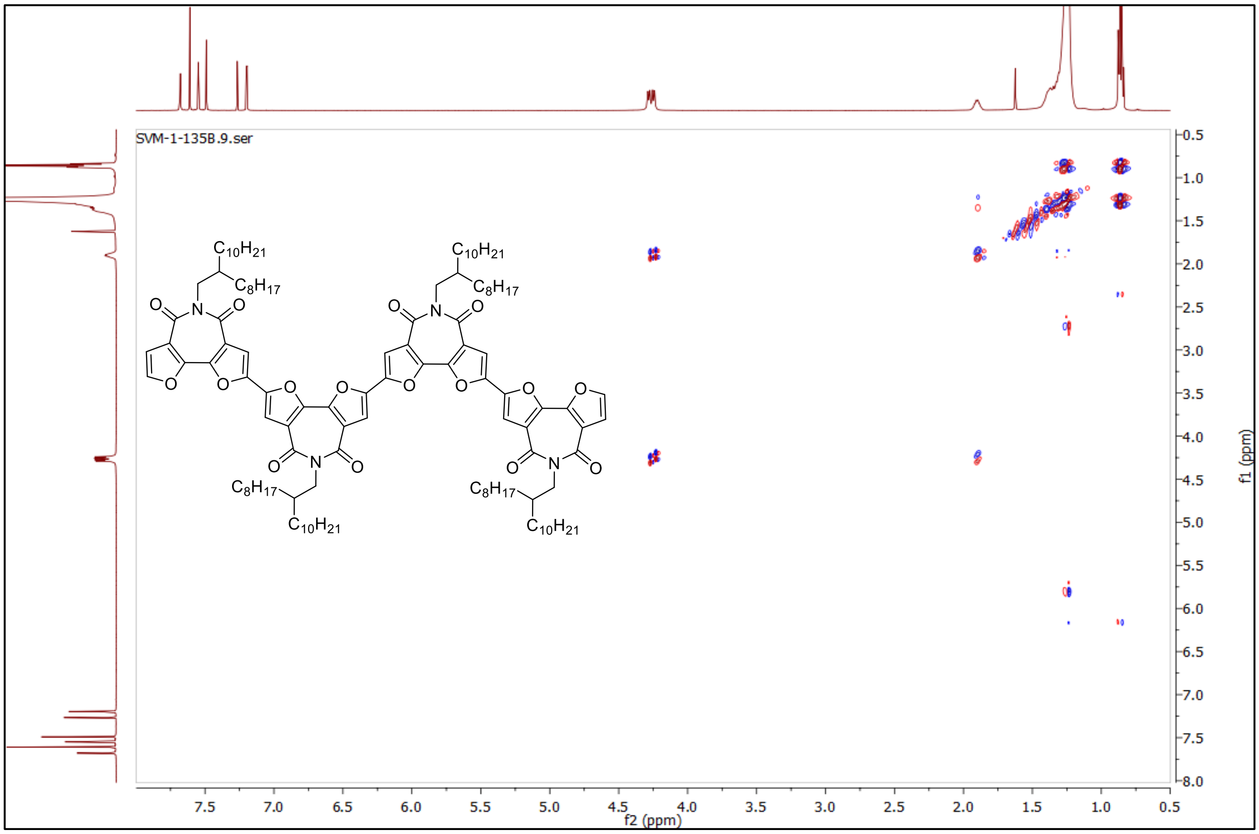

Figure S18. COSY NMR spectra of **L-4BFI** in $CDCl_3$, measured at 298 K.

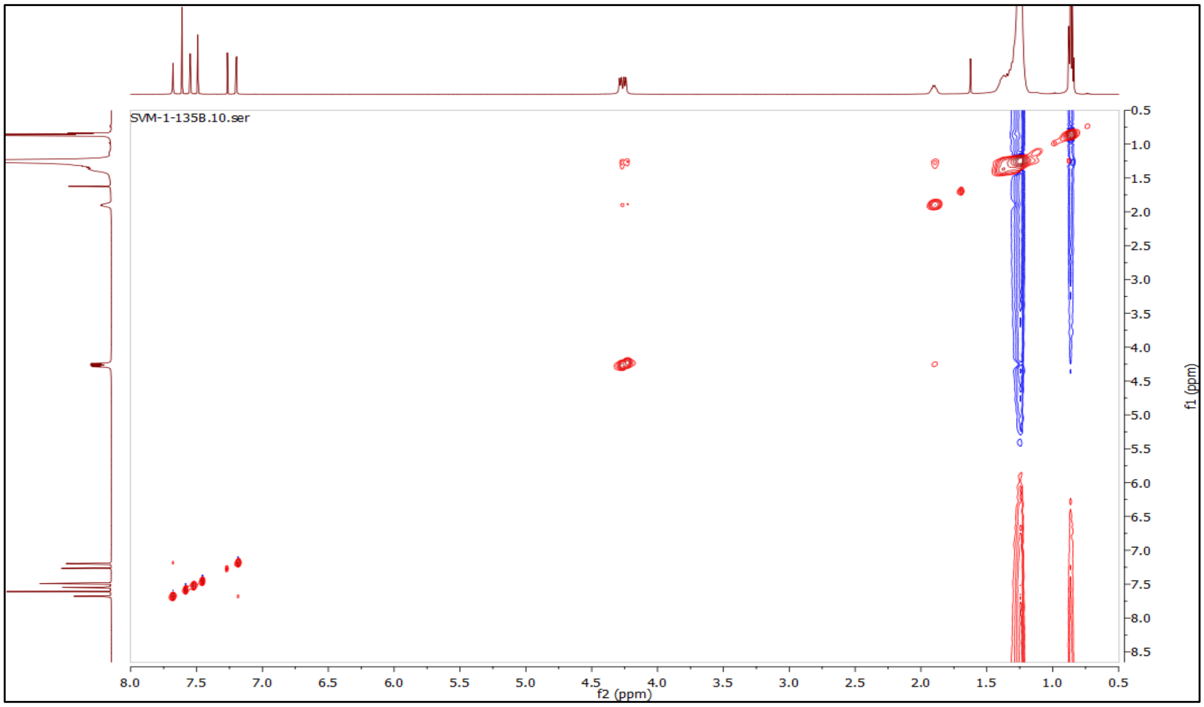

Figure S19. NOESY NMR spectra of **L-4BFI** in $CDCl_3$, measured at 298 K.

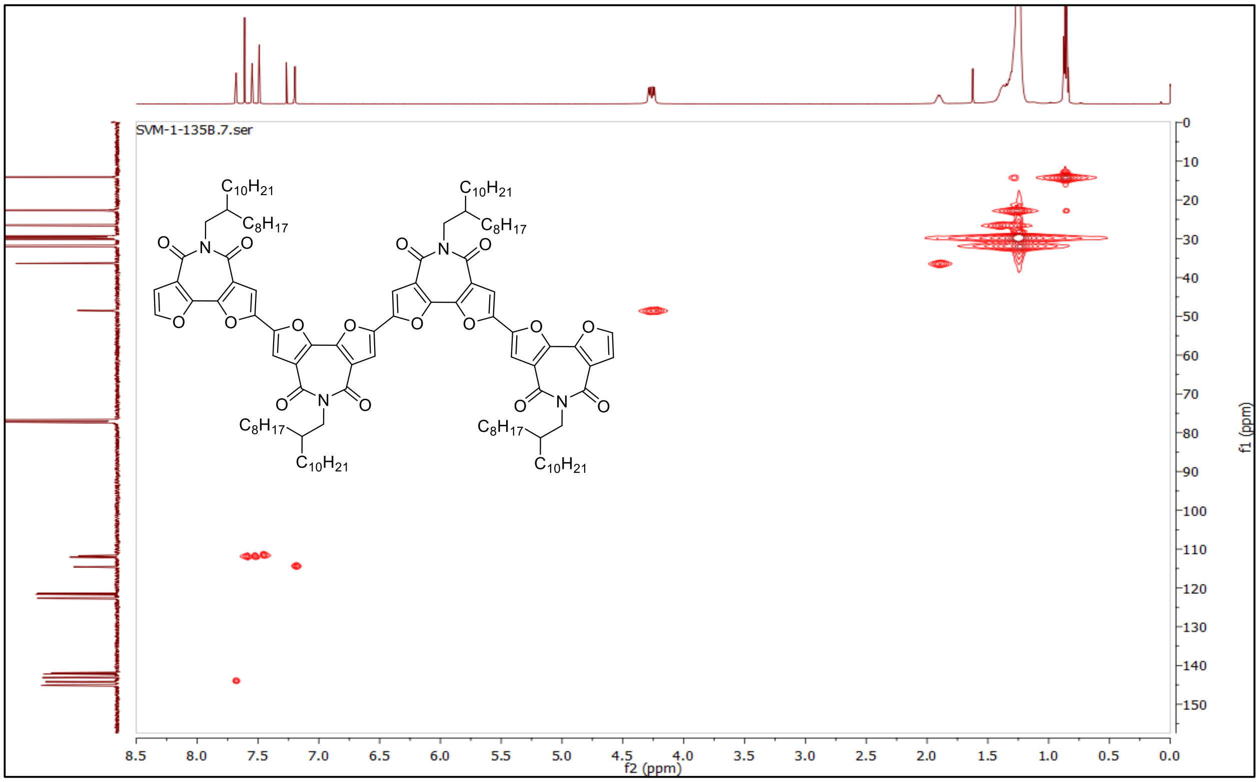

Figure S20. HSQC NMR spectra of **L-4BFI** in $CDCl_3$, measured at 298 K.

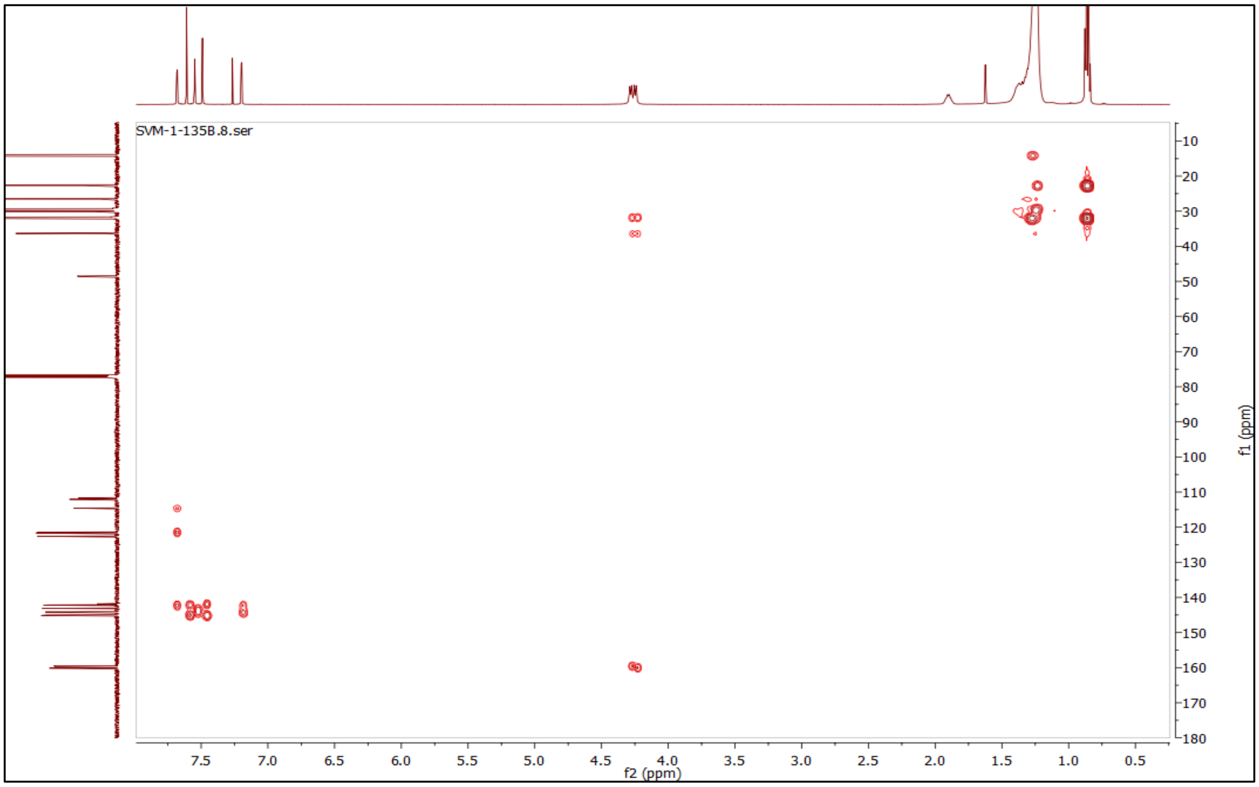

Figure S21. HMBC NMR spectra of **L-4BFI** in $CDCl_3$, measured at 298 K.

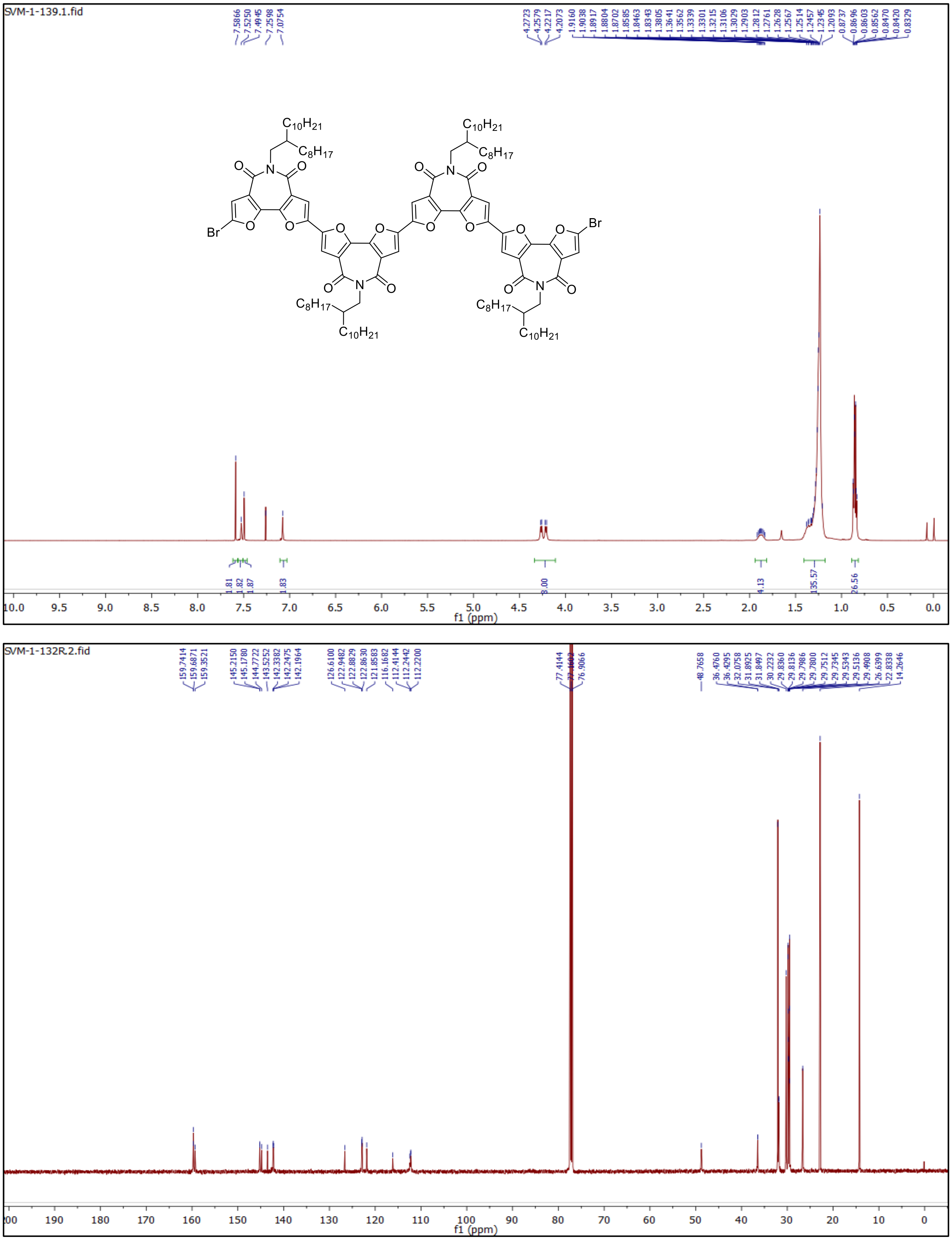

Figure S22. ^{1}H and ^{13}C NMR spectra of **3** in $CDCl_3$, measured at 298 K.

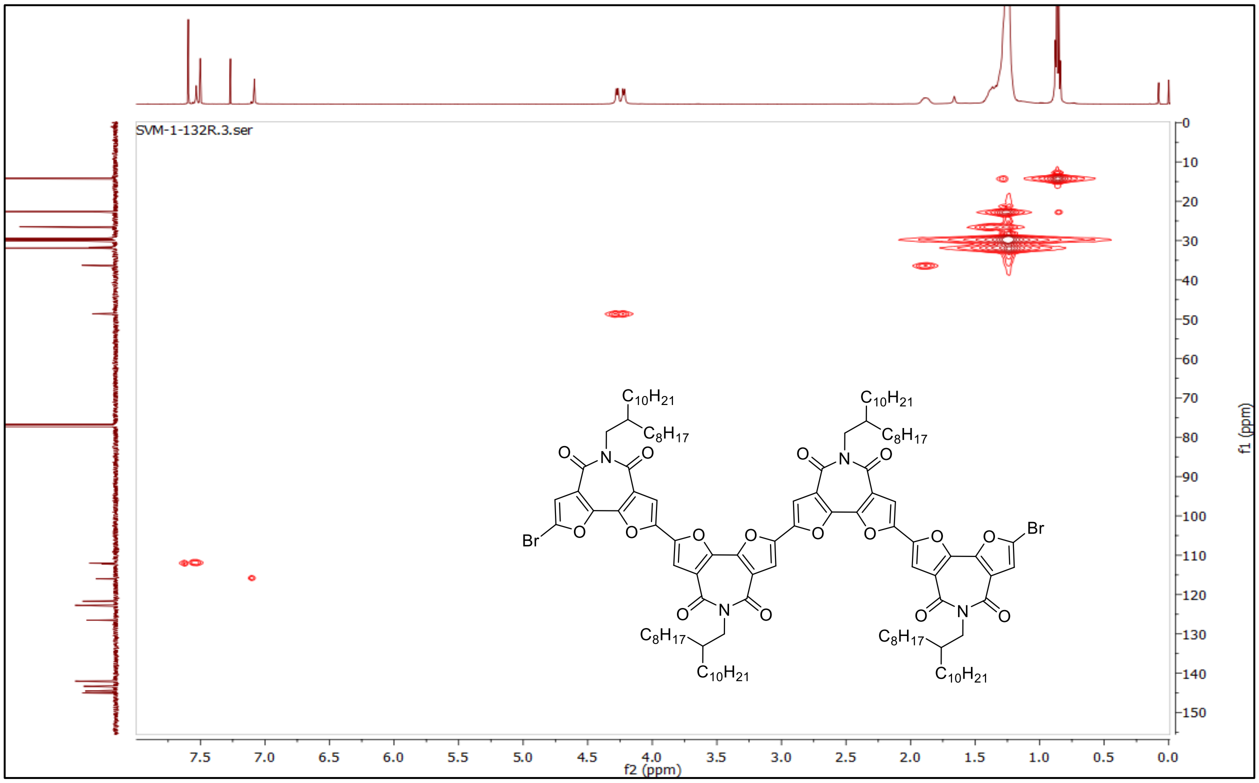

Figure S23. HSQC NMR spectra of **3** in $CDCl_3$, measured at 298 K.

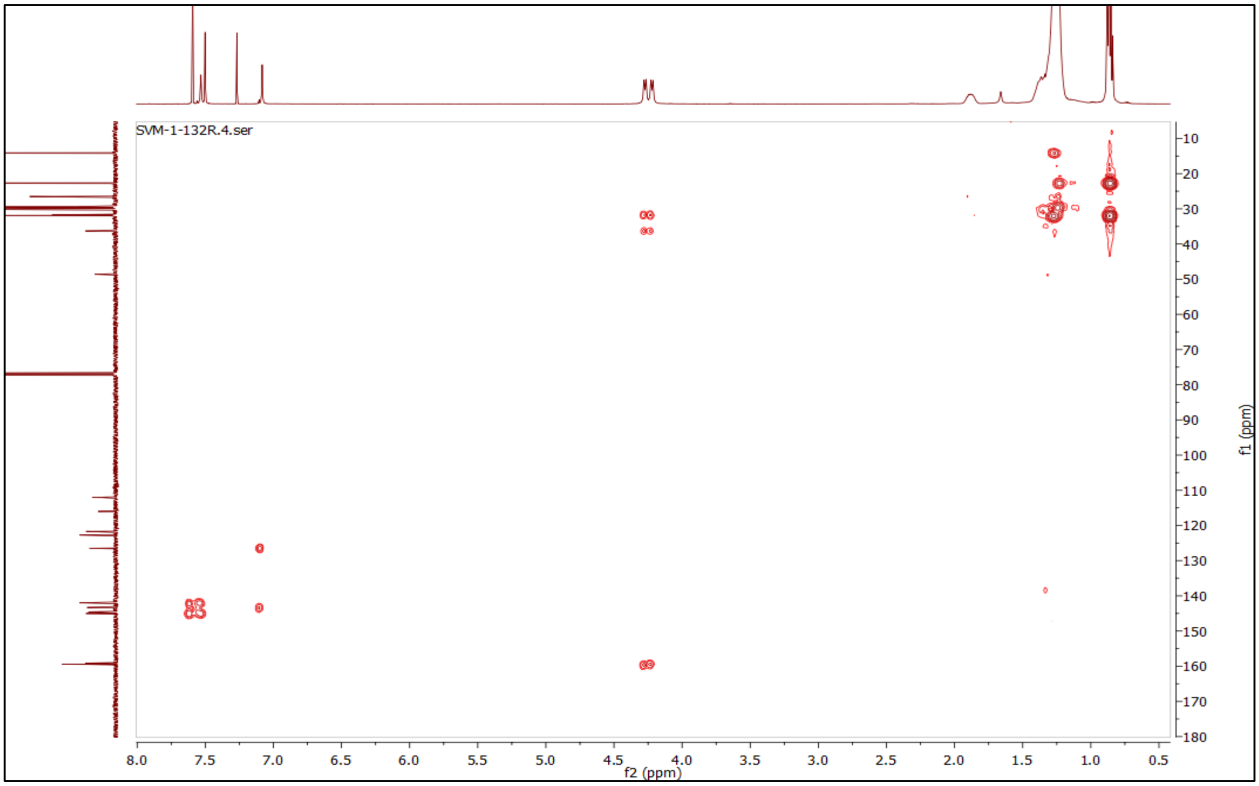

Figure S24. HMBC NMR spectra of **3** in $CDCl_3$, measured at 298 K.

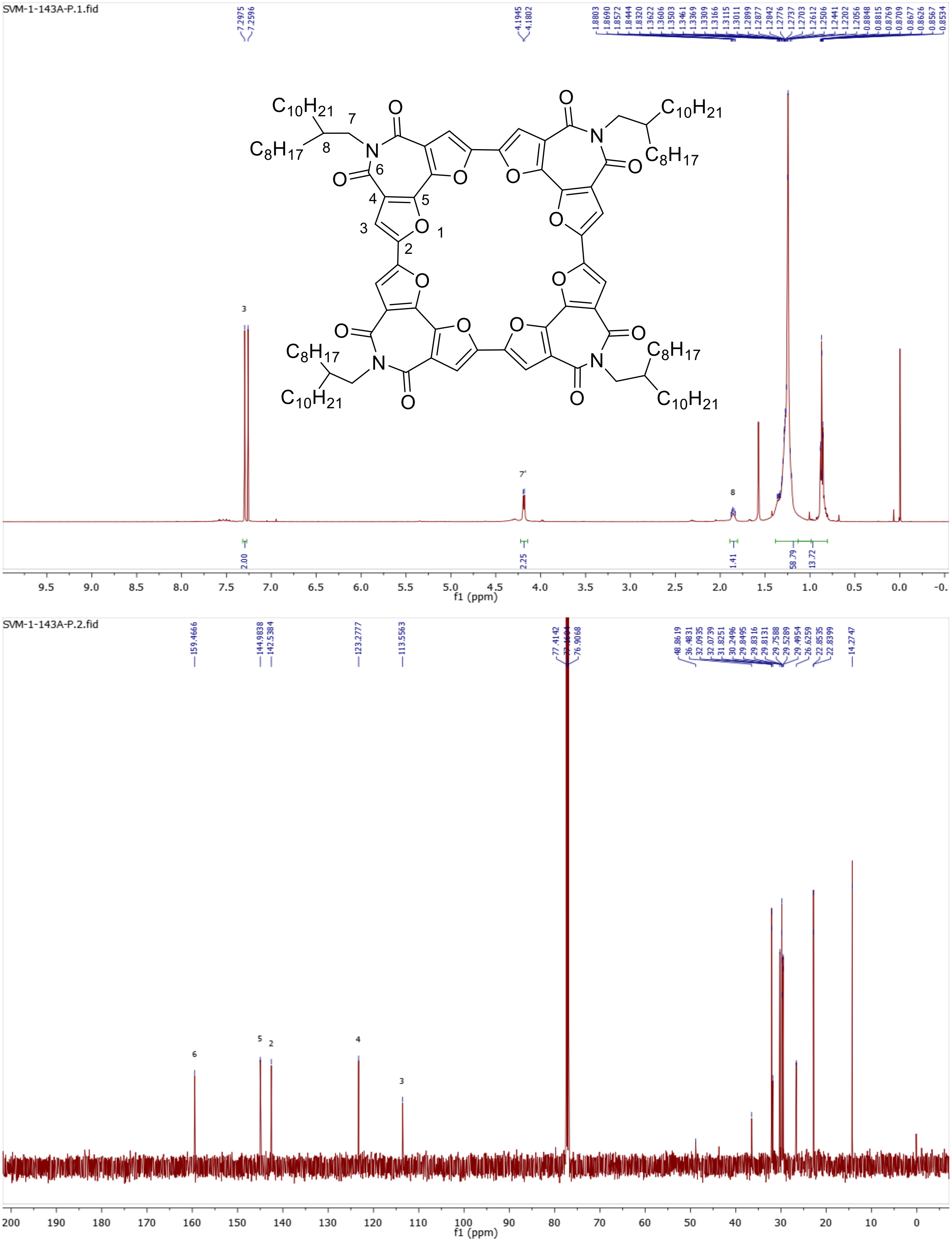

Figure S25. ^{1}H and ^{13}C NMR spectra of **C-4BFI** in $CDCl_3$, measured at 298 K.

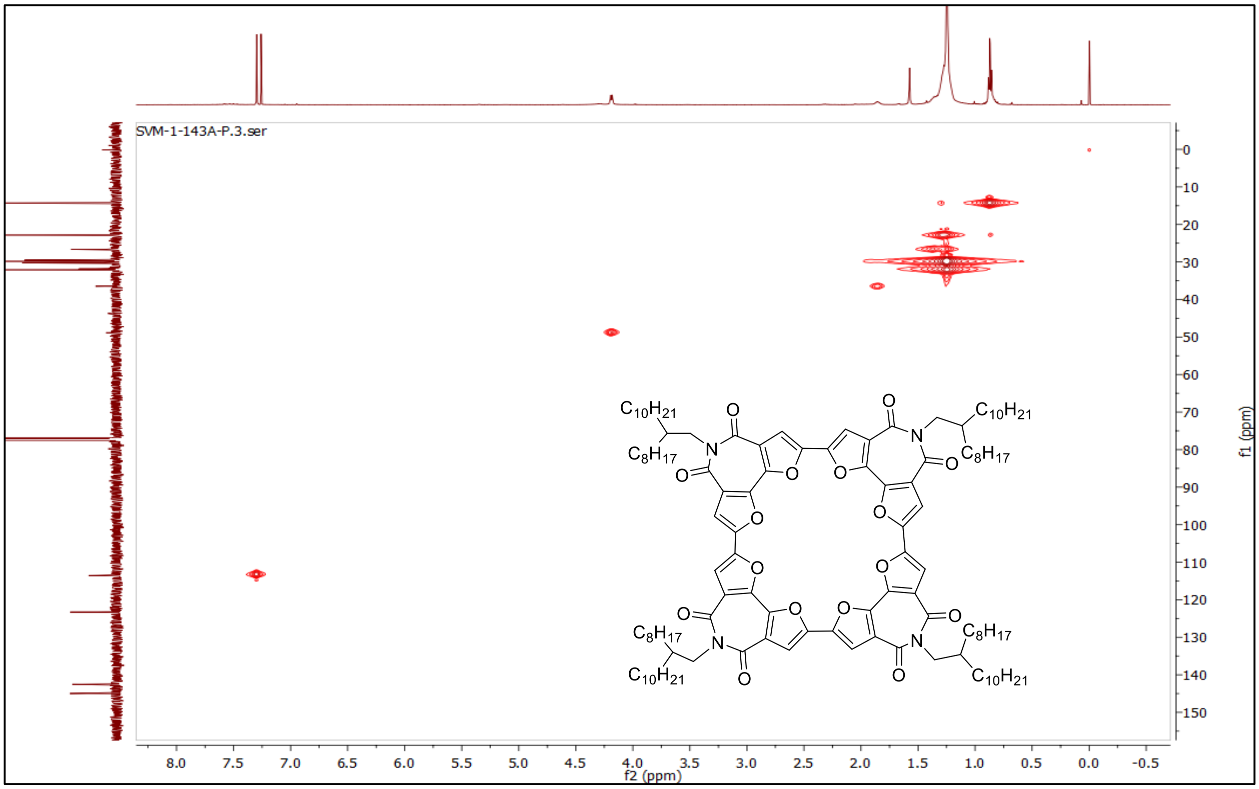

Figure S26. HSQC NMR spectra of **C-4BFI** in $CDCl_3$, measured at 298 K.

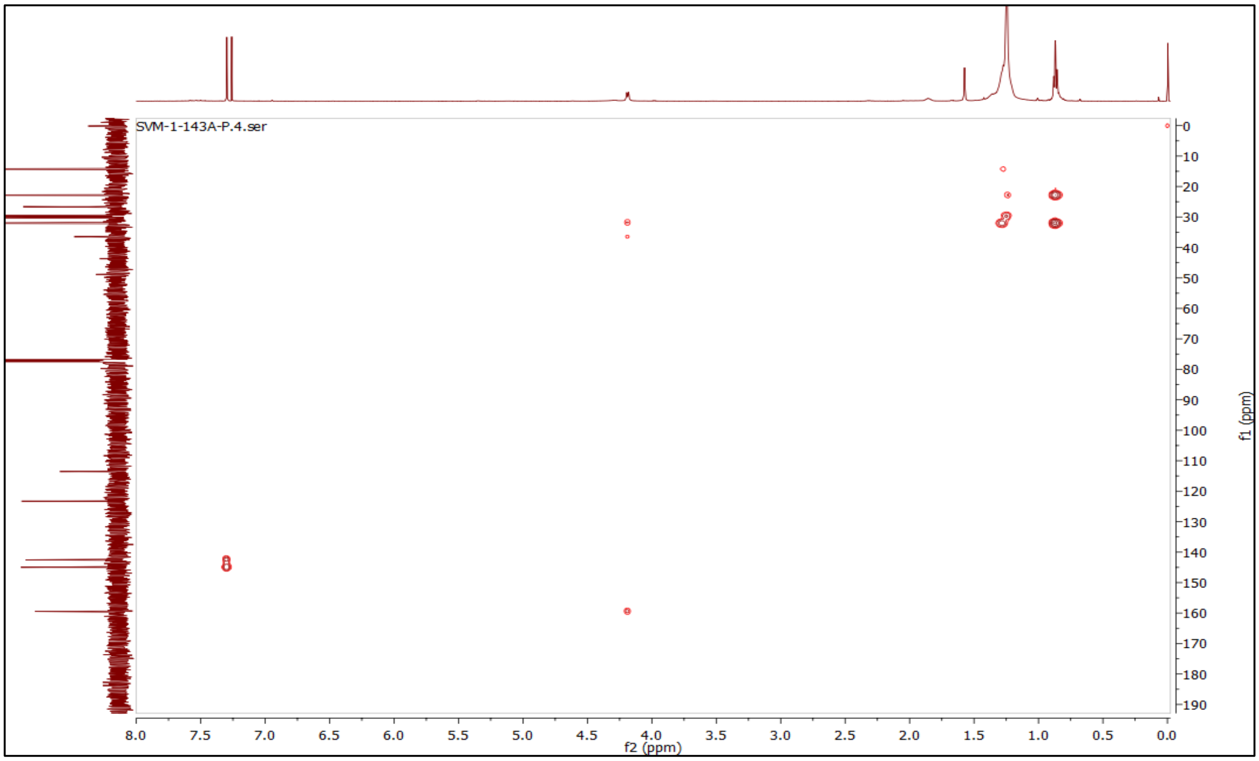

Figure S27. HMBC NMR spectra of **C-4BFI** in $CDCl_3$, measured at 298 K.

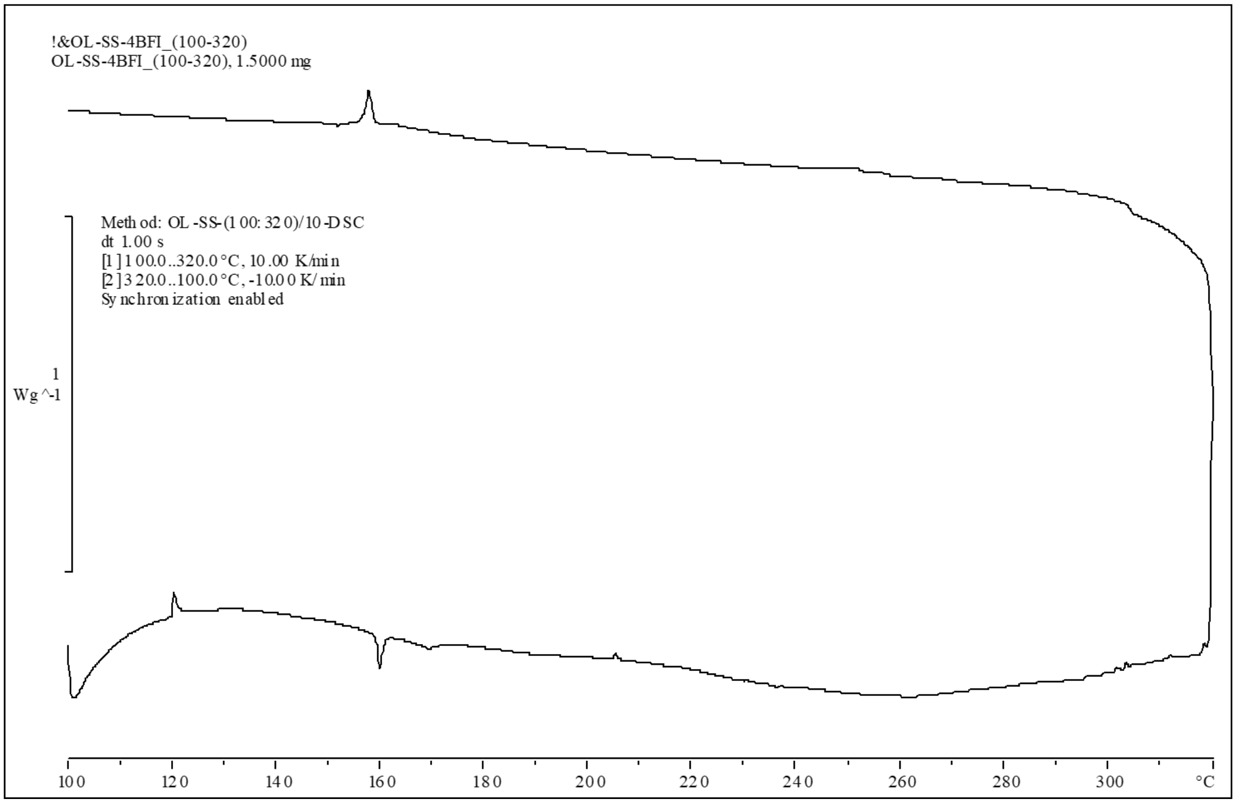

Figure S28. Differential Scanning Calorimetry (DSC) for **C-4BFI**. Scan rate: 10 °C/min.

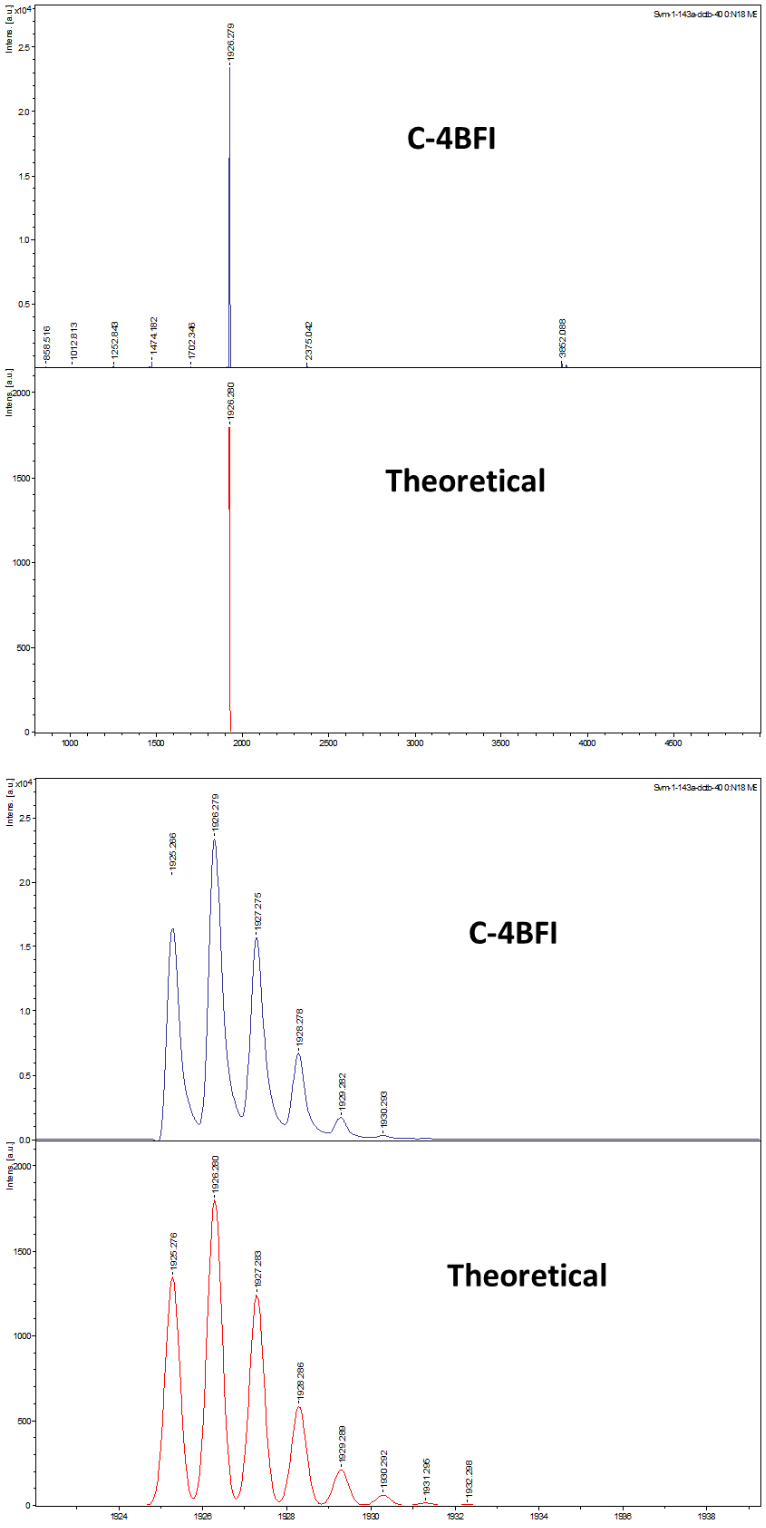

Figure S29. MALDI of **C-4BFI**.

S5. X-Ray Data Collection and Structure Refinement

	Macrocycle og8
X-ray source	ESRF ID23-1
Empirical formula	$C_{116}H_{162}N_4O_{16}$ + $3(C_3H_6O)$
Crystal description	Orange needle
Formula weight (g/mol)	2042.73
Temperature (K)	100
Wavelength (Å)	0.80
Crystal system	triclinic
Space group	P-1
a (Å)	4.830
b (Å)	22.780
c (Å)	28.510
α (°)	79.22
β (°)	88.33
γ (°)	86.44
Volume ($Å^3$)	3075
Z	1
Density calculated (Mg/m^3)	1.103
Absorption coefficient (mm^{-1})	0.094
F(000)	1110
Crystal size (mm^3)	$0.200^X0.016^X0.002$
Theta range for data collection (°)	1.026 to 30.202
Reflection collected (Unique)	47875(8537)
R int	0.0559
Completeness to theta 28.68	74.1%
Data\restraints\parameters	8537 \ 82 \ 683
Goodness-of-fit on F^2	1.133
Final R [I>2σ(I)]	R1=0.0970, wR2=0.2789
R (all data)	R1=0.1301, wR2=0.3072
Largest diff. peak and hole ($e^{\cdot}$ $Å^{-3}$)	0.493 and -0.456

A small needle-like crystal of C-4BFI was coated in Hampton Research Paratone oil, mounted on a cryo-loop and flash frozen in LN for transport to the ESRF synchrotron for data collection. Data were collected at 100K, to atomic resolution at beam-line ID23-1 using MXCube and EDNA strategy and processed with XDS auto-processing. The molecular structure was solved using SHELXT-2016/4 and fully refined with anisotropic temperature factors for all non-hydrogen atoms using SHELXL-2016/4. Structure and data have been deposited with the CCDC number 1892379.

S6. Computational Details

All calculations were carried out with the Gaussian 09 series of programs[2] using density function theory (DFT). Becke's three-parameter exchange functional combined with the Lee-Yang-Parr correlation functional (B3LYP) and with the 6-311G(d) basis set was used for all calculations.[3-6] The B3LYP functional was chosen in order to conduct a valid comparison with previous computational studies of analogous systems.[7, 8] **nF**, **nT**, **nCF**, and **nCT** are defined as depicted in chart S1, and the data taken from a previous work published by our group.[9] No symmetry restrictions were applied to any of the calculations, unless explicitly specified otherwise. The optimal geometries for all structures were confirmed as minima by frequency calculations. No negative frequencies were found for any optimized structures presented in this work. **C-4BFI-H** was calculated using both no-symmetry constrains, and with restriction to D_{4h} point group. With these results, we have calculated the planarization energy of the optimized structure; since the resulting energy difference of 0.2 kcal/mol is within the margin of error for this level of theory. The same calculation was applied to **C-4BTI-H**, resulting in planarization energy of 17 kcal/mol.

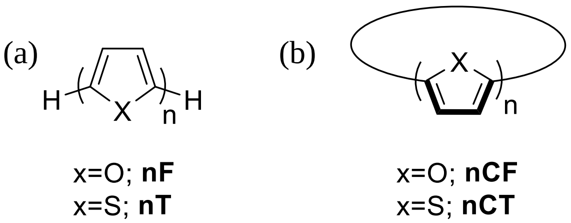

Chart S1. Structures of (a) linear furan oligomers and (b) macrocyclic furan oligomers.

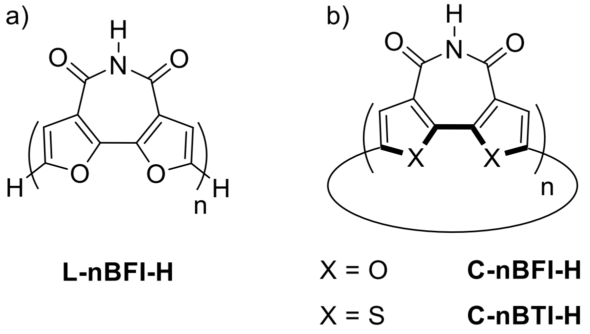

Chart S2. Structures of (a) linear **BFI** oligomers and (b) macrocyclic **BFI** oligomers.

S6.1. Absolute Energies of Neutral Structures

Table S1. Absolute energies for all uncharged structures presented in this work.

Molecule	Energy (Hartree)
**L-1BFI-H**	-739.8876987
**L-2BFI-H**	-1478.589852
**L-3BFI-H**	-2217.291702
**L-4BFI-H**	-2955.993414
**L-5BFI-H**	-3694.695241
**L-6BFI-H**	-4433.397026
**L-7BFI-H**	-5172.098734
**L-10BFI-H**	-7388.203974
**L-15BFI-H**	-11081.71266
**C-3BFI-H**	-2216.069355
**C-4BFI-H**	-2954.79176
**C-4BFI-H** (D_{4h} symmetry)	-2954.791435
**C-4BFI-(*sec*-Bu)**	-3583.884622
**C-5BFI-H**	-3693.469036
**Poly-2BFI-H (PBC)**	-1477.40348
**C-4BTI-H**	-5538.5735692
**C-4BTI-H** (D_{4h} symmetry)	-5538.545729

S6.2. Optimized Structures of Selected Macrocycles

a) b)

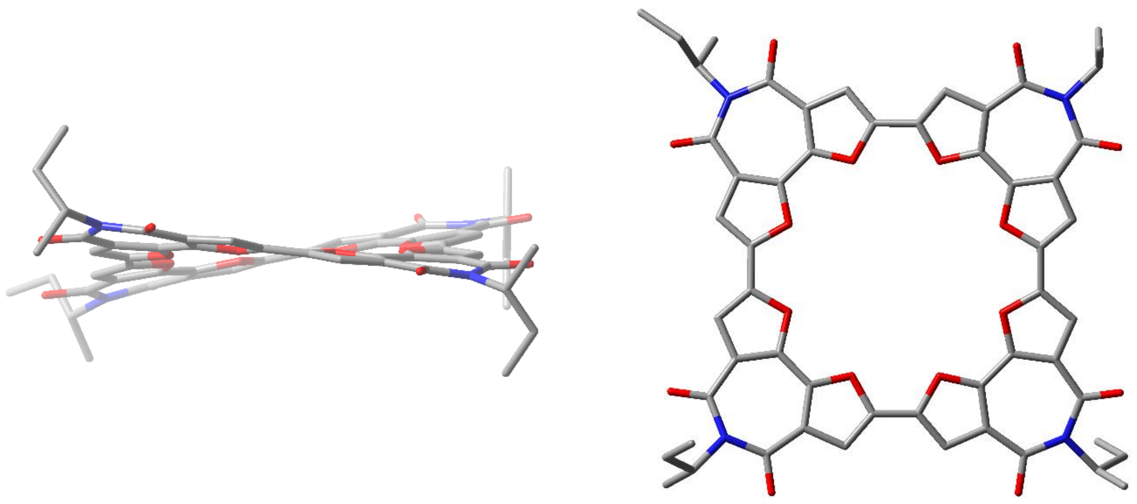

Figure S30. Optimized structure of **C-4BFI-(*sec*-Bu)** from (a) side view and (b) top view. Hydrogens are omitted for clarity.

a) b)

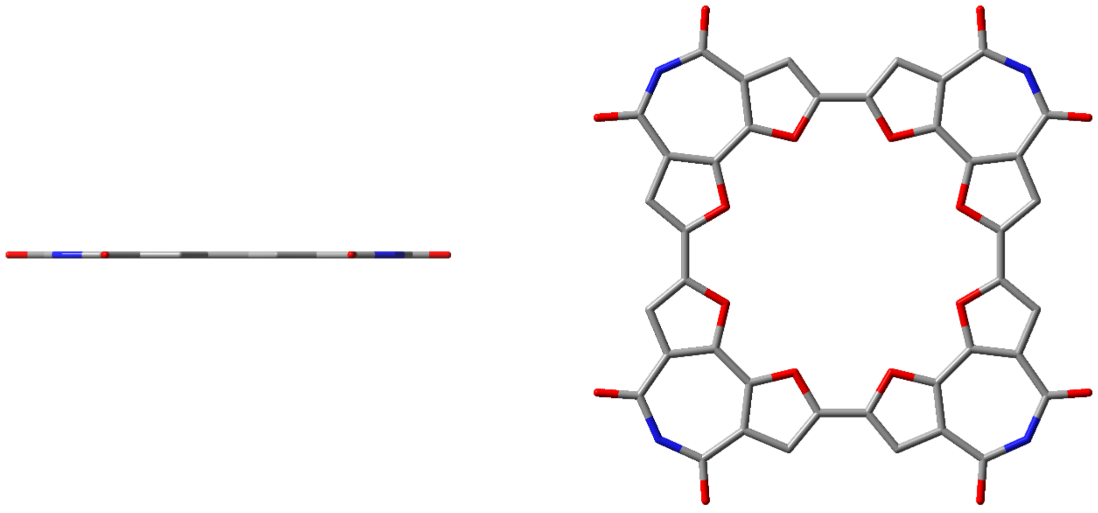

Figure S31. Optimized structure of **C-4BFI-H** (D_{4h} symmetry) from (a) side view and (b) top view. Hydrogens are omitted for clarity.

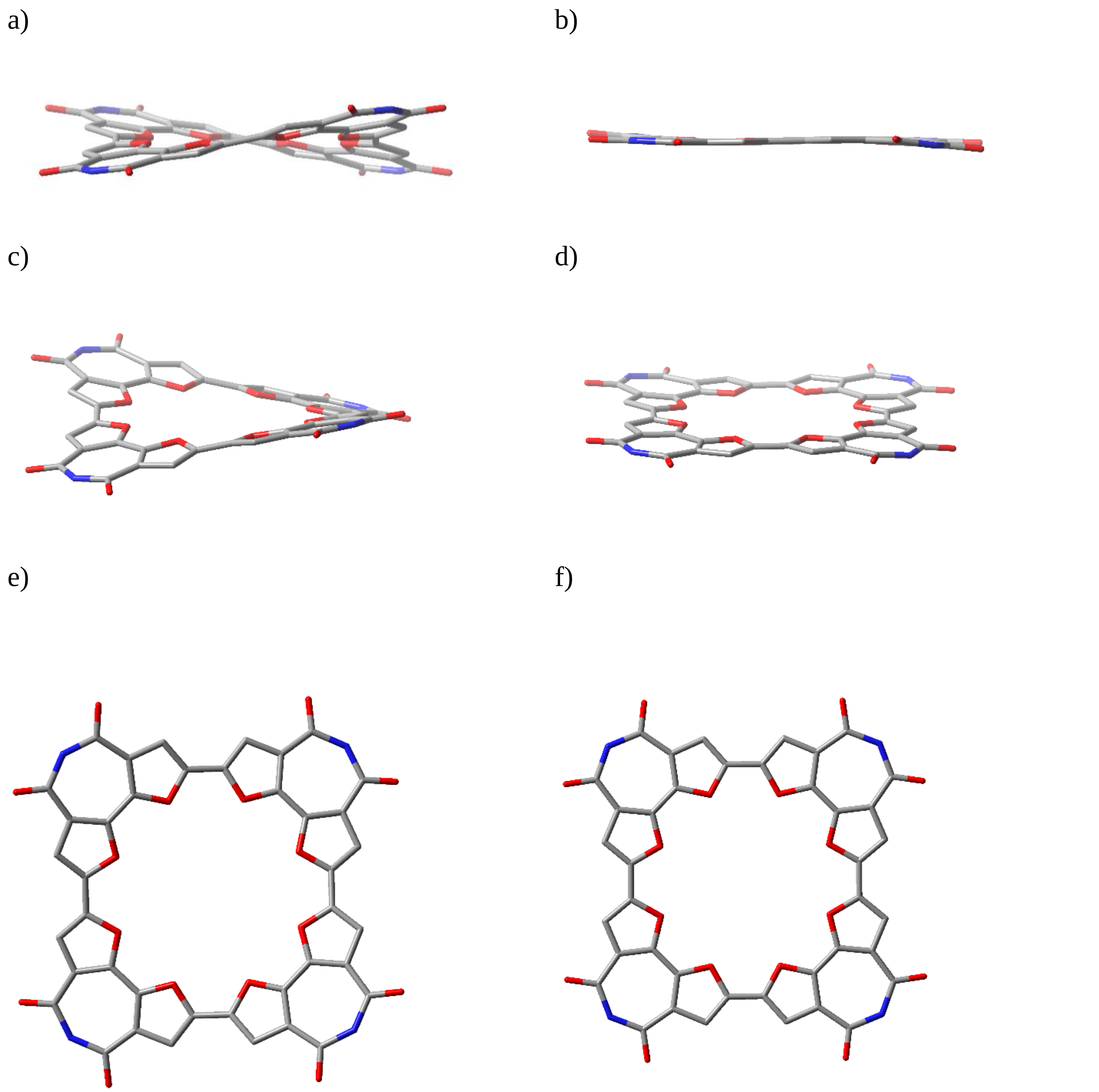

Figure S32. The structure of (a,c,e) **C-4BFI-H** (optimized) and of (b,d,f) **C-4BFI** (taken from crystal structure). Hydrogens and alkyl chains are omitted for clarity.

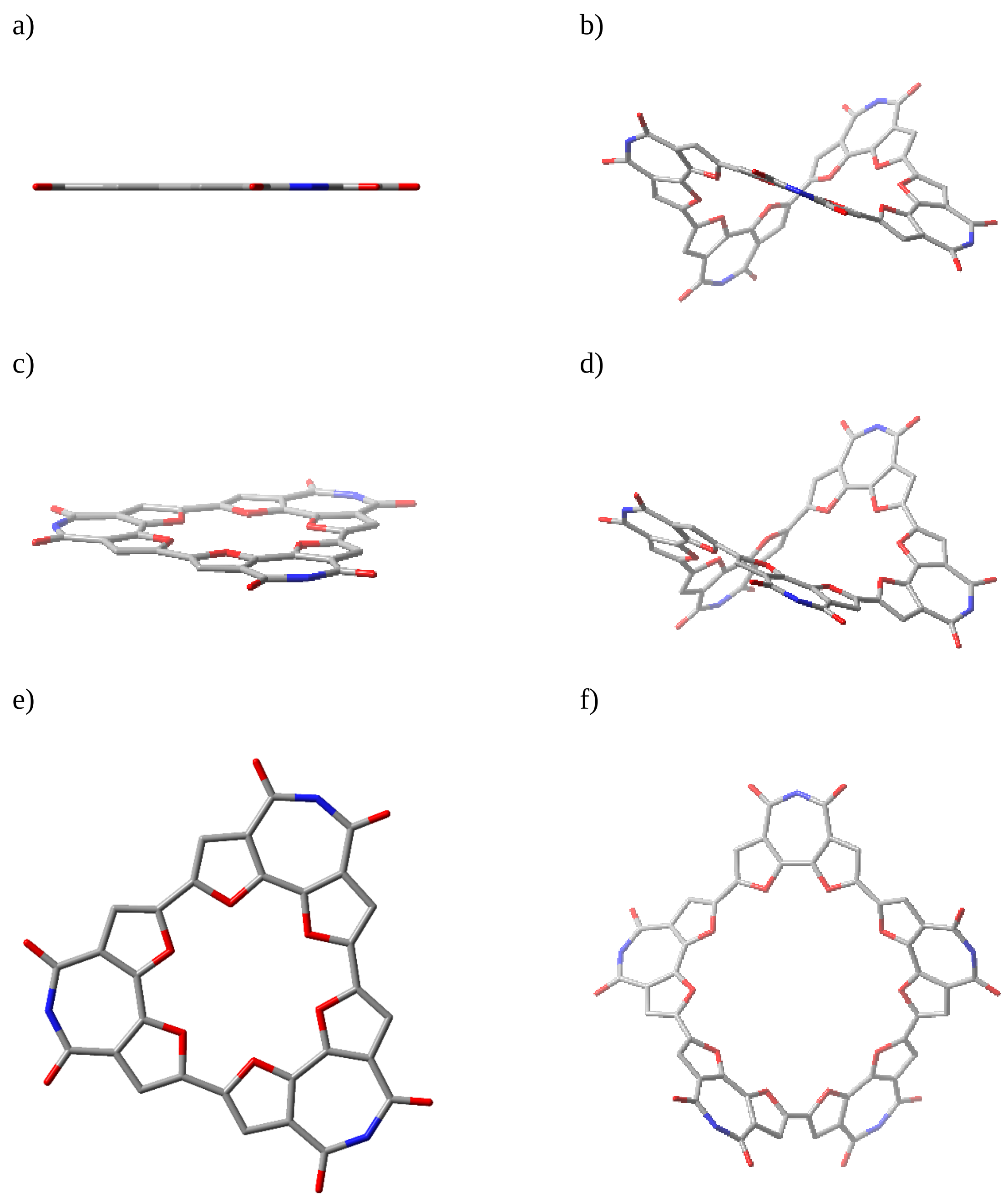

Figure S33. Optimized structure of (a,c,e) **C-3BFI-H** and (b,d,f) **C-5BFI-H**. Hydrogens are omitted for clarity.

a) b)

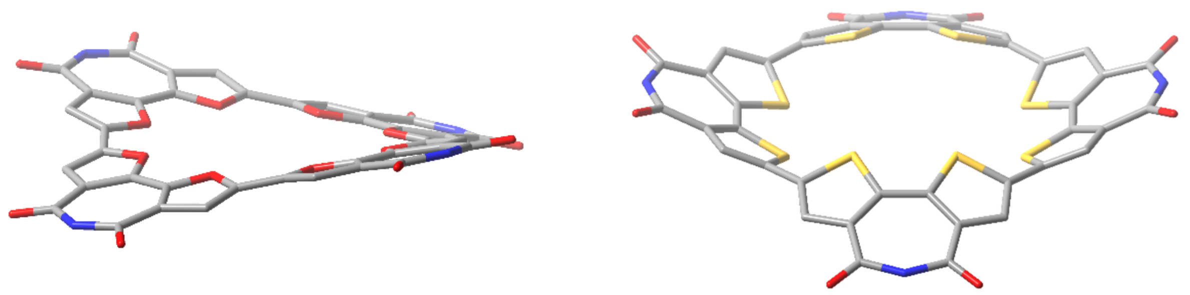

Figure S34. Optimized structure of (a) **C-4BFI-H** and (b) **C-4BTI-H**. Hydrogens are omitted for clarity. The calculated planarization energy for **C-4BFI-H** is 0.2 kcal/mol and for **C-4BTI-H** is 17.5 kcal/mol.

S6.3. Frontier Molecular Orbitals for Selected Macrocycles

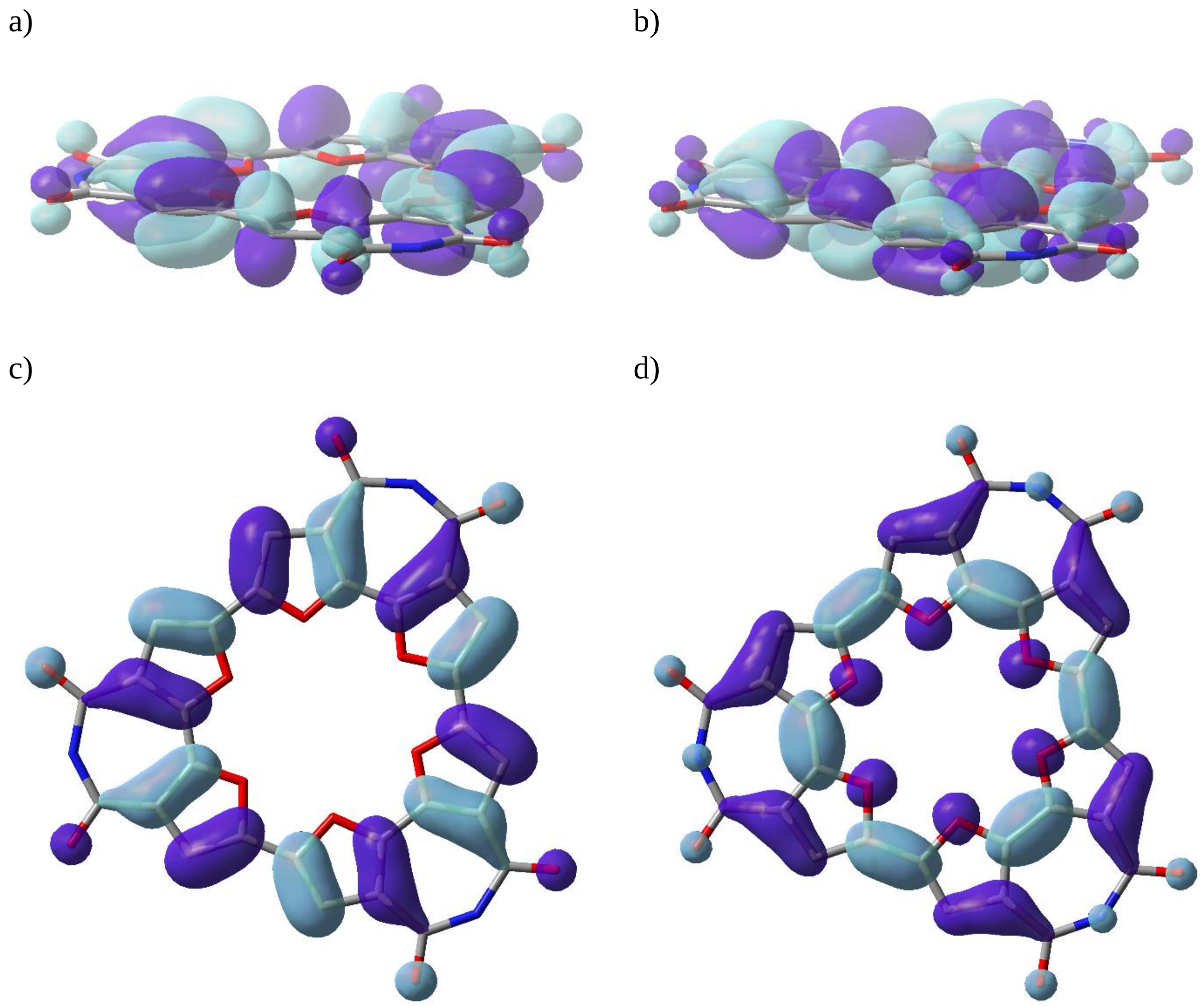

Figure S35. (a,c) HOMO and (b,d) LUMO of **C-3BFI-H**.

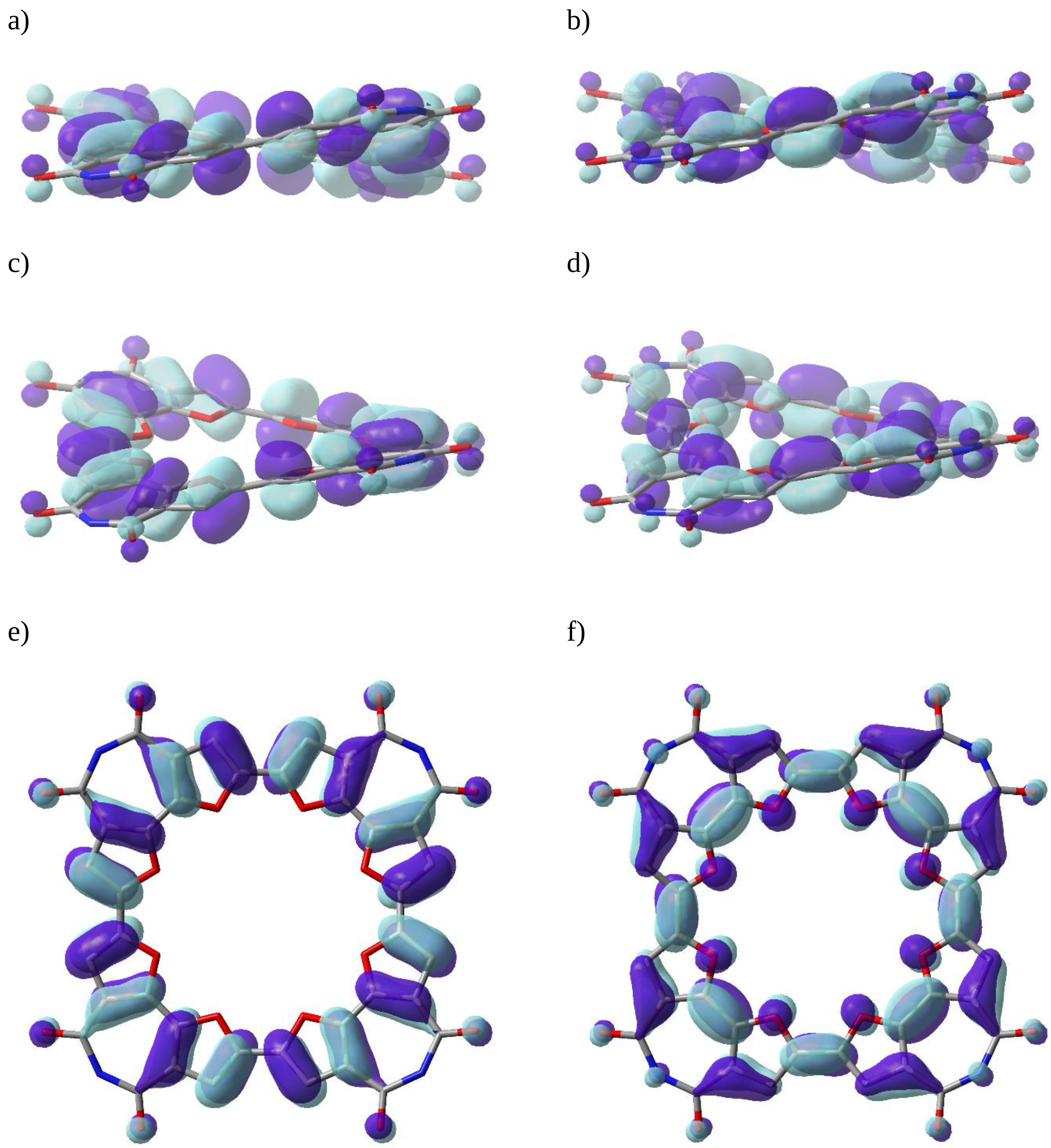

Figure S36. (a,c,e) HOMO and (b,d,f) LUMO of **C-4BFI-H**.

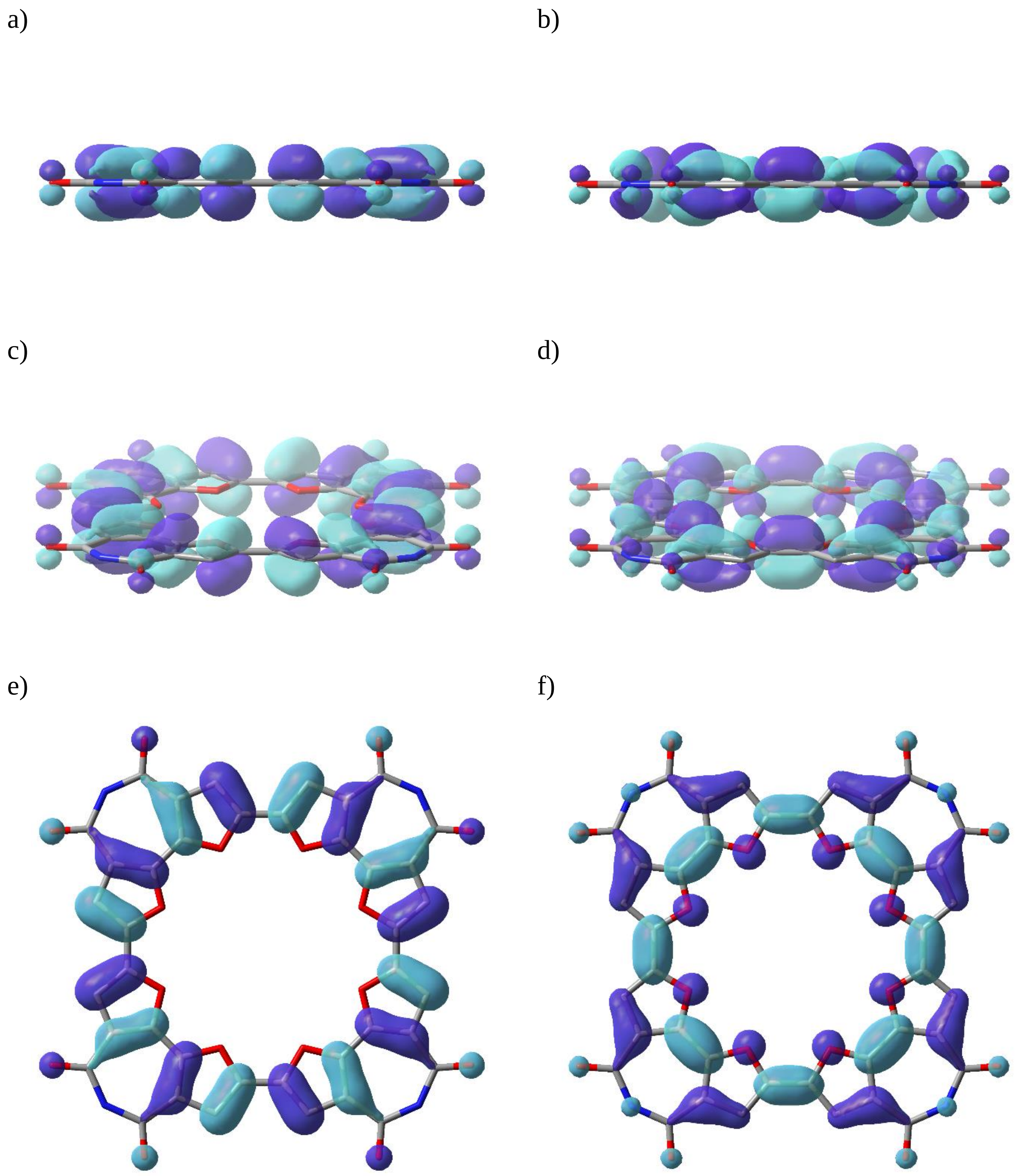

Figure S37. (a,c,e) HOMO and (b,d,f) LUMO of **C-4BFI-H** (D_{4h} symmetry).

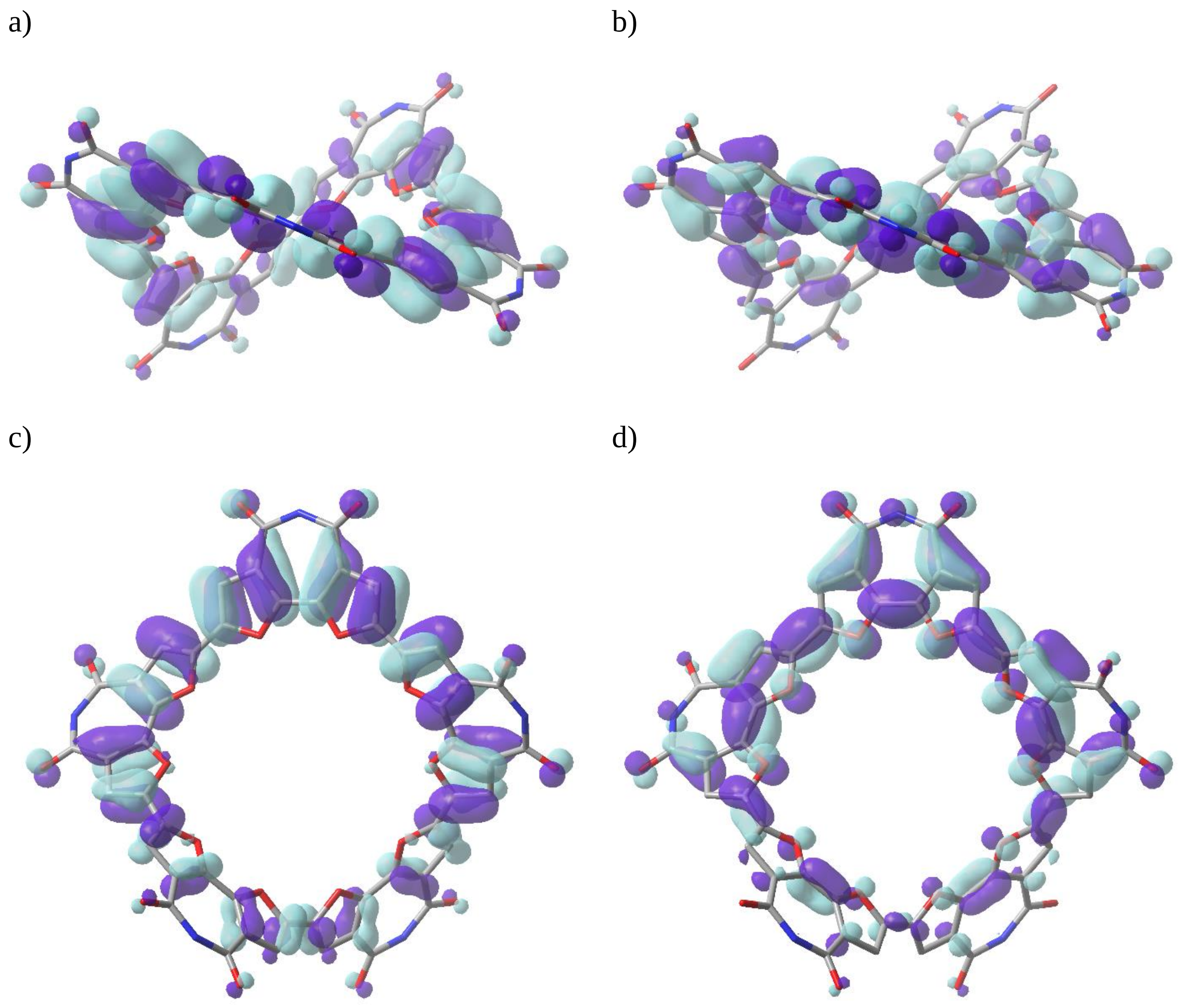

Figure S38. (a,c) HOMO and (b,d) LUMO of **C-5BFI-H**.

S6.4. Calculated HOMO-LUMO gaps for selected oligomers

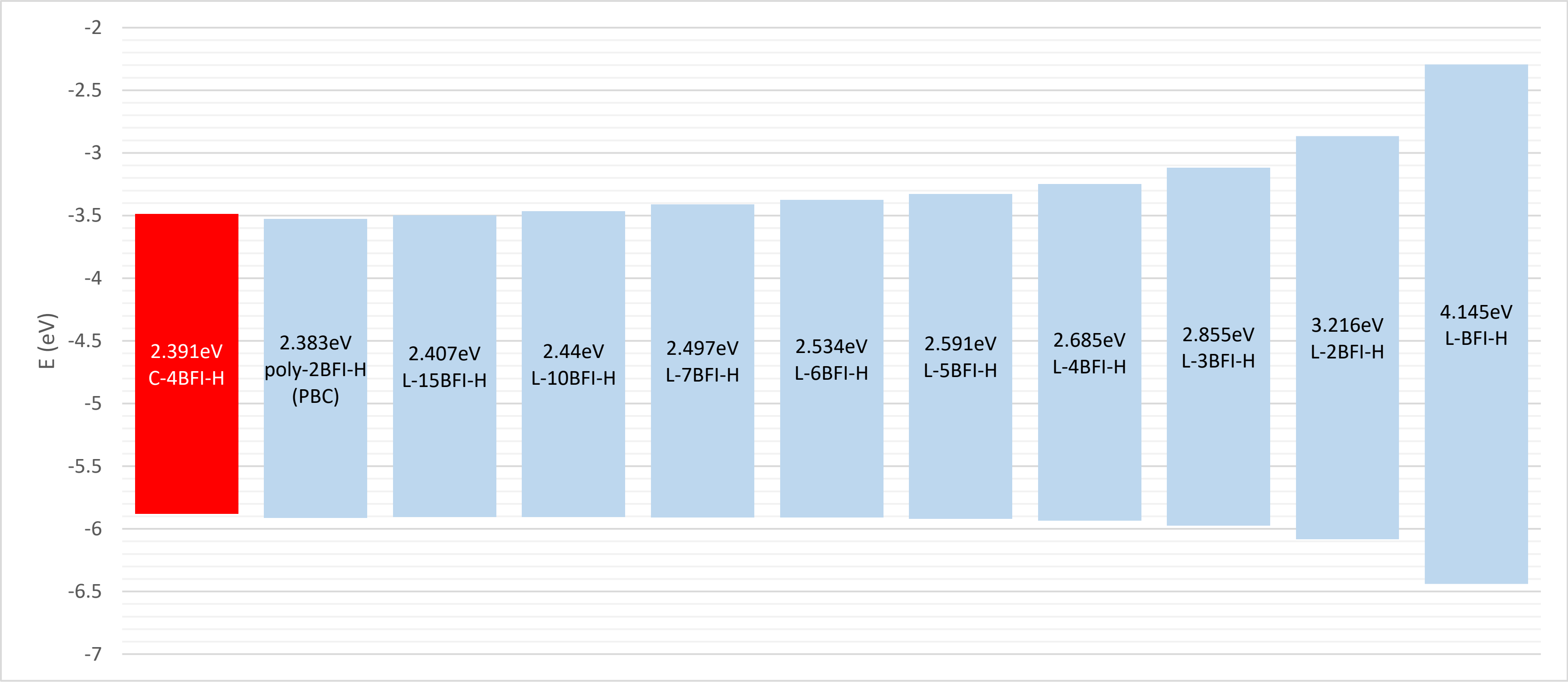

Figure S39. computational HOMO-LUMO gaps for **L-(1–7,10,15)BFI**, **poly-2BFI-H**, and **C-4BFI-H**.

Table S2. computational HOMO, LUMO and gap energies for selected oligomers.

	LUMO (eV)	HOMO (eV)	Gap (eV)
**8F**	-1.912	-4.517	2.781
**8CF**	-1.952	-4.733	2.605
**C-4BFI-H**	-3.489	-5.880	2.391
**C-4BFI-H (D_{4h} symmetry)**[1]	-3.537	-5.794	2.257
**Poly-2BFI-H**[2]	-3.529	-5.912	2.383
**L-15BFI-H**	-3.499	-5.906	2.407
**L-10BFI-H**	-3.465	-5.904	2.440
**L-7BFI-H**	-3.410	-5.907	2.497
**L-6BFI-H**	-3.376	-5.910	2.534
**L-5BFI-H**	-3.327	-5.918	2.591
**L-4BFI-H**	-3.250	-5.935	2.685
**L-3BFI-H**	-3.120	-5.975	2.855
**L-2BFI-H**	-2.866	-6.082	3.216
**L-1BFI-H**	-2.295	-6.440	4.145
**C-4BTI-H**	-6.441	-3.702	2.739
**C-4BTI-H (D_{4h} symmetry)**	-6.233	-3.988	2.245

S6.5. TD-DFT calculation of **C-4BFI-H** (B3LYP/6-311g(d))

Excitation energies and oscillator strengths:

Excited State 1: Singlet-A 1.8542 eV 668.66 nm f=0.0000 <S**2>=0.000

204 -> 205 0.70217

---

[1] HOCO and LUCO values.

This state for optimization and/or second-order correction.

Total Energy, E(TD-HF/TD-KS) = -2954.72361593

Copying the excited state density for this state as the 1-particle RhoCI density.

Excited State 2: Singlet-A 2.6567 eV 466.68 nm f=0.2225 <S**2>=0.000

203 -> 205 0.29030

204 -> 206 0.64259

Excited State 3: Singlet-A 2.6568 eV 466.67 nm f=0.2225 <S**2>=0.000

202 -> 205 -0.29031

204 -> 207 0.64259

Excited State 4: Singlet-A 3.0351 eV 408.50 nm f=1.3670 <S**2>=0.000

203 -> 205 0.64079

204 -> 206 -0.29191

Excited State 5: Singlet-A 3.0351 eV 408.50 nm f=1.3670 <S**2>=0.000

202 -> 205 0.64079

204 -> 207 0.29192

S7. Experimental Absorption, Emission, and Excitation Spectra

We found that spectra of **C-4BFI** in chloroform and in hexane in similar concentrations displayed different behaviors. The absorption spectra in chloroform are uniform in shape (Figure S40–41), allowing us to calculate the extinction coefficient with a small error (ca. 2%, Figure S42); in contrast, the spectra in hexane exhibit both changes in the relative intensities of the peaks, and a hypsochromic shift in λ_{max} as the concentration of the macrocycle increases (Figure S43).

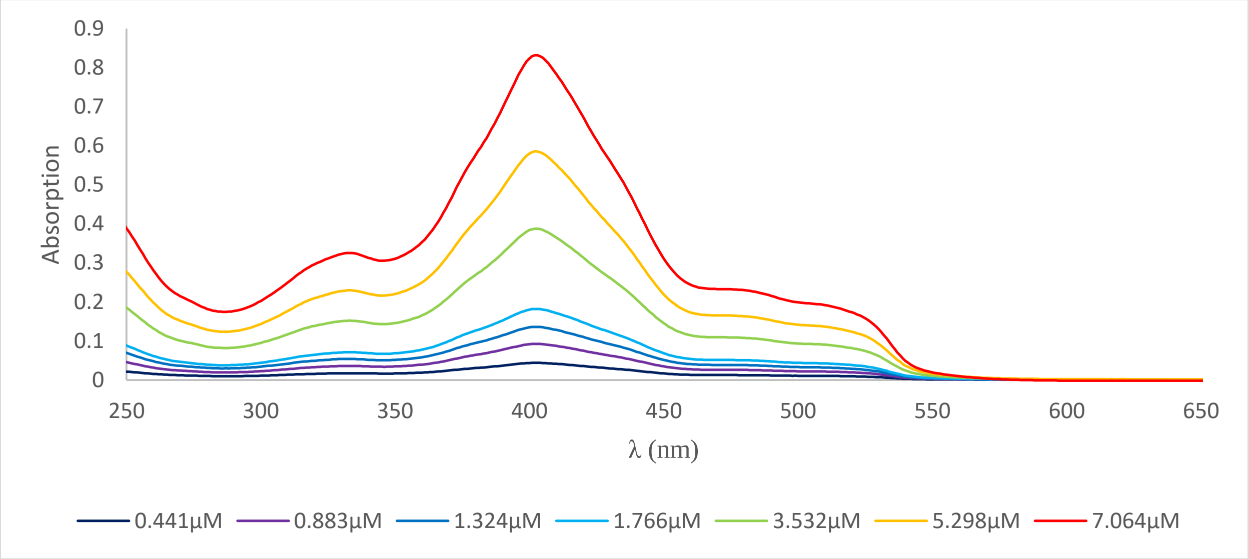

Figure S40. Absorption spectra of **C-4BFI** in chloroform in different concentrations.

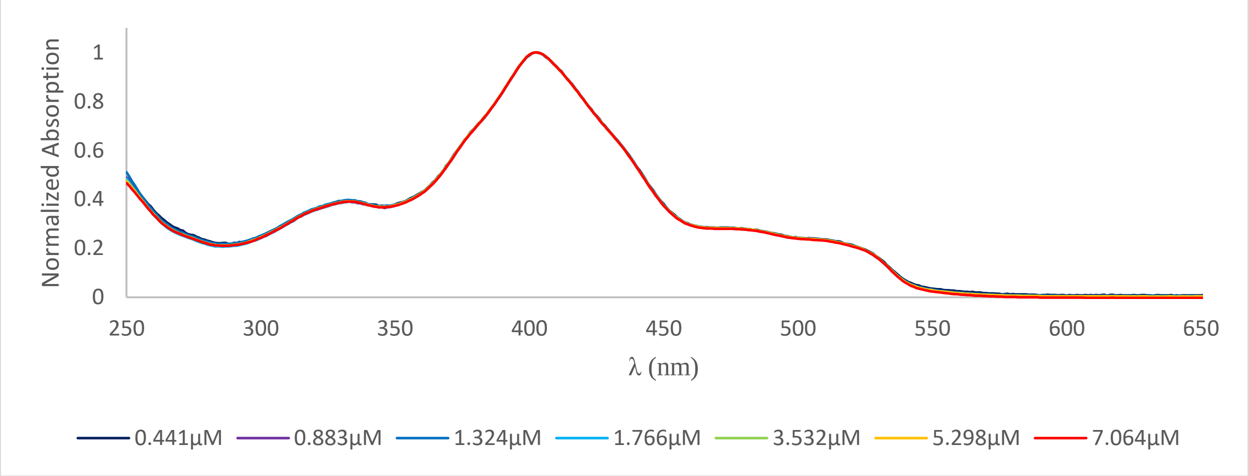

Figure S41. Normalized absorption spectra of **C-4BFI** in chloroform in different concentrations, showing no concentration dependence of the absorption spectra.

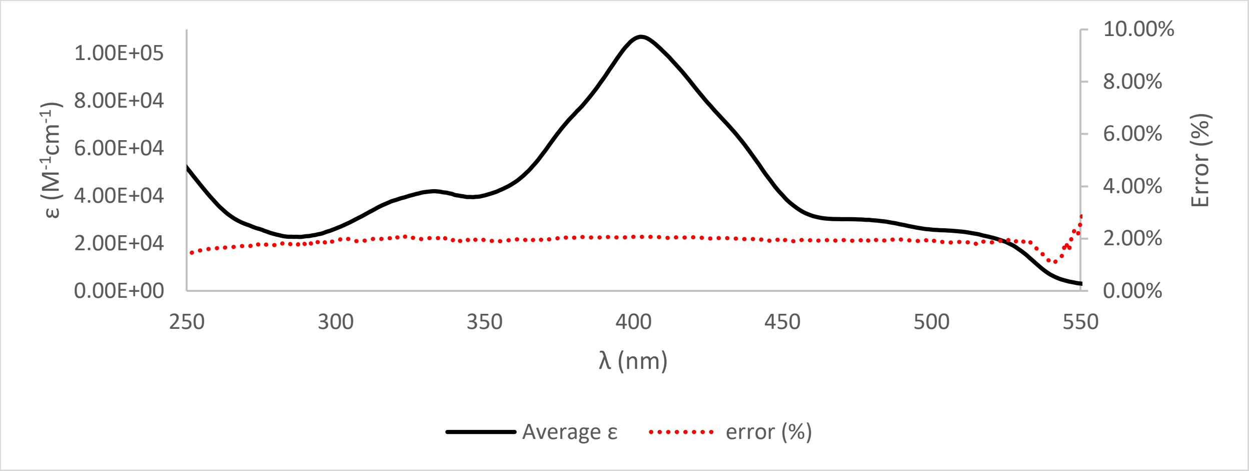

Figure S42. Extinction coefficient of **C-4BFI** in chloroform averaged from the measurements shown in Figure S40 (black) and the error for this average (red).

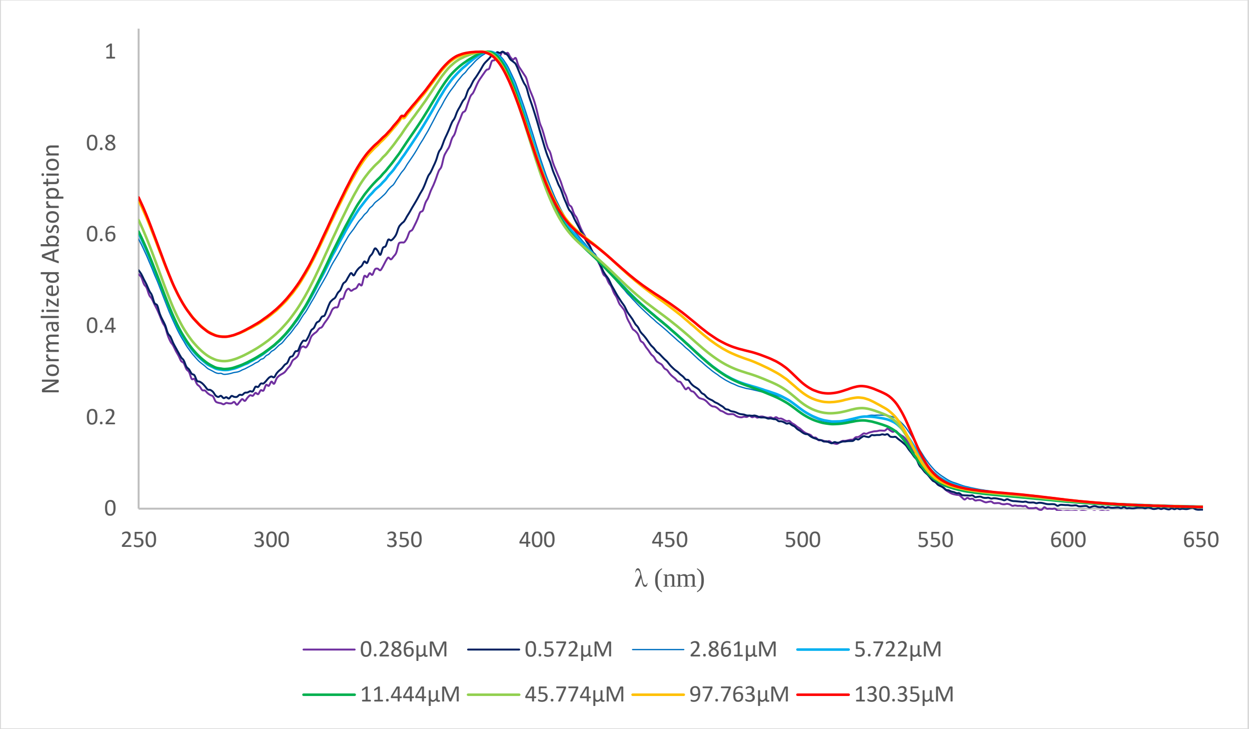

Figure S43. Normalized absorption spectra of **C-4BFI** in hexane. A hypsochromic shift is observed in λ_{max}, with a difference of 8nm between the λ_{max} values of the least concentrated solution and of the most concentrated one (0.286µM and 130.35µM respectively).

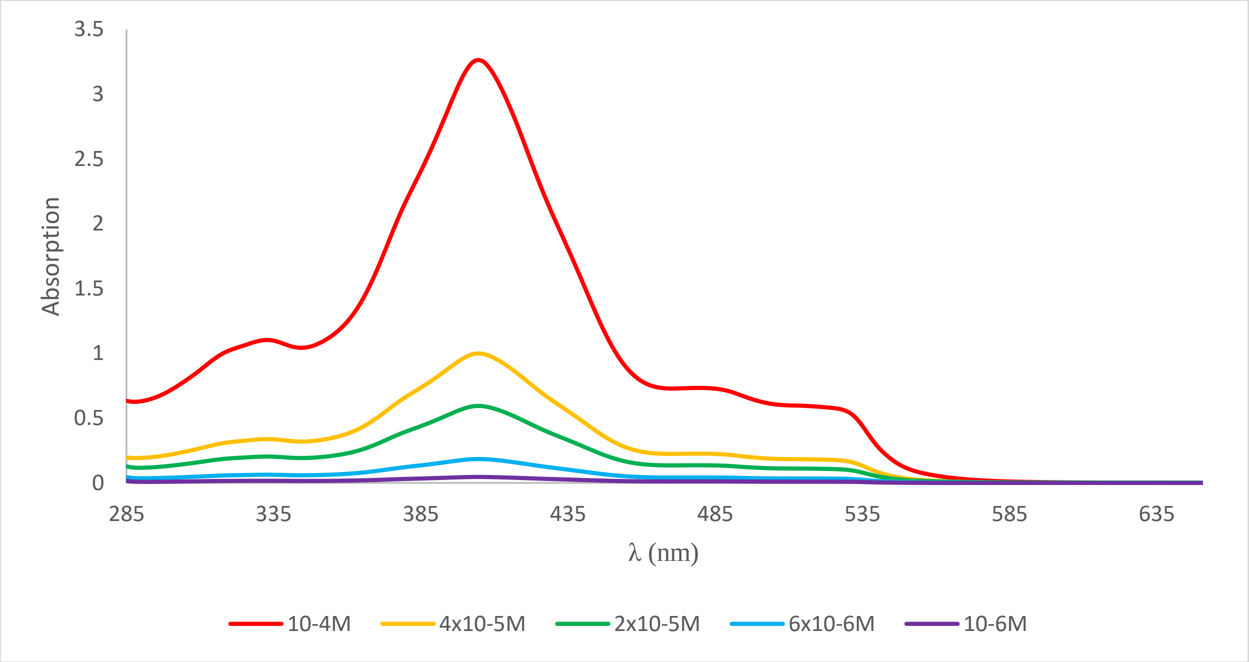

Figure S44. Absorption spectra of **C-4BFI** in toluene in different concentrations.

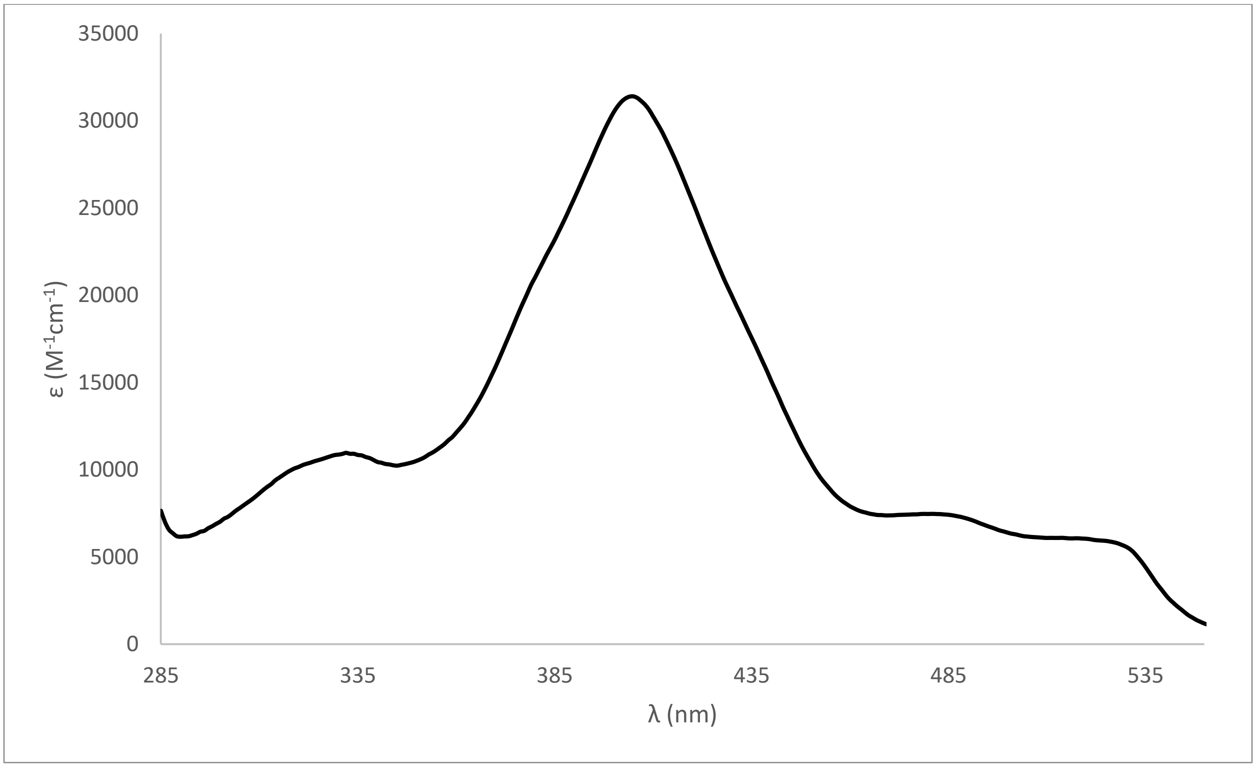

Figure S45. Extinction coefficient of **C-4BFI** in toulene averaged from the measurements shown in Figure S44.

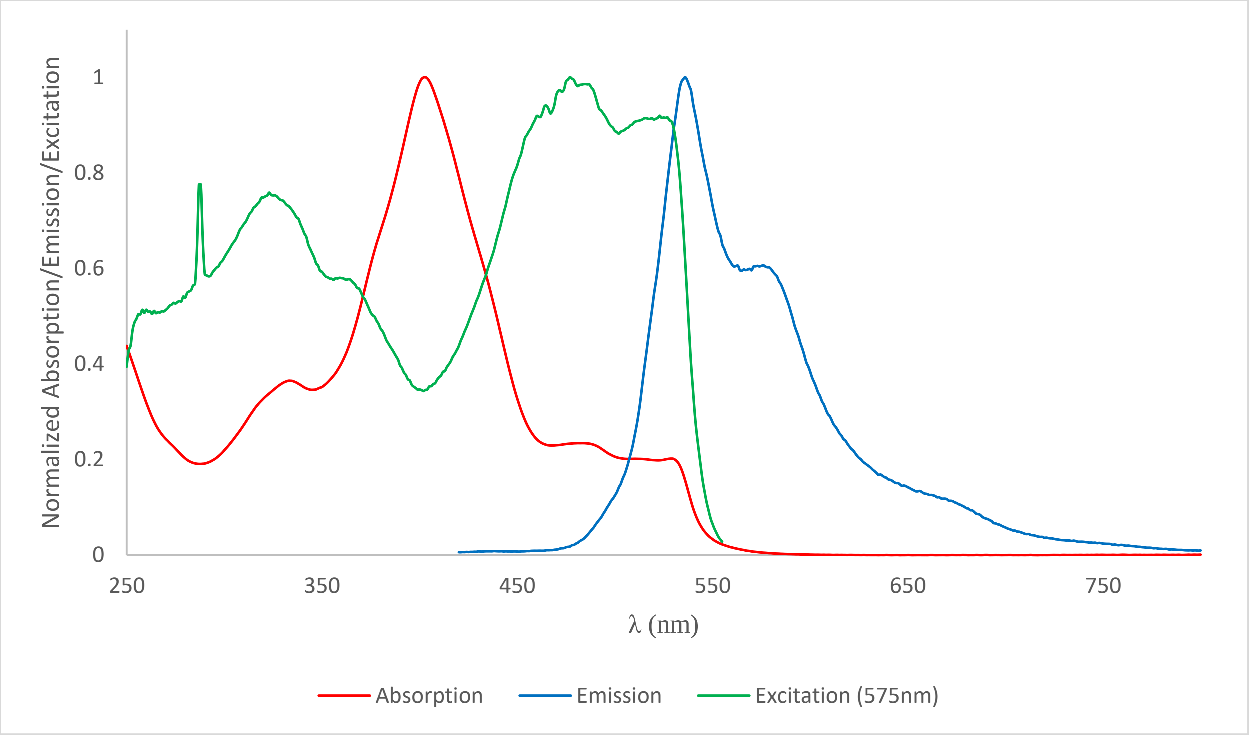

Figure S46. Absorption (red), emission (blue, excited at 402nm), and excitation (green, solid, emission at 575nm) spectra of **C-4BFI** in chloroform.

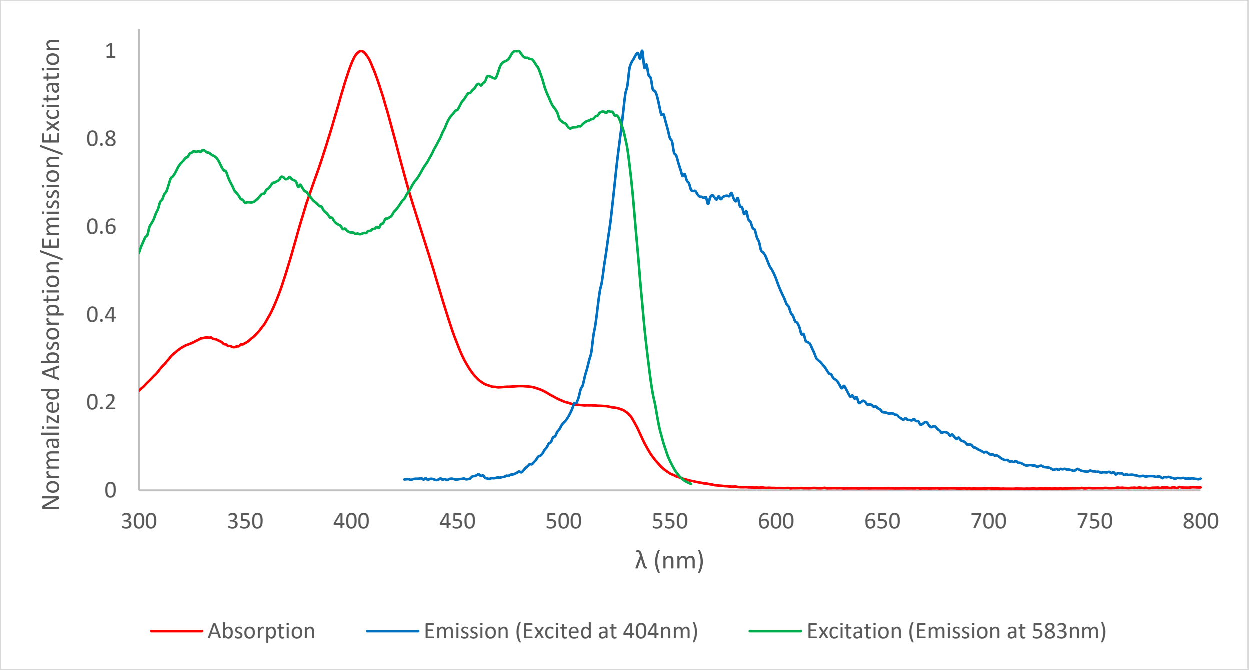

Figure S47. Absorption (red), emission (blue, excited at 404nm), and excitation (green, solid, emission at 583nm) spectra of **C-4BFI** in Toluene.

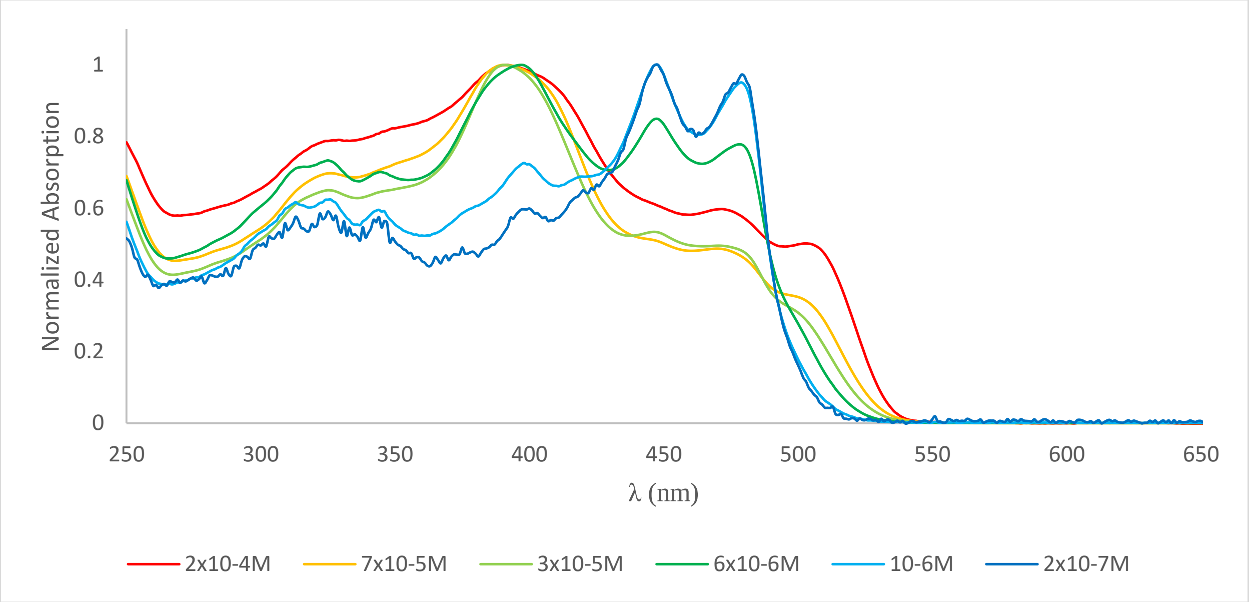

Figure S48. Normalized absorption spectra of **L-4BFI** in hexane.

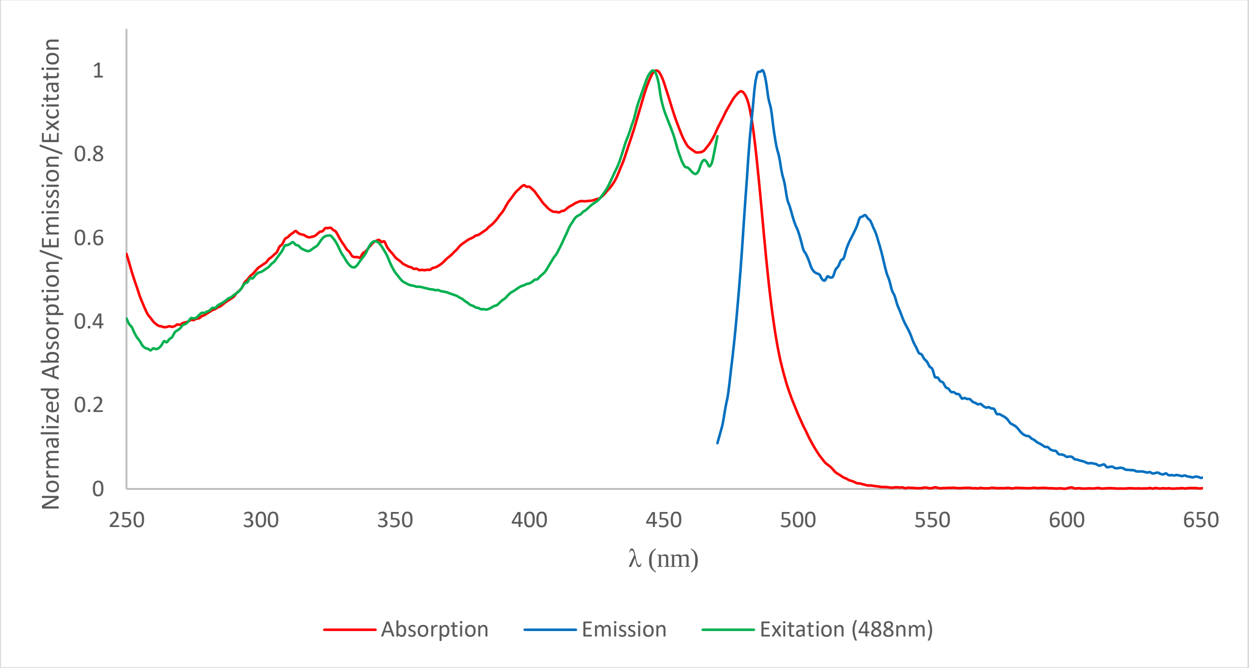

Figure S49. Absorption (red), emission (blue, excited at 448nm), and excitation (green, emission at 488nm) spectra of **L-4BFI** (1.127μM) in hexane.

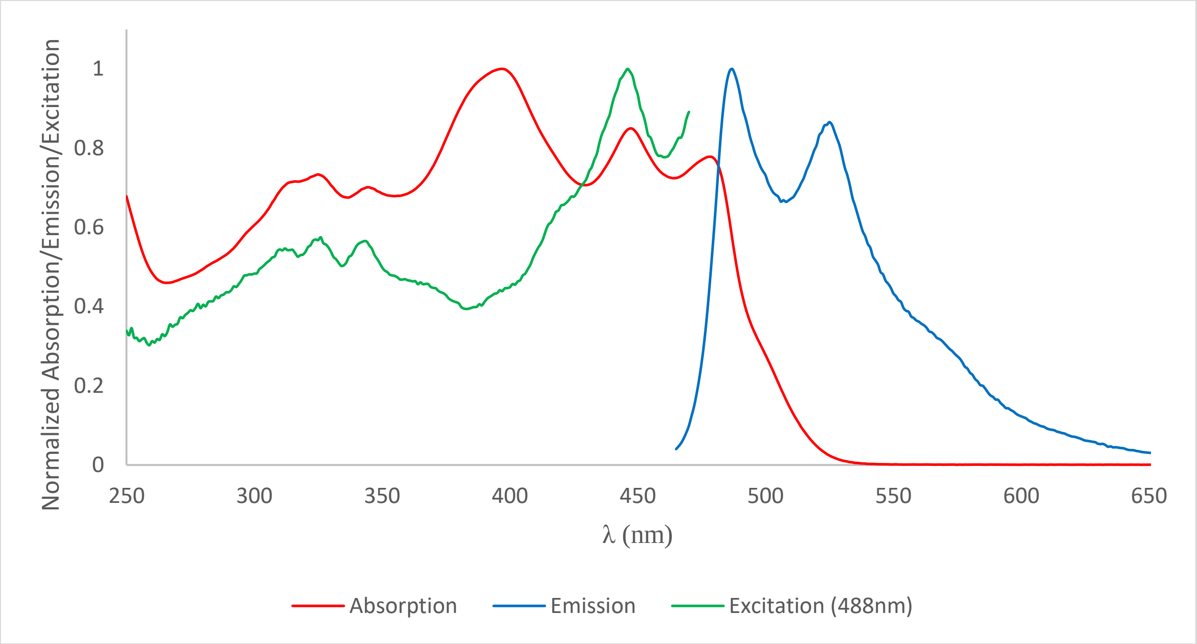

Figure S50. Absorption (red), emission (blue, excited at 447nm), and excitation (green, emission at 488nm) spectra of **L-4BFI** (5.633μM) in hexane.

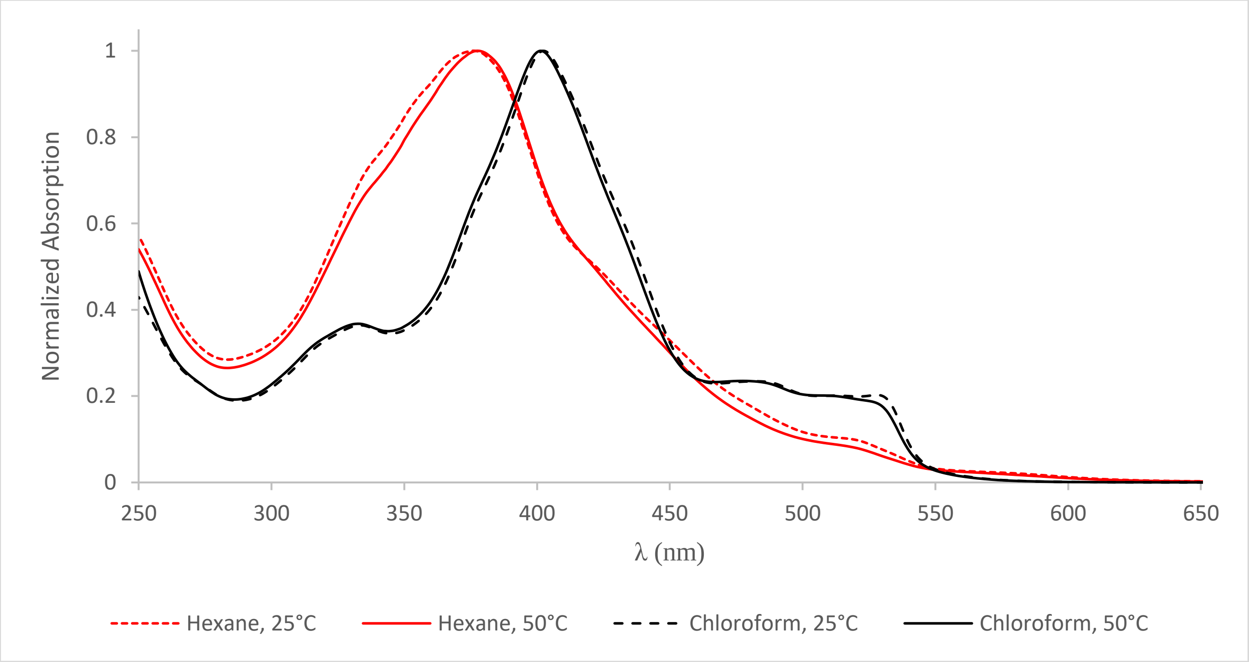

Figure S51. Absorption spectra for **C-4BFI** in chloroform at 25°C (black, dashed) and at 50°C (black, solid) and in hexane at 25°C (red, dashed) and at 50°C (red, solid).

S8. Aggregation NMR Studies

Increasing the macrocycle concentration affects the chemical ^{1}H-NMR chemical shift of **C-4BFI** in chloroform-*d*, indicating aggregation. The association constant was determined by measuring concentration dependent chemical shifts of the **C-4BFI** β-protons. Analysis was performed assuming monomer-dimer equilibrium is the predominant process, applying non-linear regression analysis:

$$\delta = \delta_0 + (\delta_\infty - \delta_0)\left(\frac{4KC_t + 1 - \sqrt{8KC_t + 1}}{4KC_t}\right)$$

Where δ is the observed chemical shift in a given total (constant) macrocycle concentration (C_t). δ_0 and δ_∞ are concentrations in infinite dilution and complete association, respectively. We note that while a good fit is observed for the µM to mM regime, at higher concentrations (>1 mM) the behavior deviates from the abovementioned model, and it is reasonable to assume that in higher concentrations the monomer-dimer equilibrium is not the predominant process but rather higher aggregates.

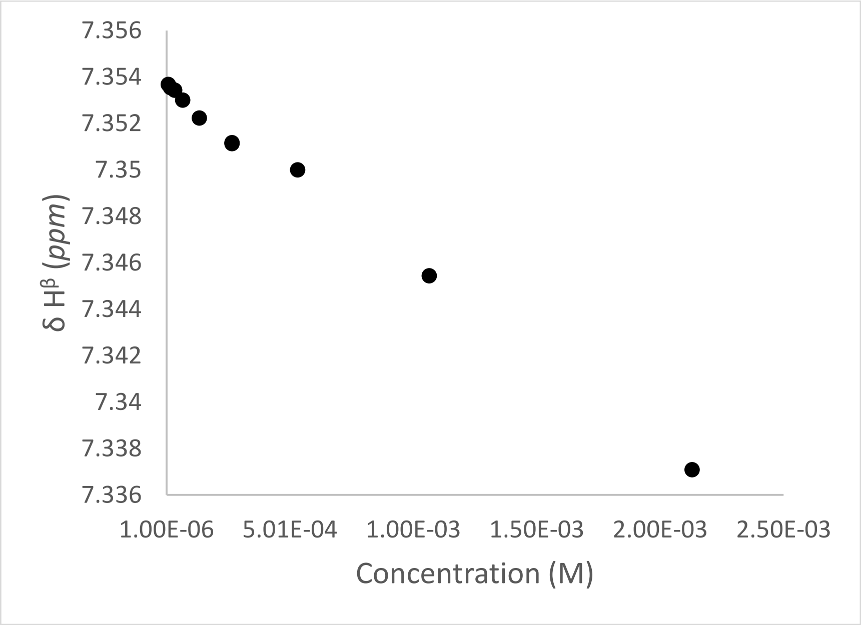

Figure S52. Chemical shift (ppm) of the aromatic β-proton of bifuranimide unit in **C-4BFI** measured in chloroform-*d* at different concentrations.

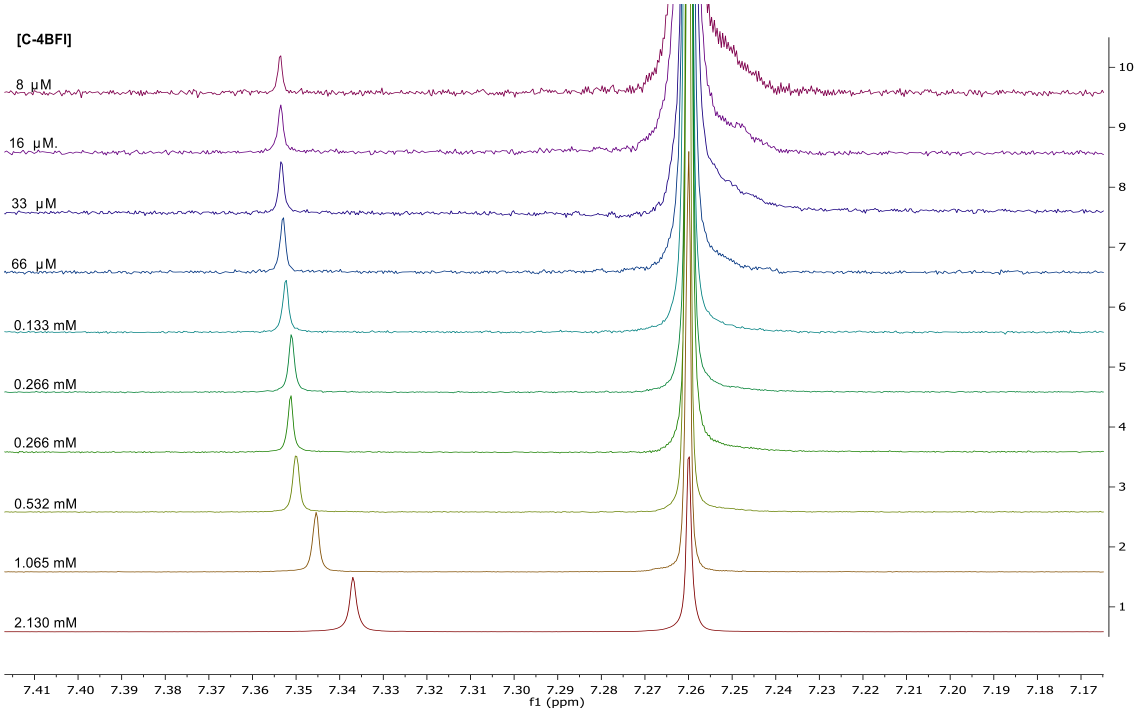

Figure S53. ^{1}H NMR of **C-4BFI** measured in chloroform-*d* at different concentrations.

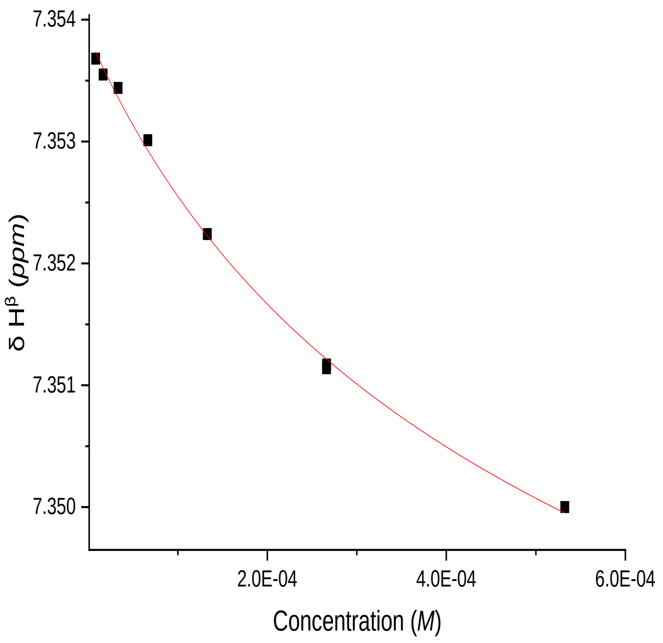

Figure S54. Fitting values and constants for a dimerization model for **C-4BFI** based on the data showed in Figures S52 and S53.

Equation[10, 11]	$\delta = \delta_0 + (\delta_\infty - \delta_0)\left(\frac{4KC_t + 1 - \sqrt{8KC_t + 1}}{4KC_t}\right)$
δ_∞	7.34 ± 0.001
δ_0	$7.35 \pm 5.996E - 5$
K	725 ± 134
Reduced Chi-Sqr	$5.804E - 9$
R-Square (COD)	0.9978
Adj. R-Square	0.99692

S9. Electrochemistry

For electrochemical measurements, dichloromethane containing 0.1M tetra-*n*-butylammonium perchlorate (TBAPC) was used as a solvent. Ag/AgCl was used as a reference electrode by dipping a silver wire in an aqueous solution of $FeCl_3$ and HCl. Platinum-disk and platinum-wire electrodes were applied as working and counter electrodes, respectively. All electrochemical measurements were performed under a dry nitrogen atmosphere and were externally calibrated against $E^{1/2}$ of the Fc/Fc^+ redox couple.

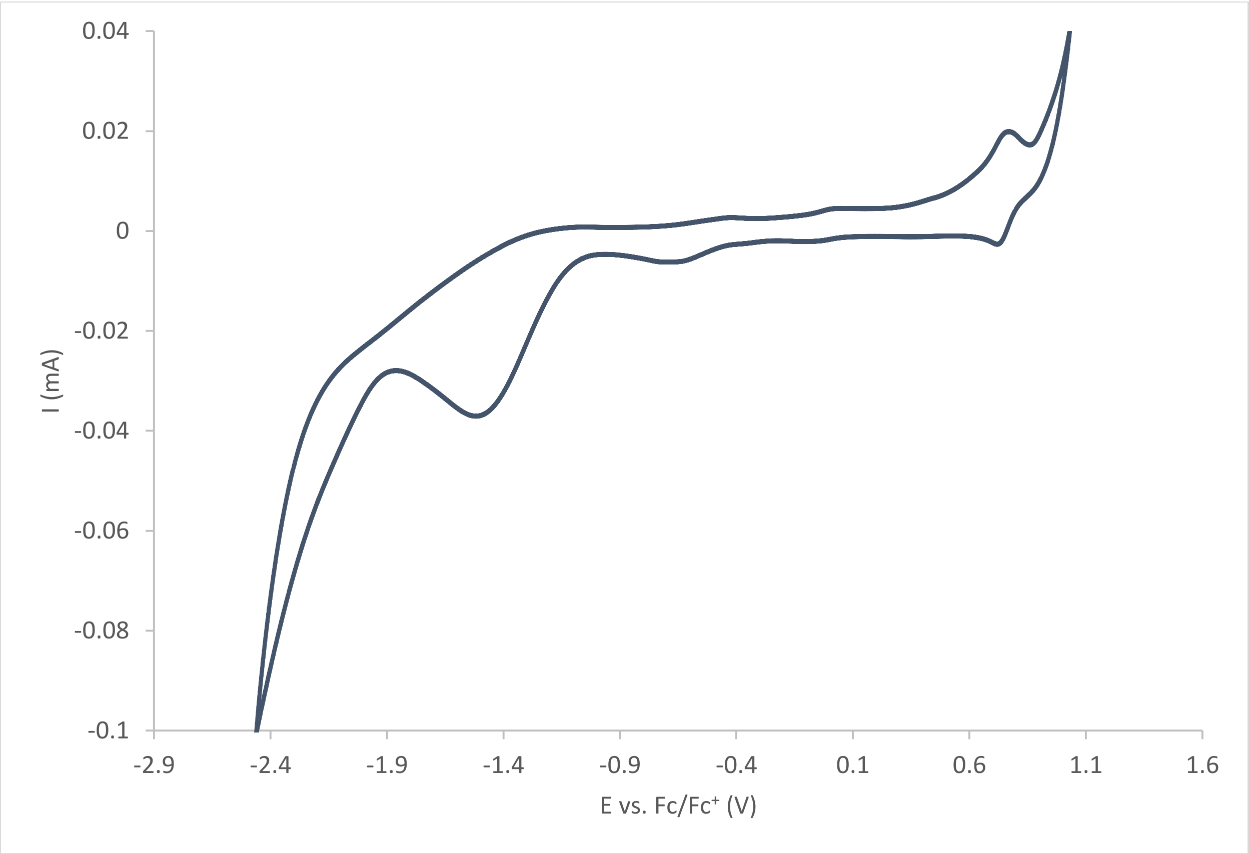

Figure S55. Cyclic voltammetry of **C-4BFI** in dichloromethane as solvent and 0.1M TBAPC as electrolyte, referenced against the Fc/Fc^+ redox couple (scan rate 100 mV/s).

S10. Bond Length Alternations

Bond length alternation (BLA) is an important criterion for aromaticity for conjugated systems.[12, 13] We depict here the BLA of selected **C-nBFI** and **C-4BTI** discussed in this work and compare them to the appropriate **nCX**. For **C-nBFI** and **C-4BTI** we recognize five different types of bonds: external double bonds (red), single bonds (green), internal double bonds (blue), internal interring bond (pink), and external interring bond (orange).

a) b)

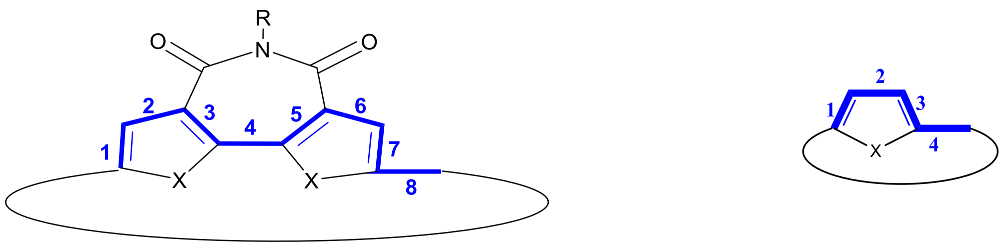

Chart S3. Bond numbering in (a) **C-nBFI** (X = O), **C-nBTI** (X = S), and (b) **nCF** (X = O), **nCT** (X = S).

The internal and external interring bond lengths (4 and 8, respectively) are depicted separately from the other bond lengths. The external interring bonds for both optimized structure and crystal structure are similar to the interring bond length of **8CF**; the internal interring bond are even shorter, which could suggest additional conjugation inside the **BFI** subunit.

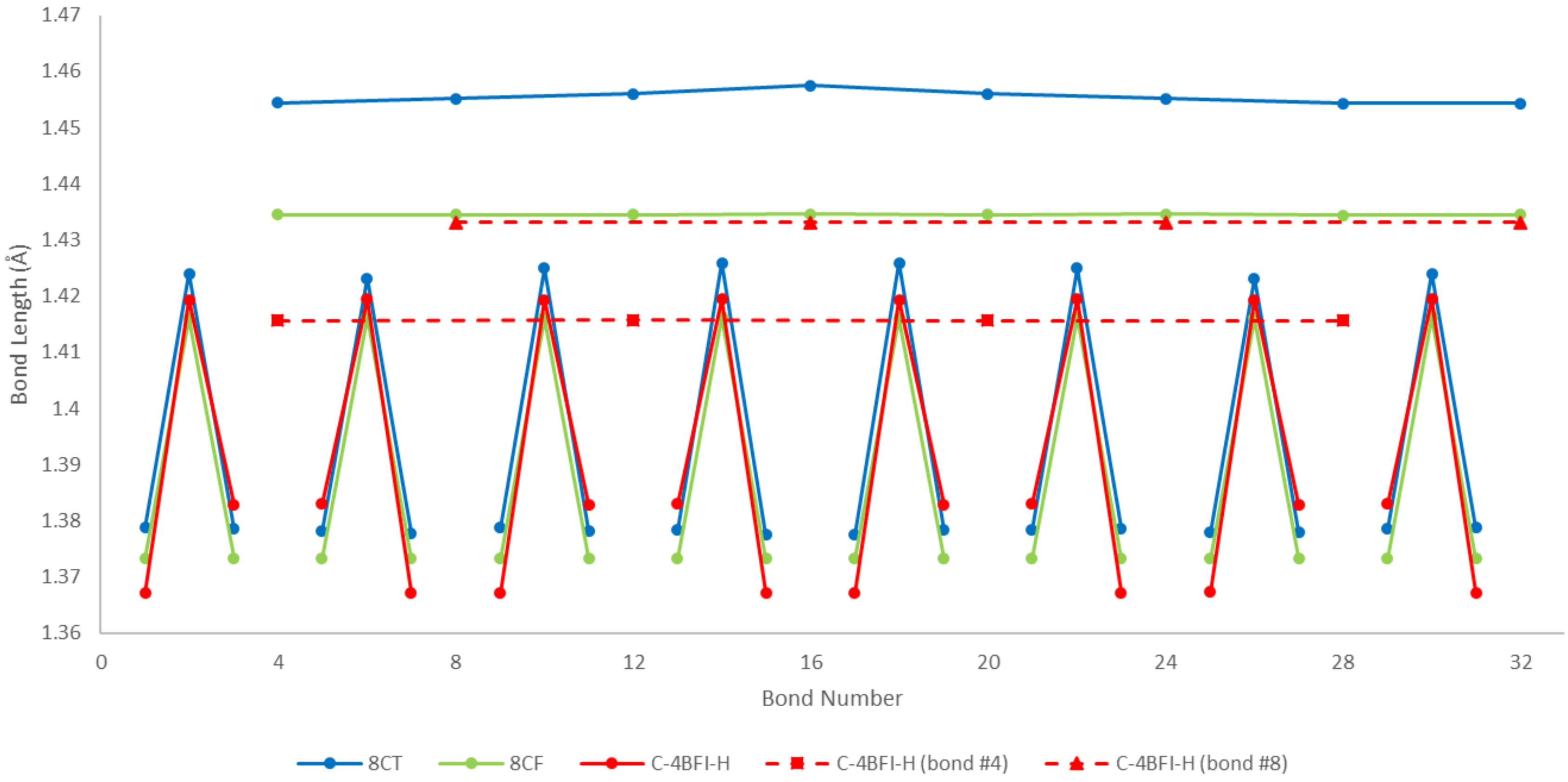

Figure S56. BLA pattern of **8CT**, **8CF**, and **C-4BFI-H**, based on the optimized structures (B3LYP/6-311G(d)).[9]

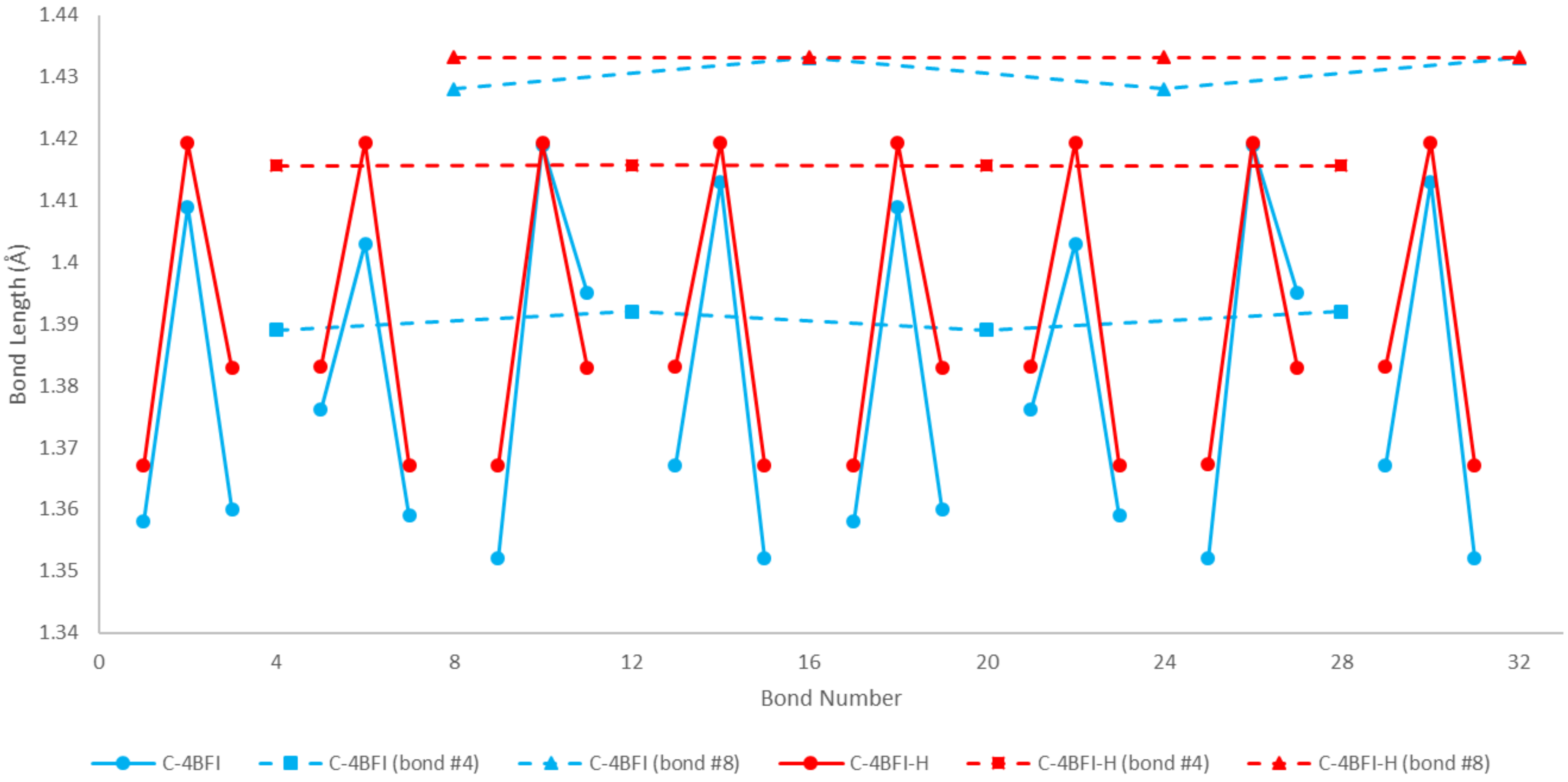

Figure S57. BLA pattern of **C-4BFI-H** (based on the optimized structures (B3LYP/6-311G(d))) and **C-4BFI** (based on the crystal structure).

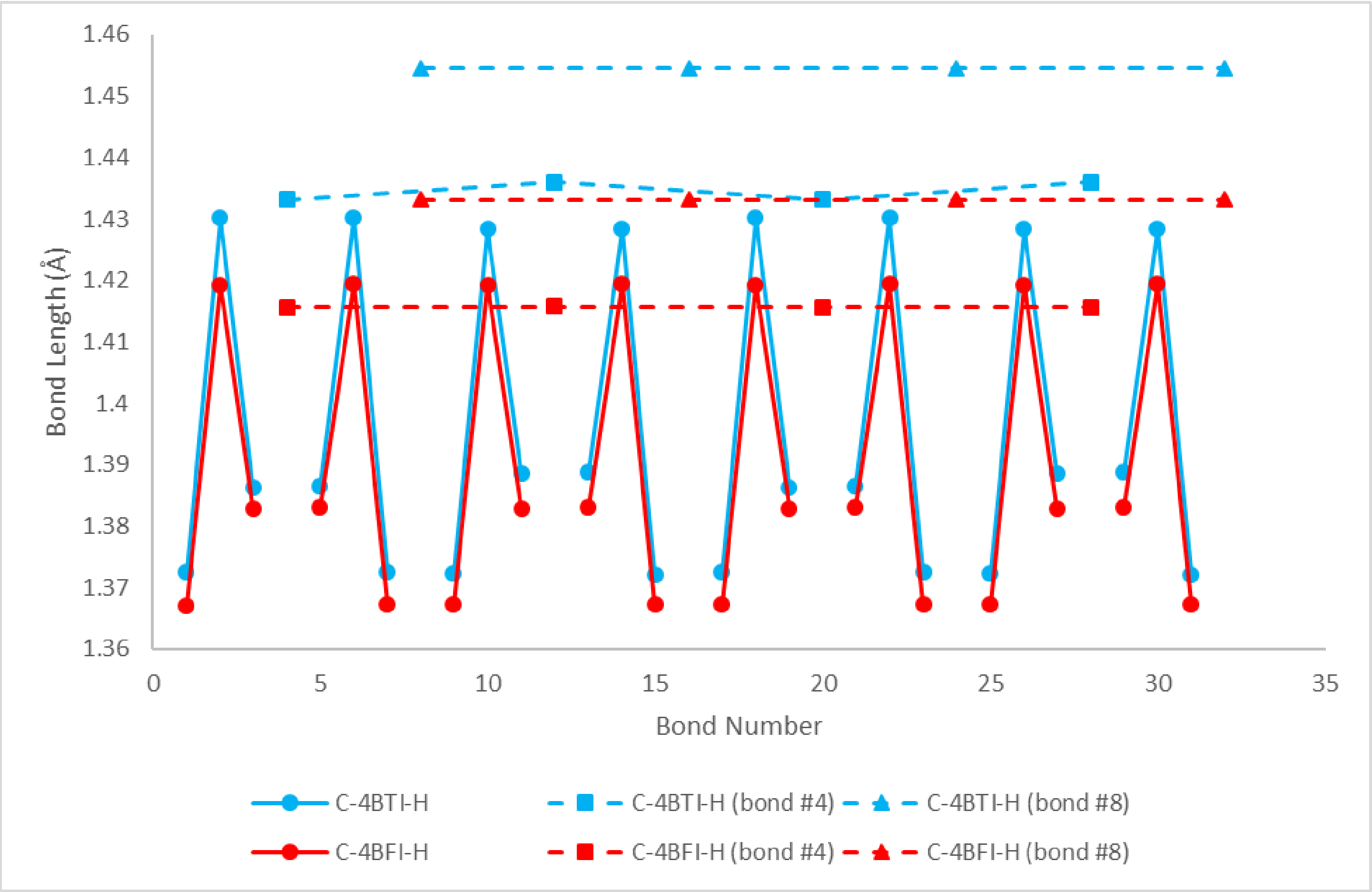

Figure S58. BLA pattern of **C-4BFI-H** and **C-4BTI-H** based on the optimized structures (B3LYP/6-311G(d)).

S11. Scanning Probe Microscopy measurements

STM experiments were carried out using a Nanoscope Multimode 8 with A-scanner and standard STM scanning head with the tip immersed in the supernatant liquid at room temperature. STM tips were prepared by mechanical cutting of Pt/Ir wire (80/20, diameter 0.25 mm). Prior to imaging, 6 µl of C-$_4$BFI saturated 1,2,4-trichlorobenzene (TCB) solution (Fisher Scientific) was applied on the basal (0001) plane of freshly cleaved highly ordered pyrolytic graphite (HOPG, grade ZYB, Bruker SFR). Imaging started 1 minute after the drop casting. The self-assembled molecular network was visualized at the liquid-solid interface in about half an hour. Several samples were investigated, and for each sample several locations were probed. The imaging parameters I_{set} (tunneling current) and V_{bias} (substrate bias) are described in the figure captions. The substrate bias was negative, indicating a tunneling current from the substrate to the STM tip. For analysis purposes, recording of a molecular network image was followed by imaging the graphite substrate underneath it under the same experimental conditions, except for lowering the bias. The images were corrected for drift via WSxM software[14] using the recorded graphite images for calibration purposes, allowing a more accurate unit cell determination.

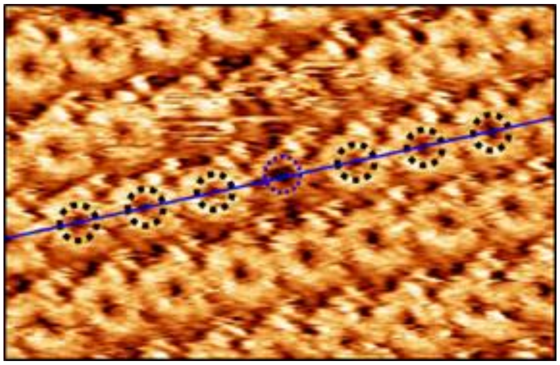

Figure S59. STM image of **C-4BFI** multilayer at liquid-solid interface on HOPG. The molecule of the bottom layer is visible through the vacancy defect. Its positions (blue dotted circle) appears slightly shifted vs the blue line passing through the center of molecules in the upper layer (black dotted circle). Such ‘offset’ is expected based on the molecular packing determined by X-ray crystallography in 3D crystals, but its magnitude is too small to be determined by STM.

AFM measurement were carried out at ambient conditions on Nanoscope Multimode 8 equipped with a Nanoscope V controller (Bruker, Santa Barbara, CA). The topographies were acquired in PeakForce mode (BrukerScanAsyst mode and Nanoscope 8.15r3 software) on the Nanoscope Multimode 8. WSxM software[8] was used to perform the particle size analysis. Sample preparation for AFM measurements was performed as follows. A diluted solution of **C-4BFI** (1×10^{-5} M in hexane) was heated to 60 °C to solubilize all possible aggregates. A freshly cleaved mica substrate was placed in the solution, which were kept in a closed vial overnight. Before imaging, the substrate was picked out of the solution and blow dried with nitrogen.

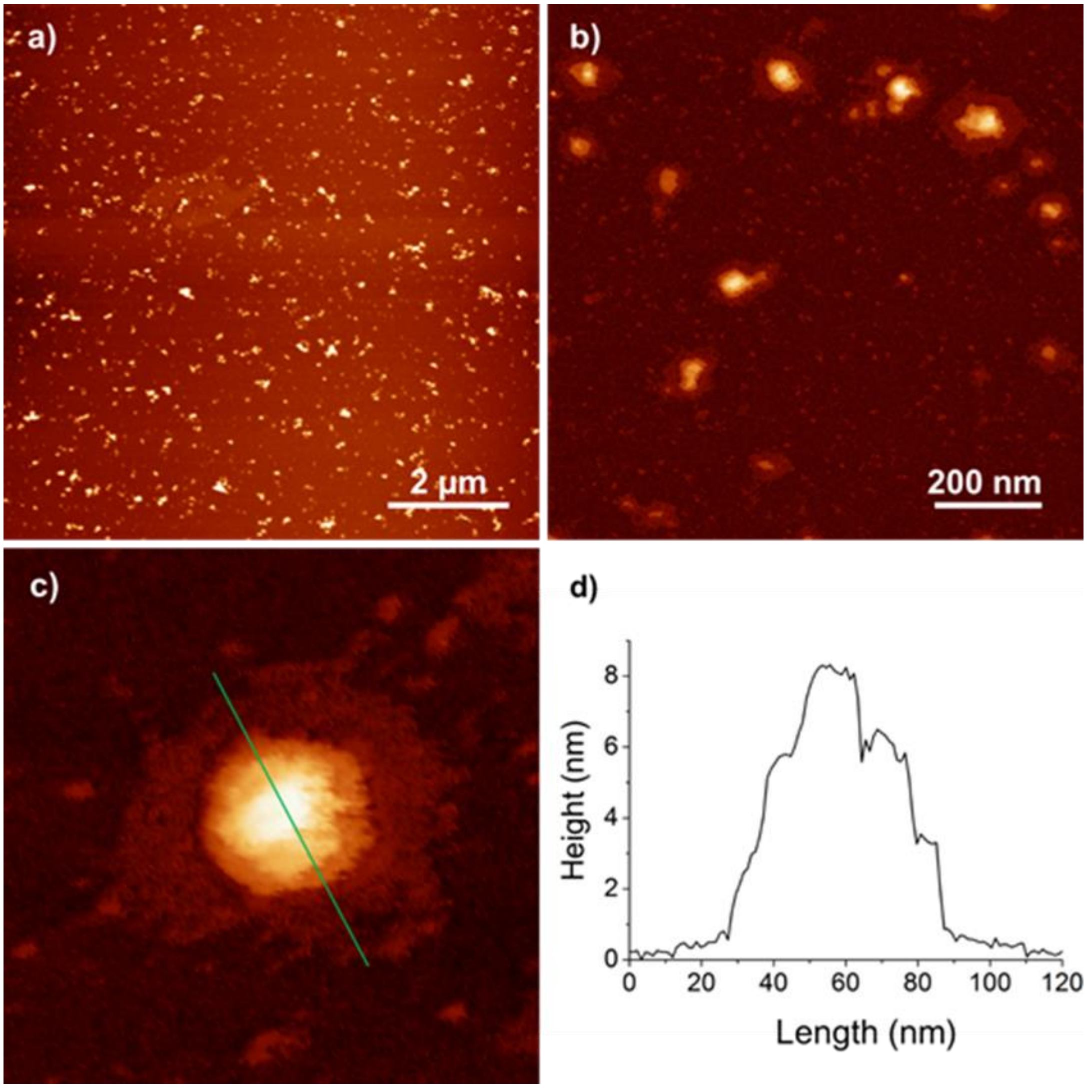

Figure S60. C-4BFI particles adsorbed on mica from 1×10^{-5} M hexane solution. (a & b) AFM topographic images; c) a zoom-in AFM image of an individual particle step edges which suggest its crystallinity; d) AFM height profiles corresponding to the green line in (c). The observed step-edge of ~3 nm suggests edge-on orientation of the molecules.

S12. Dynamic Light Scattering (DLS)

DLS experiments were carried out using a DynaPro™ S10 Instrument from Wyatt Technology. A cumulants fit model was used to confirm the presence and determine the size the aggregates. Samples were prepared by dissolving **C-4BFI** in hexane heating at 60 °C, leaving at room temperature overnight and filtering through a 0.2 μm syringe filter before transferring into DLS measuring cell. All measurements were carried out at 21 °C

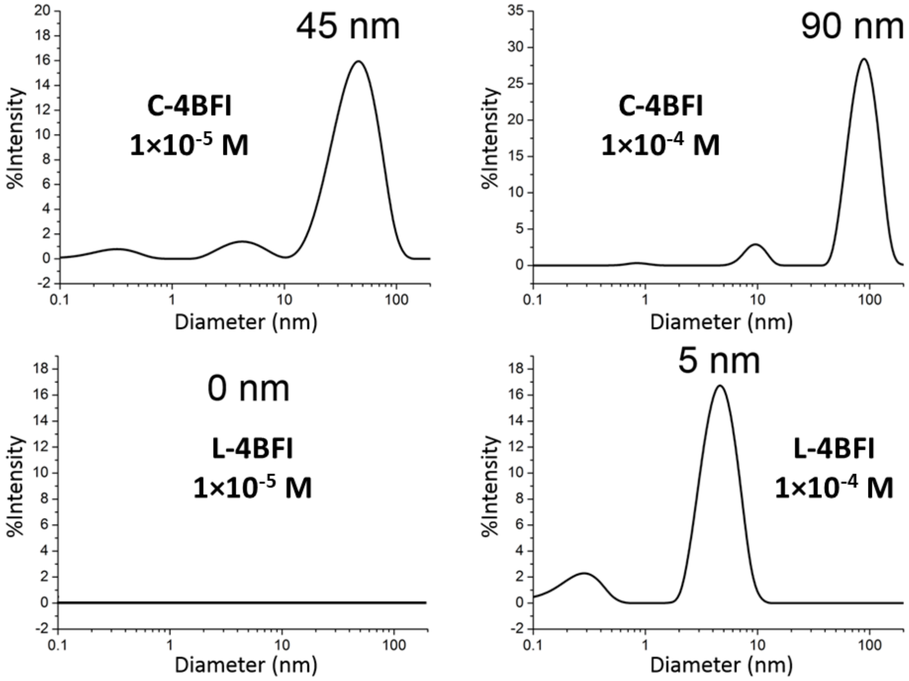

Figure S61. Dynamic light scattering (DLS) of **C-4BFI** and **L-4BFI** in hexane.

S13. Device Fabrication

The diodes were fabricated on Si/SiO_2 substrates thoroughly cleaned with acetone, 2-propanol, deionized water and a basic-piranha solution (deionized water: hydrogen peroxide: ammonium hydroxide (5:1:1)) and subjected to 5-minute oxygen plasma treatment. The bottom & top electrodes were defined by thermal evaporation using shadow masks resulting in device area of 0.0002 cm^2. The FETs were fabricated using Fraunhofer bottom contact-bottom gate substrates. The substrates were rinsed with acetone, 2-propoanl and subjected to oxygen-plasma cleaning before modifying the workfunction of gold electrodes with pentaflourobenzenethiol (PFBT) self-assembled monolayer. The c-4BFI was spin coated from 5mg/ml solution in chlorobenzene. Thin films were annealed at 70ºC for 2 minutes to remove any residual solvent. All the devices were characterized using Kiethley 4200 SCS system. We did not notice any difference in the current-voltage characteristics by performing the measurements in air or under vacuum. The detailed device architectures are shown in Figure S60.

We used Mott-Gurney's law to extract μ_{SCLC} and is given by Equation 1.

$$J = \frac{9\mu_{SCLC}\varepsilon_0\varepsilon_r V^2}{8L^3} \qquad (1)$$

Where where ε_0 and ε_r are the vacuum and relative dielectric permittivities, L is the thickness of c-4BFI spin coated layer (200nm), J is the current density, V is the applied voltage and μ is the material's carrier mobility in SCLC regime.

The saturation field effect mobility was determined using the following Equation 2.

$$\mu_{sat} = \frac{2L}{WC_i}\frac{\partial^2 {I_{DS}}^{sat}}{\partial {V_{GS}}^2} \qquad (2)$$

Where L is channel length (2.5 μm), W is channel width (10 mm), C_i is dielectric capacitance (SiO_2 240 nm = 15 $nFcm^{-2}$).

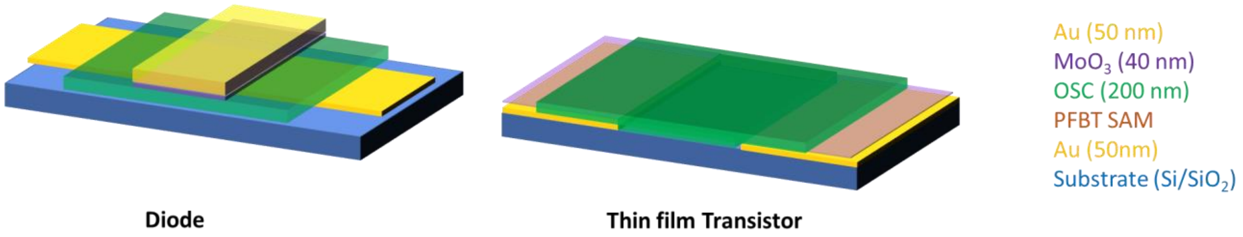

Figure S62. The schematic representation of device architectures.

References

1. S. V. Mulay, B. Bogoslavky, I. Galanti, E. Galun and O. Gidron, *J. Mater. Chem. C*, 2018, **6**, 11951-11955.
2. Gaussian 09, Revision D.01, M. J. Frisch, G. W. Trucks, H. B. Schlegel, G. E. Scuseria, M. A. Robb, J. R. Cheeseman, G. Scalmani, V. Barone, G. A. Petersson, H. Nakatsuji, X. Li, M. Caricato, A. Marenich, J. Bloino, B. G. Janesko, R. Gomperts, B. Mennucci, H. P. Hratchian, J. V. Ortiz, A. F. Izmaylov, J. L. Sonnenberg, D. Williams-Young, F. Ding, F. Lipparini, F. Egidi, J. Goings, B. Peng, A. Petrone, T. Henderson, D. Ranasinghe, V. G. Zakrzewski, J. Gao, N. Rega, G. Zheng, W. Liang, M. Hada, M. Ehara, K. Toyota, R. Fukuda, J. Hasegawa, M. Ishida, T. Nakajima, Y. Honda, O. Kitao, H. Nakai, T. Vreven, K. Throssell, J. A. Montgomery, Jr., J. E. Peralta, F. Ogliaro, M. Bearpark, J. J. Heyd, E. Brothers, K. N. Kudin, V. N. Staroverov, T. Keith, R. Kobayashi, J. Normand, K. Raghavachari, A. Rendell, J. C. Burant, S. S. Iyengar, J. Tomasi, M. Cossi, J. M. Millam, M. Klene, C. Adamo, R. Cammi, J. W. Ochterski, R. L. Martin, K. Morokuma, O. Farkas, J. B. Foresman, and D. J. Fox, Gaussian, Inc., Wallingford CT, 2016.
3. W. Koch and M. C. Holthausen, *A chemist's guide to density functional theory*, Wiley-VCH, 2000.
4. A. D. Becke, *J. Chem. Phys.*, 1993, **98**, 5648-5652.
5. R. G. Parr and Y. Weitao, *Density-Functional Theory of Atoms and Molecules*, Oxford University Press, 1989.
6. C. Lee, W. Yang and R. G. Parr, *Phys. Rev. B*, 1988, **37**, 785-789.
7. J. Fabian and H. Hartmann, *J. Phys. Org. Chem.*, 2007, **20**, 554-567.
8. S. S. Zade and M. Bendikov, *J. Org. Chem.*, 2006, **71**, 2972-2981.
9. O. Dishi and O. Gidron, *J. Org. Chem.*, 2018, **83**, 3119-3125.
10. S. Höger, K. Bonrad, A. Mourran, U. Beginn and M. Möller, *J. Am. Chem. Soc.*, 2001, **123**, 5651-5659.
11. R. B. Martin, *Chem. Rev.*, 1996, **96**, 3043-3064.
12. J. L. Bredas, *J. Chem. Phys.*, 1985, **82**, 3808-3811.
13. V. M. Geskin, A. Dkhissi and J. L. Brédas, *Int. J. Quantum Chem*, 2003, **91**, 350-354.
14. I. Horcas, R. Fernández, J. M. Gómez-Rodríguez, J. Colchero, J. Gómez-Herrero and A. M. Baro, *Rev. Sci. Instrum.*, 2007, **78**, 013705.
